# The spatiotemporal dynamics of lung cancer: 30-year trends of epidemiology across 204 countries and territories

**DOI:** 10.1186/s12889-022-13281-y

**Published:** 2022-05-16

**Authors:** Xiang Chen, Shaoyan Mo, Bin Yi

**Affiliations:** 1 Department of Anesthesiology, Xiangya Hospital, Central South University, Changsha, 410008 Hunan People’s Republic of China; 2 National Clinical Research Center for Geriatric Disorders, Xiangya Hospital, Central South University, Changsha, 410008 Hunan People’s Republic of China; 3 Department of Thoracic Surgery, Xiangya Hospital, Central South University, Changsha, 410008 Hunan People’s Republic of China; 4 Hunan Engineering Research Center for Pulmonary Nodules Precise Diagnosis & Treatment, Changsha, 410008 Hunan People’s Republic of China; 5 Xiangya Lung Cancer Center, Xiangya Hospital, Central South University, Changsha, 410008 Hunan People’s Republic of China

**Keywords:** Lung cancer, Prevalence, Incidence, Years lived with disability, Spatiotemporal dynamics

## Abstract

**Background:**

It has been established that lung cancer is the leading cause of all cancer deaths. This study sought to analyze the epidemiological trends of lung cancer over the past 30 years worldwide.

**Methods:**

Estimates, including the global, regional, national prevalence, incidence, and years lived with disability (YLDs) of lung cancer from 1990 to 2019, were extracted from the Global Burden of Disease Study 2019 to assess the spatiotemporal dynamics in cases and age-standardized rates (ASR). The estimated annual percentage change (EAPC) was calculated to evaluate the variation in ASR. Besides, estimates of age-sex specific prevalence, decomposition analysis for incident cases, and correlation analysis of the EAPC were conducted in our study.

**Results:**

Globally, the ASR of lung cancer prevalence, incidence and YLDs in 2019 were 38.84/100,000 persons, 27.66/100,000 persons, and 6.62/100,000 persons, respectively. Over the past 30 years, the ASR of incidence (EAPC = -0.09) decreased, although that of prevalence (EAPC = 0.51) and YLDs (EAPC = 0.03) increased. The global prevalence counts was greater in males than females at all age groups and increased with age, peaking in the 65–69 age group for both sexes. The increase in incidence was mainly attributed to population aging. For YLDs, EAPC was negatively correlated with the human development index (*p* = 0.0008) and ASR (*p* < 0.0001) in 1990 across nation-level units.

**Conclusions:**

Lung cancer remains a major public health issue globally, warranting the implementation of scientific and effective measures in different countries and territories to control it.

**Supplementary Information:**

The online version contains supplementary material available at 10.1186/s12889-022-13281-y.

## Background

Lung cancer remains the leading cancer killer, with 1,796,144 deaths reported in 2020, accounting for about 18% of all cancer deaths globally [1]. Most patients who suffered from advanced lung cancer ultimately experience local invasion and metastatic disease [2], which are often accompanied by heavy financial burden and poor quality of life [3]. Given the fatality and disability rates, more resources and emphasis should be placed on lung cancer. Accordingly, fully understanding the burden of lung cancer has significant value for implementing targeted therapeutic and protective programs, and facilitates the optimal allocation of limited resources.

The Global Burden of Disease Study (GBD) provides an overview of the burden from major diseases, injuries and risk factors to health at global, national and regional levels [4–6]. In this study, we sought to analyze the prevalence, incidence and years lived with disability (YLDs) of lung cancer from the GBD 2019, and evaluate the spatiotemporal dynamics of lung cancer by analyzing the trends from 1990 to 2019 at the global, regional, and national levels. Our findings can be a supplement and an extension to existing studies [7], and provide the foothold to guide global-, regional-and national-specific health care plans for lung cancer.

## Methods

### Study data

The prevalence, incidence and YLDs of lung cancer by year, sex and location, were acquired from the GBD 2019 [4–6], which made a systematic analysis of disease burden to all World Health Organization (WHO) member states, including a comprehensive assessment on 87 risk factors, 369 diseases and injuries from 1990 to 2019 in 204 countries and territories [8]. Estimates were available from 204 countries and territories, which were grouped into 21 GBD regions, such as East Asia. Furthermore, the globe was divided into five regions based on the sociodemographic index (SDI), a comprehensive indicator that estimates total fertility rate, lag-distributed income per capita, and average educational attainment population over age 15 years [9].

Previous studies have reported methodological details for the GBD 2019 and assessments of disease burden in lung cancer [5, 7]. The prevalence was quartered into different cancer phases: the diagnosis or treatment stage, the remission stage, the metastatic or disseminated stage and the terminal stage. The Cause of Death Ensemble model (CODEm) was used to estimate fatal lung cancer mortality. Once the final mortality estimates were obtained, the incidence of lung cancer was calculated according to the mortality-to-incidence ratio. YLDs, a scientific indicator representing the nonfatal disability, were calculated by multiplying each health state prevalence by the health state disability weight.

### Data identification and retrieval

Lung cancer was identified using the International Classification of Diseases codes, Tenth Revision and Ninth Revision (ICD-10 and ICD-9, respectively). Diseases coded as C33, C34–C34.92, Z12.2, Z80.1–Z80.2, Z85.1–Z85.20 in the ICD-10 and 162–162.9, 209.21, V10.1–V10.20, V16.1–V16.2, V16.4–V16.40 in the ICD-9 were attributed to lung cancer in the present study [7]. The following information about lung cancer were retrieved from the GBD 2019 (http://ghdx.healthdata.org/gbd-resultstool): population, prevalence, incidence, and YLDs by age and sex at the global, regional, and national levels from 1990 to 2019. In addition, we searched the World Bank for human development index (HDI), a measure used by the United Nations, consisting of three components: life expectancy, average income per person, and level of education [10].

### Statistical analysis

In addition to the absolute number and rate/100,000 persons, we also applied the age-standardized rate (ASR)/100,000 persons, including age-standardized prevalence rate (ASPR), age-standardized incidence rate (ASIR) and age-standardized YLDs rate (ASYR), given the heterogeneity in the age structure of population. Estimated annual percentage change (EAPC) was used to estimate the amplitude of ASR variations during the given period. The EAPC and its 95% confidence interval (CI) could also be calculated from a linear model that determined the logarithm of the ASR, that is, y = α + βx + ε. The ASR exhibited a downward or upward trend when the EAPC was less or greater than 0, respectively [11].

After classifying into 20 different age groups, the crude number and prevalence rates were collected to analyze the age-and sex-specific patterns for males and females in 2019. In addition, to explore the roles of population growth, population aging and changes in lung cancer burden per capita on the change in total lung cancer incidence cases, a decomposition analysis was performed by (1) applying the 1990 age-specific rates to the age structures and total population in 2019, and (2) applying the age-specific rates and age structures in 1990 to 2019 population size [12]. Furthermore, to assess the effect factors for the EAPC, we comprehensively analyzed the correlation of EAPC in ASYR with HDI and ASYR at the national level. All statistics and visualizations were generated by R software version 3.6.3 and GraphPad Prism 7. A *p*-value < 0.05 was statistically significant.

## Results

### Lung cancer prevalence

**Fig. 1:**
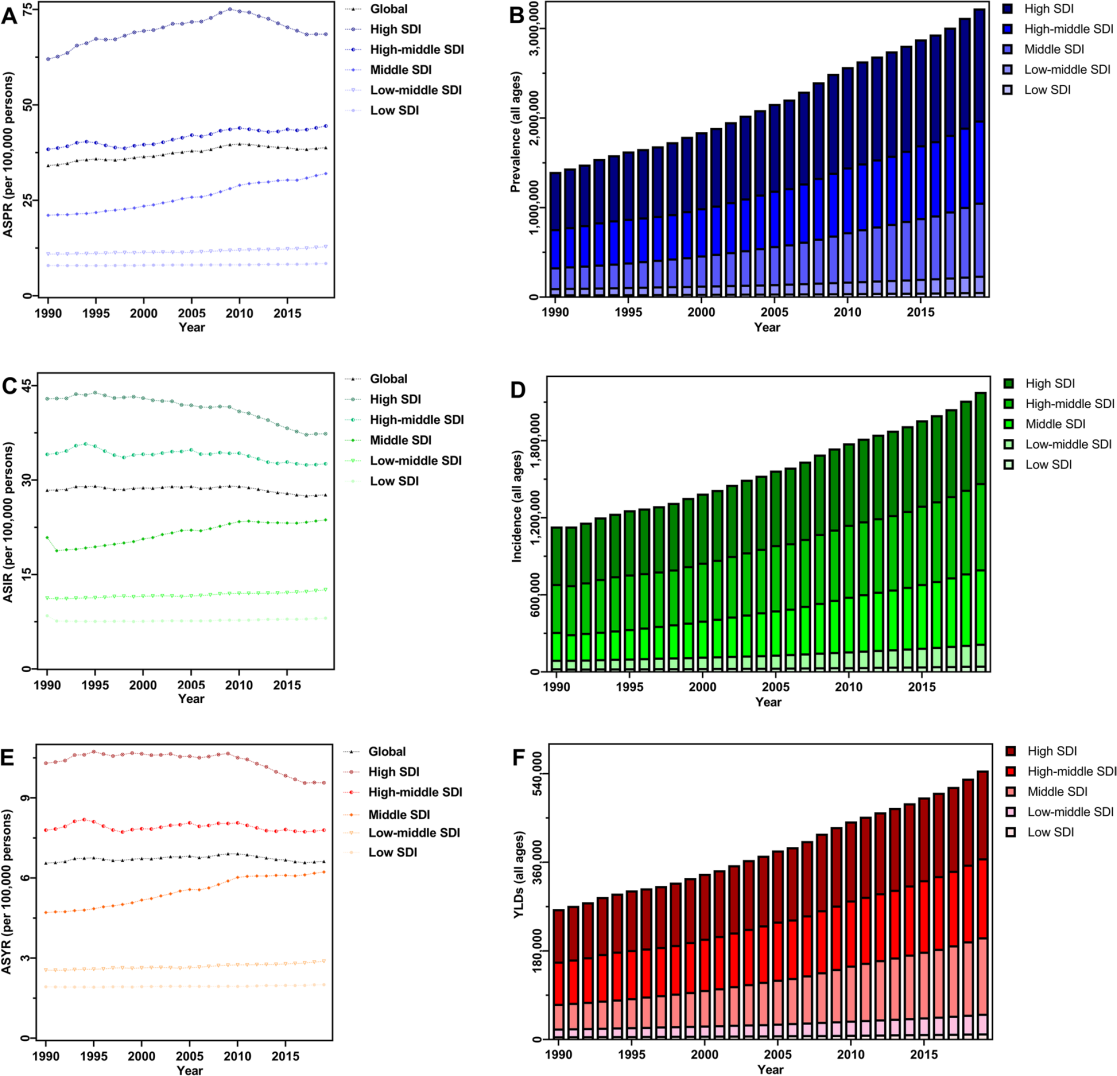
The epidemiology of lung cancer globally, and at five SDI regions from 1990 to 2019. (**A**) prevalent cases; (**B**) ASPR; (**C**) incident cases; (**D**) ASIR; (**E**) YLDs; (**F**) ASYR. SDI, socio-demographic index; ASIR, age-standardized incidence rate; ASPR, age-standardized prevalence rate; YLDs, years lived with disability; ASYR, age-standardized YLDs rate

Globally, a total of 3,212,307 patients sufferd from lung cancer in 2019, a 1.32-fold increase from 1990 (1,385,579), while the ASPR increased marginally from 1990 to 2019 (28.39/100,000 persons vs. 38.84/100,000 persons, EAPC = 0.51) (Table 1 and Fig. 1).

**Table 1:** The spatiotemporal dynamics of lung cancer globally and regionally from 1990 to 2019

Characteristics	Prevalence			Incidence			YLDs		
	2019 ASR /100 000 (95% UI)	1990–2019 EAPC in ASR (95% CI)	1990–2019 Change in Counts	2019 ASR /100 000 (95% UI)	1990–2019 EAPC in ASR (95% CI)	1990–2019 Change in Counts	2019 ASR /100 000 (95% UI)	1990–2019 EAPC in ASR (95% CI)	1990–2019 Change in Counts
Global	38.84(35.49–42.16)	0.51(-0.98,2.02)	1.32	27.66(25.28–29.99)	-0.09(-1.88,1.73)	1.01	6.62(4.82–8.52)	0.03(-3.62,3.83)	1.07
Sex									
Female	25.22(22.65–27.78)	0.45(-1.13,2.05)	1.94	16.84(15.03–18.59)	0.7(-1.75,3.2)	1.52	4.09(2.94–5.29)	0.8(-4.14,5.99)	1.58
Male	54.52(49.05–60.18)	-0.19(-2.5,2.17)	1.09	40.44(36.55–44.42)	-0.46(-1.9,1)	0.83	9.58(6.98–12.26)	-0.33(-3.29,2.73)	0.89
SDI Regions									
Low SDI	8.47(7.27–9.92)	0.13(-2.18,2.49)	1.32	8.07(6.96–9.49)	-0.16(-3.43,3.21)	1.08	2.01(1.41–2.69)	0.15(-6.45,7.21)	1.28
Low-middle SDI	12.88(11.56–14.11)	0.35(-1.71,2.45)	1.61	12.58(11.33–13.76)	0.39(-2.36,3.21)	1.54	2.88(2.06–3.73)	0.42(-5.25,6.43)	1.54
Middle SDI	32.03(27.87–36.55)	0.42(-1.74,2.62)	2.52	23.69(20.85–26.46)	0.44(-1.58,2.5)	1.68	6.23(4.45–8.11)	0.97(-3.11,5.22)	2.1
High-middle SDI	44.49(39.76–49.16)	-0.49(-3.23,2.33)	1.15	32.6(29.29–35.7)	-0.15(-1.79,1.51)	0.8	7.8(5.62–10.07)	0(-3.36,3.48)	0.87
High SDI	68.54(62.03–74.87)	0.45(-1.48,2.42)	0.96	37.36(33.86–40.77)	-0.48(-1.97,1.04)	0.59	9.57(6.91–12.22)	-0.25(-3.23,2.82)	0.67
GBD Regions									
High-income Asia Pacific	74.32(63.96–85.75)	-0.37(-2.92,2.25)	2.23	31.57(26.96–36.26)	0.11(-1.59,1.84)	1.47	9.06(6.47–11.91)	0.56(-2.72,3.94)	1.67
Central Asia	20.13(18.23–22.19)	-0.51(-2.28,1.29)	-0.01	18.91(17.14–20.76)	-1.52(-3.46,0.46)	-0.01	4.33(3.09–5.67)	-1.56(-5.56,2.61)	-0.01
East Asia	54.46(45.82–63.97)	0.32(-1.07,1.74)	3.03	41.31(35.03–48.11)	1.11(-0.52,2.76)	2.22	9.74(6.84–12.83)	1.26(-2.11,4.74)	2.33
Southeast Asia	22.31(18.65–25.86)	1.28(-0.83,3.43)	1.58	21.99(18.43–25.41)	0.24(-1.82,2.34)	1.51	4.91(3.37–6.56)	0.24(-4.07,4.74)	1.51
South Asia	8.31(7.1–9.5)	0.57(-2.19,3.4)	1.8	8.36(7.14–9.52)	0.33(-3.01,3.78)	1.79	1.92(1.32–2.56)	0.35(-6.51,7.72)	1.77
Australasia	58.5(47.1–72.22)	-0.42(-1.44,0.62)	1.22	30.69(24.98–37.48)	-0.73(-2.35,0.91)	0.7	8(5.51–11)	-0.44(-3.65,2.88)	0.83
Oceania	20.58(15.67–28.96)	1.45(-0.46,3.39)	1.65	21.63(16.64–30.37)	0.37(-1.73,2.52)	1.6	4.75(2.98–7.13)	0.34(-4.07,4.96)	1.59
High-income North America	80.37(70.29–92.13)	-0.38(-2.98,2.29)	0.63	44.96(39.48–51.36)	-0.89(-2.21,0.45)	0.41	11.11(8–14.39)	-0.8(-3.46,1.93)	0.45
Caribbean	25.86(22–30.46)	-0.84(-2.38,0.71)	1.25	22.19(18.95–25.96)	0.04(-1.97,2.1)	1.03	5.11(3.59–6.88)	0.1(-4.07,4.45)	1.05
Andean Latin America	10.96(8.76–13.46)	-1.63(-3.5,0.27)	1.3	10.8(8.7–13.21)	-0.62(-3.35,2.19)	1.27	2.47(1.68–3.47)	-0.61(-6.23,5.35)	1.26
Central Latin America	12.87(10.85–15.02)	0.35(-0.84,1.54)	1.48	11.69(9.97–13.63)	-0.71(-3.32,1.97)	1.32	2.7(1.86–3.67)	-0.61(-6,5.09)	1.35
Southern Latin America	26.46(20.82–33.35)	1.97(0.43,3.54)	0.51	23.63(18.8–29.51)	-0.73(-2.57,1.15)	0.44	5.43(3.54–7.78)	-0.7(-4.51,3.25)	0.44
Central Europe	48.84(42.6–55.67)	0.06(-1.24,1.36)	0.48	40.04(35.21–45.2)	0.04(-1.46,1.57)	0.41	9.24(6.47–12.12)	0.09(-3.03,3.31)	0.41
Eastern Europe	33.53(29.76–37.86)	-0.31(-2.46,1.89)	-0.08	25.04(22.22–28.14)	-1.23(-2.96,0.52)	-0.17	6.01(4.25–7.84)	-1.1(-4.62,2.55)	-0.14
Western Europe	57.24(49.68–65.59)	0.17(-3.52,4.01)	0.75	34.54(30.13–39.38)	-0.31(-1.89,1.28)	0.4	8.71(6.17–11.31)	-0.09(-3.25,3.18)	0.47
Tropical Latin America	16.23(15.31–17.01)	0.47(-2.92,3.98)	1.43	15.41(14.47–16.17)	-0.36(-2.7,2.03)	1.38	3.51(2.51–4.5)	-0.31(-5.16,4.8)	1.39
North Africa and Middle East	17.17(15.15–19.45)	1.4(0.15,2.66)	1.54	16.83(14.91–19.02)	0.02(-2.28,2.39)	1.47	3.8(2.72–5.04)	0.01(-4.78,5.05)	1.47
Central Sub-Saharan Africa	12.41(8.06–21.06)	0.26(-1.01,1.55)	1.09	12.74(8.4–21.23)	-0.46(-3,2.16)	1.04	2.87(1.63–5.2)	-0.44(-5.74,5.15)	1.05
Eastern Sub-Saharan Africa	6.63(5.56–8.03)	0.57(-0.74,1.91)	1.26	7.03(5.95–8.47)	0.1(-3.47,3.81)	1.19	1.62(1.08–2.28)	0.1(-7.2,7.98)	1.21
Southern Sub-Saharan Africa	18.33(16.61–20.46)	0.22(-3.07,3.62)	0.85	18.37(16.72–20.33)	-0.27(-2.43,1.93)	0.86	4.08(2.87–5.4)	-0.31(-4.82,4.42)	0.84
Western Sub-Saharan Africa	8.52(7.13–9.94)	0.56(-2.82,4.05)	1.47	9.17(7.74–10.64)	0.59(-2.68,3.96)	1.44	2.07(1.43–2.75)	0.54(-6.18,7.73)	1.43

Abbreviations: *SDI* socio-demographic index, *GBD* global burden of disease, *ASR* age standardized rate, *UI* uncertainty interval, *EAPC* estimated annual percentage change, *CI* confidence interval, *YLDs* years lived with disability

**Fig. 2:**
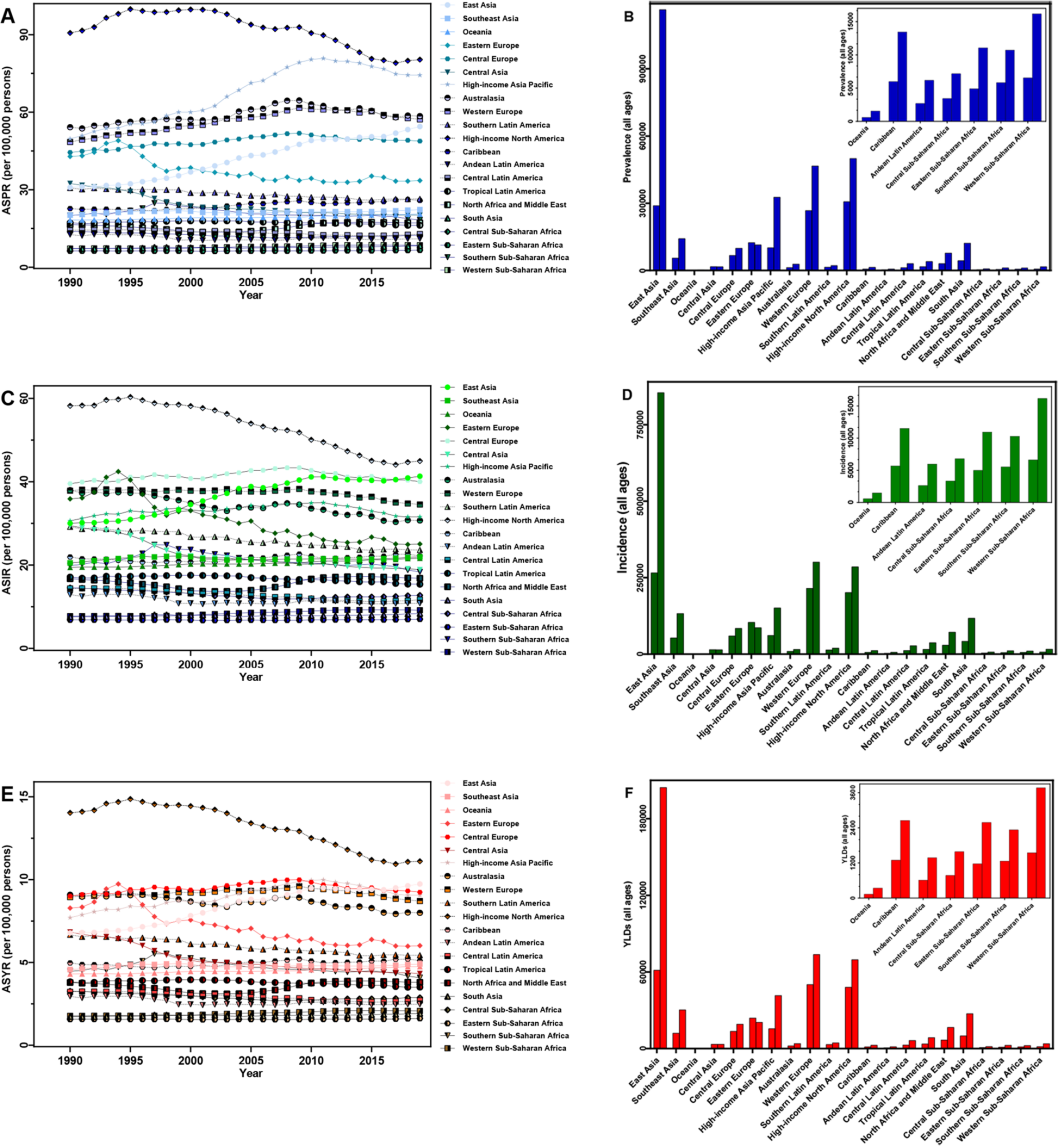
The epidemiology of lung cancer in 21 GBD regions from 1990 to 2019. (**A**) prevalent cases; (**B**) ASPR; (**C**) incident cases; (**D**) ASIR; (**E**) YLDs; (**F**) ASYR. Those data from certain regions can be viewed in the top-right of the panel. SDI, socio-demographic index; ASIR, age-standardized incidence rate; ASPR, age-standardized prevalence rate; YLDs, years lived with disability; ASYR, age-standardized YLDs rate

In 2019, low SDI regions had the lowest absolute number (45,593) and ASR (8.47/100,000 persons) of lung cancer prevalence, with the highest absolute number (1,250,089) and ASR (68.54/100,000 persons) in high SDI regions. Furthermore, the number of lung cancer patients increased in all SDI regions, with the largest increase in middle SDI regions (2.52-fold), while the ASPR only decreased in high-middle SDI regions (EAPC = -0.49) from 1990 to 2019 (Table 1 and Fig. 1). Among 21 GBD regions, East Asia (1,163,481), high-income North America (499,571) and Western Europe (466,299) exhibited the highest prevalent cases in 2019. Meanwhile, the highest ASPR was observed in high-income North America (80.37/100,000 persons), followed by high-income Asia Pacific (74.32/100,000 persons), Australasia (58.50/100,000 persons) and Western Europe (57.24/100,000 persons). Over the past 30 years, the most significant increase in lung cancer patients (3.03-fold) was observed in East Asia, with the highest EAPC of ASPR (1.97) in Southern Latin America (Table 1 and Fig. 2).

**Fig. 3:**
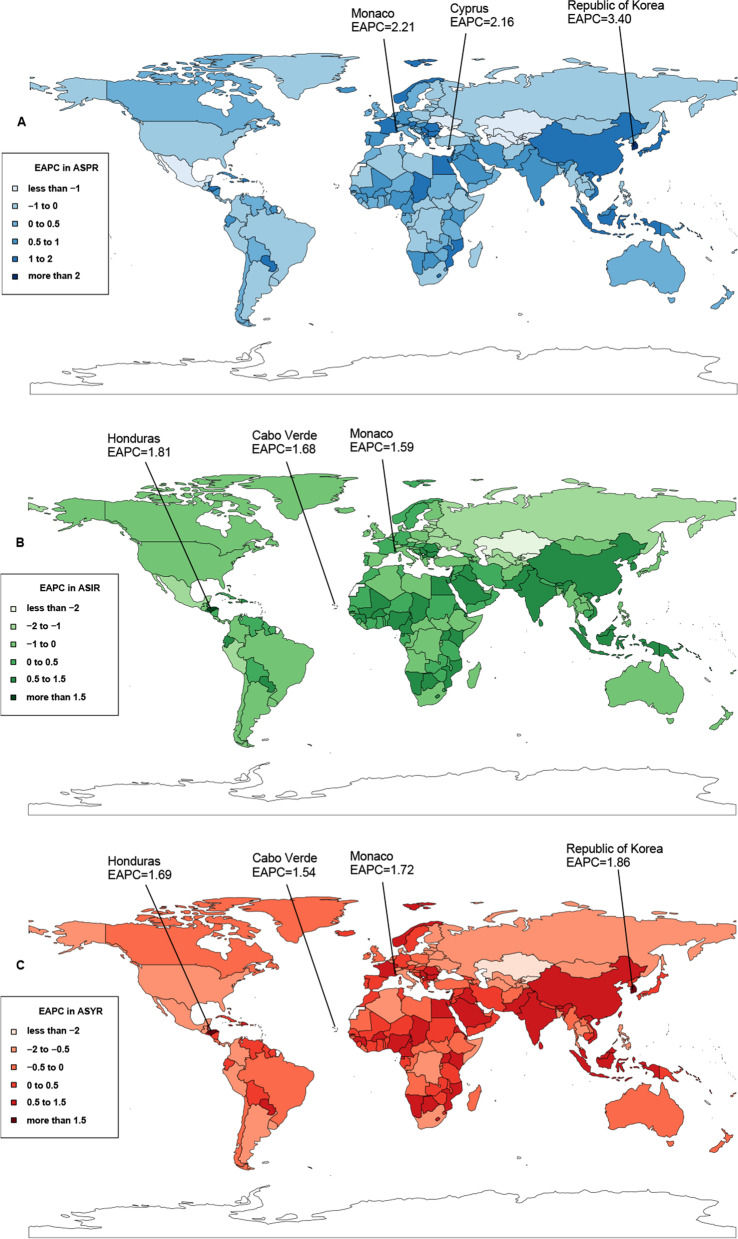
The trends of lung cancer for both sexes in 204 countries and territories from 1990 to 2019. (**A**) The EAPC in ASPR; (**B**) The EAPC in ASIR; (**C**) The EAPC in ASYR. EAPC, estimated annual percentage change; ASPR, age-standardized prevalence rate; ASIR, age-standardized incidence rate; ASYR, age-standardized YLDs rate

Among 204 countries and territories, Monaco (119.00/100,000 persons), Greenland (82.90/100,000 persons), and Canada (81.97/100,000 persons) had the largest ASPR (Additional file 1: Table S1, Additional file 2: Fig. S1A), with the greatest prevalent cases in China (1,137,880), United States of America (444,083), and Japan (253,321) in 2019 (Additional file 1: Table S1, Additional file 2: Fig. S1B). During the study, the temporal trends of lung cancer ASPR exhibited significant heterogeneity worldwide, with the largest increase in South Korea (EAPC = 3.40) and the greatest decrease in Kyrgyzstan (EAPC = -2.76) (Additional file 1: Table S1, and Fig. 3). Lung cancer patients increased in approximately 95.59% of all countries, and the most remarkable increase was noticed in the United Arab Emirates (8.43-fold), Qatar (7.37-fold) and Republic of Korea (6.29-fold) (Additional file 1: Table S1, Additional file 2: Fig. S1C).

### Lung cancer incidence

In 2019, there were 2,259,998 newly diagnosed lung cancer patients with an ASIR of 27.66/100,000 persons worldwide (Table 1 and Fig. 1). Over the past 30 years, lung cancer cases increased in almost all 21 GBD regions, with the highest increase recorded in East Asia (2.22-fold), followed by South Asia (1.79-fold) and Oceania (1.60-fold), and only decreased in Eastern Europe (-0.17-fold) and Central Asia (-0.01-fold). High-income North America (44.96/100,000 persons) and Eastern Sub-Saharan Africa (7.03/100,000 persons) showed the maximum and minimum value of ASIR in 2019, respectively. In addition, the most significant decrease in ASIR was observed in Central Asia (EAPC = -1.52), followed by Eastern Europe (EAPC = -1.23) and high-income North America (EAPC = -0.89) (Table 1 and Fig. 2). The absolute number of lung cancer cases increased across the five SDI quintiles from 1990 to 2019. However, the ASIR exhibited different trends in all SDI regions over time, decreasing in low, high-middle and high SDI regions, and increasing in low-middle and middle SDI regions. Furthermore, the high SDI regions had the largest cases (709,218) and ASIR (37.36/100,000 persons) in 2019. In contrast, the low SDI regions exhibited the lowest cases (40,765) and ASIR (8.07/100,000 persons) (Table 1 and Fig. 1).

The incidence of lung cancer was heterogeneous across countries. In 2019, almost 36.85% of newly diagnosed lung cancer cases were detected in China (832,922), followed by the United States (254,808) and Japan (116,798) (Additional file 1: Table S2, Additional file 2: Fig. S2A). From 1990 to 2019, the most significant rise and decline were observed in the United Arab Emirates (7.73-fold) and Ukraine (-0.35-fold), respectively (Additional file 1: Table S2, Additional file 2: Fig. S2B). Moreover, the largest ASIR was recorded in Greenland (77.71 per 100,000), followed by Monaco (75.57 per 100,000) and Montenegro (56.72 per 100,000) (Additional file 1: Table S2, Additional file 2: Fig. S2C). In addition, Honduras (EAPC = 1.81) and Kyrgyzstan (EAPC = -2.68) showed the largest increase and decrease in the ASIR during the same period, respectively (Additional file 1: Table S2, and Fig. 3).

### Lung cancer YLDs

The largest YLDs attributable to lung cancer in 2019 were observed in China (199,352), followed by the United States (61,843) and Japan (32,090) (Additional file 1: Table S3, Additional file 2: Fig. S3A). The United Arab Emirates showed the most significant increase from 1990 to 2019 (7.99-fold) (Additional file 1: Table S3, Additional file 2: Fig. S3B). In 2019, the ASYR varied considerably around the globe, from 1.29/100,000 persons in Ethiopia to 18.75/100,000 persons in Monaco (Additional file 1: Table S3, Additional file 2: Fig. S3C). Throughout the study period, the ASYR decreased in approximately half of all countries, with the largest decrease noticed in Kyrgyzstan (EAPC = -2.67), followed by Bahrain (EAPC = -2.48) and Kazakhstan (EAPC = -2.37) (Additional file 1: Table S3, and Fig. 3).

Among the five SDI regions, the YLDs ranged widely in 2019, from 10,413 in low SDI regions to 177,980 in high SDI regions, while middle SDI regions exhibited the most significant change (2.1-fold) over time. However, the ASYR decreased in high SDI regions (EAPC = -0.25) from 1990 to 2019 (Table 1 and Fig. 1). Across the 21 GBD regions, the decrease in lung cancer YLDs was noticed in Eastern Europe (-0.14-fold) and Central Asia (-0.01-fold), with the highest increase in East Asia (2.33-fold). Besides, the largest increase in lung cancer ASYR was observed in East Asia (EAPC = 1.26), with the greatest decrease in Central Asia (EAPC = -1.56) and Eastern Europe (EAPC = -1.10) (Table 1 and Fig. 2).

Globally, YLDs associated with lung cancer increased 1.07-fold from 262,763 in 1990 to 544,215 in 2019. Meanwhile, the ASYR increased from 6.56/100,000 persons in 1990 to 6.62/100,000 persons in 2019 (EAPC = 0.03) (Table 1 and Fig. 1).

### Age-sex patterns of prevalence

**Fig. 4:**
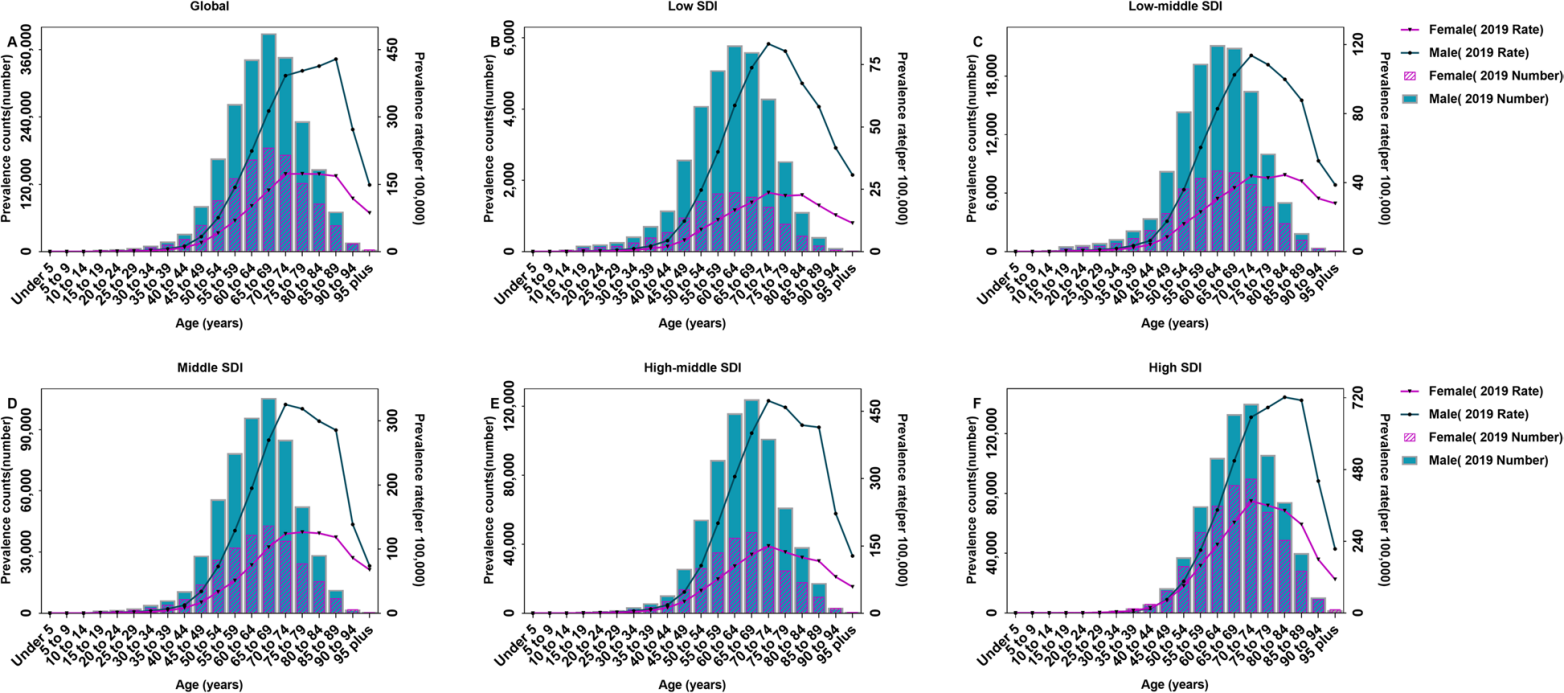
The absolute number of prevalent cases and prevalence rates/100,000 persons of lung cancer by age and sex in 2019 at (**A**) the globe; (**B**) low SDI regions; (**C**) low-middle SDI regions; (**D**) middle SDI regions; (**E**) high-middle SDI regions; and (**F**) high SDI regions. SDI: socio-demographic index

In 2019, lung cancer predominantly affected males, and the prevalence counts increased with age, peaking in the 65–69 age group for both genders worldwide. Meanwhile, the prevalence rate peaked in the 85–89 age group for males and the 75–79 age group for females. Similar to the global lung cancer prevalence, significantly fewer female patients were affected than males in all SDI quintiles, although age-sex patterns of prevalent estimates differed significantly. In high SDI regions, the prevalence rate peaked in the 80–84 age group for males and 70–74 age group for females, and the prevalence counts peaked in the 70–74 age group for both genders. In low SDI regions, the prevalence counts and rates peaked in the 60–64 and 70–74 age groups for both genders, respectively (Fig. 4).

### Decomposition analysis for incidence

**Fig. 5:**
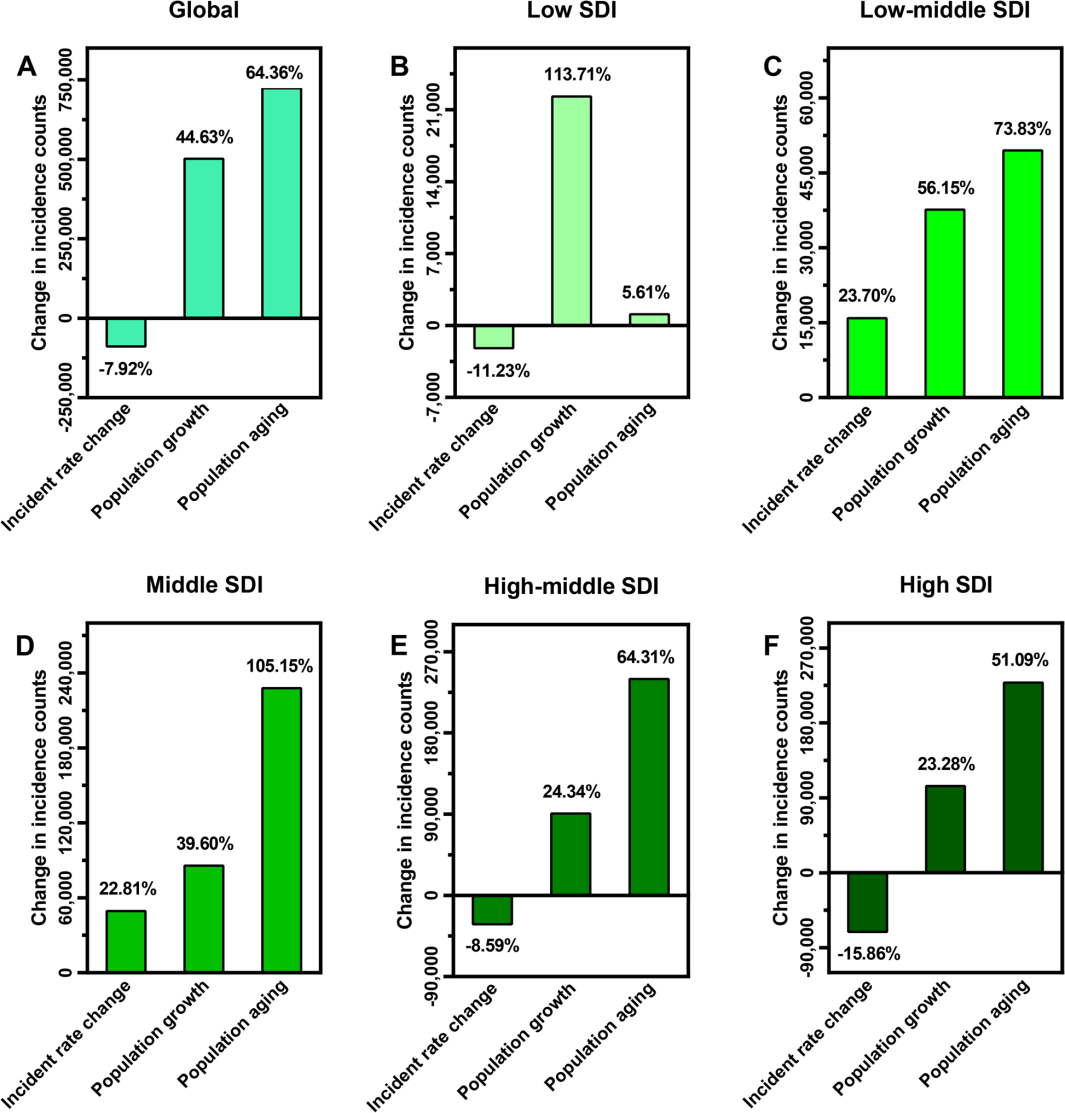
The proportions of lung cancer incident cases varied from 1990 to 2019 attributed to incident rate change, population growth, and population ageing at (**A**) the globe; (**B**) low SDI regions; (**C**) low-middle SDI regions; (**D**) middle SDI regions; (**E**) high-middle SDI regions; and (**F**) high SDI regions. SDI: socio-demographic index

A 101.07% increase was observed in the global incidence counts of lung cancer over the past three decades, of which 44.63% was attributed to population growth and 64.36% to population aging, despite a -7.92% decrease in per capita burden of lung cancer. Among the five SDI regions, the increment in incident cases was tightly correlated to population growth in low SDI regions, and population aging played a prominent role in the other SDI regions, while high SDI regions showed the largest decrease in per capita burden of lung cancer (Fig. 5).

### Correlation evaluation about YLDs

**Fig. 6:**
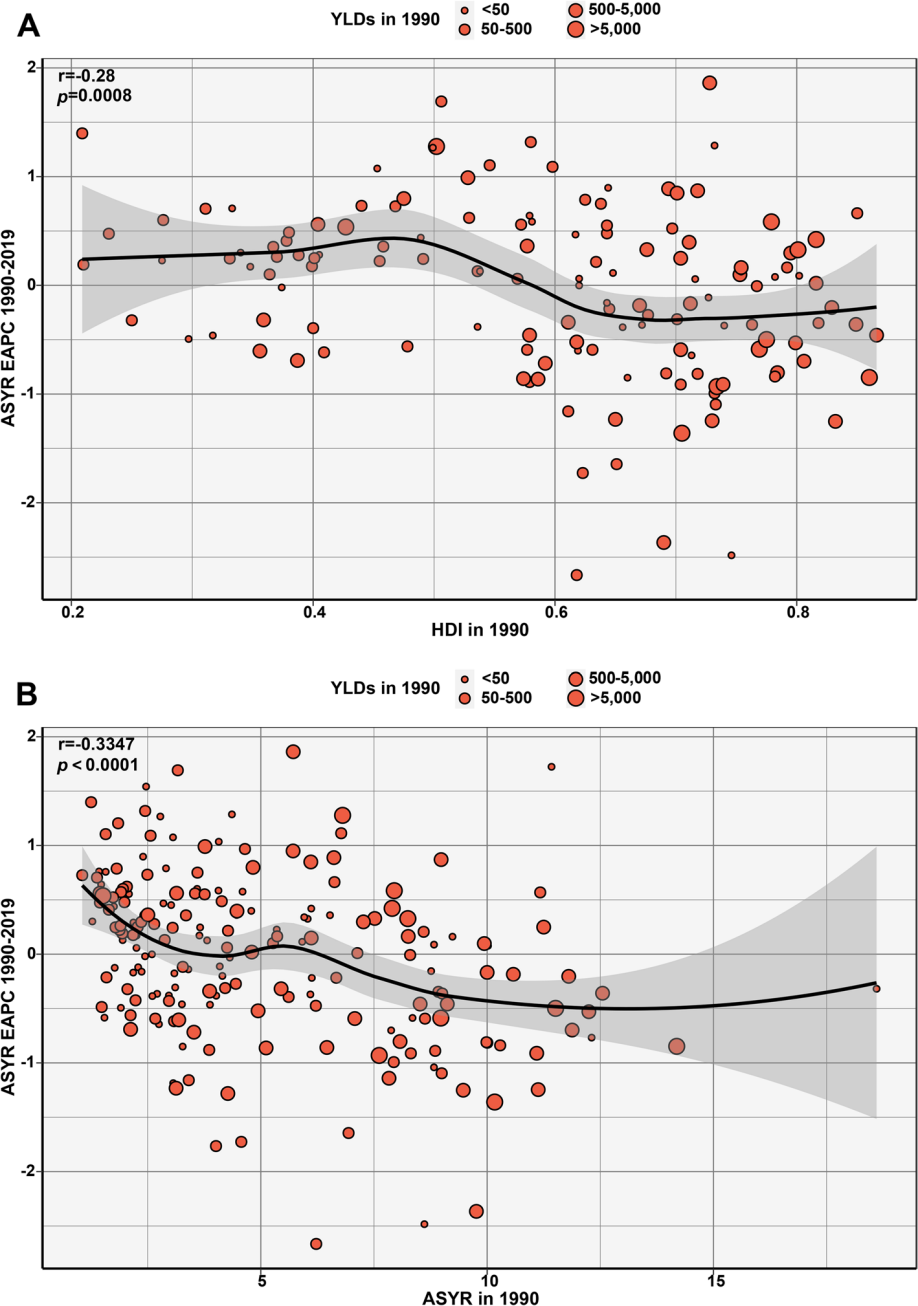
The correlation of lung cancer between EAPC of ASYR and **A** HDI in 1990, and **B** ASYR in 1990. The circles represent countries that were available on HDI data and GHDx query tool. The size of circle is increased with the absolute number of lung cancer. The r indices and *p* values presented in (**A**) and (**B**) were derived from Pearson correlation analysis. EAPC, estimated annual percentage change; ASYR, age-standardized YLDs rate; HDI, human development index; GHDx, global health data exchange

As shown in Fig. 6A and B, the EAPC of ASYR was correlated with HDI (1990) and ASYR (1990). The HDI in 1990 could reflect the level and effectiveness of the health care system for every country, and the ASYR attributable to lung cancer in 1990 could reflect the disease reservoir at baseline. As expected, countries with lower HDI in 1990 experienced a more rapid increase in ASYR attributable to lung cancer from 1990 to 2019 (*r* = -0.28, *p* = 0.0008). Furthermore, a significant negative correlation was found between EAPC and ASYR in 1990 (*r* = -0.3347, *p* < 0.0001).

## Discussion

The present study sought to assess the spatiotemporal dynamics of lung cancer during the past 30 years. We found that prevalence, incidence and YLDs varied significantly across 204 countries and territories. According to age-sex patterns of prevalence, male patients suffered more from lung cancer than females. Through the decomposition analysis, it was found that population aging contributed mainly to the lung cancer incidence. Furthermore, the EAPC of ASYR was significantly correlated with ASYR and HDI in 1990 across nation-level units.

Globally, lung cancer prevalence and YLDs have increased significantly over the past 30 years from 1990 to 2019. In addition, there was no decreasing trend globally, as well as in the vast majority of countries and regions, even after age standardization, which may be attributed to the following reasons.

First, tobacco use is well-established as one of the biggest threats to public health and an important risk factor for lung cancer [13], with high consumption worldwide, especially in developing countries, where it has a significant impact [14]. It is widely acknowledged that over one-third of the world's smokers live in China, which is home to more than one-fifth of the world's population [15], this could explain why China had the largest number of lung cancer patients in our study. Moreover, environmental deterioration and ecological destruction have become a global problem given the current breathtaking pace of globalization and modernization, such as air pollution. Over the years, epidemiological studies have shown that the increased lung cancer risk could be attributed to air pollution, especially exposure to particulate matter [16, 17]. Besides, the risk of indoor and occupational exposure is reportedly elevated in the context of industrialization and urbanization. As per WHO estimates, more than 107,000 people die each year from lung cancer, mesothelioma and asbestosis resulting from exposure to asbestos in the workplace [18]. Furthermore, mortality rates have improved due to the unprecedented medical progress and scientific development achieved in recent years, such as advancements in surgical techniques and nonsurgical treatments, which mainly include chemotherapy, radiation therapy, immune checkpoint blockade, and oncogene-targeted therapy [19].

Analysis of the age–sex patterns showed that lung cancer was more common in males than females at global and regional levels. Smoking, occupational risks, particulate matter pollution, and exposure to carcinogens have been reported to account for the difference in prevalence between sexes [20–22]. In this regard, it has been reported that on average, 14.4% and 11.7% of men and women are daily tobacco users, respectively, according to the Estimated Lung Cancer Statistics in the United States for 2021 [1]. Interestingly, the current evidence suggests that upregulation of glycolysis promotes the proliferation of cancerous cells [23, 24]. Men have higher blood sugar levels than women, resulting in the disproportionate cancer risk between sexes [25]. In addition, it has been documented that immune-mediated recognition and clearance of infectious mediators associated with cancer vary between men and women, which may contribute to sex disparities in disease burden [26, 27]. It has been established that the immune response in men is less robust than that in women [28, 29], which may be attributed to a certain extent to the capacity of low levels of oestrogen to fuel the production of the acute inflammatory agents, such as interleukin-6 and tumor necrosis factor [30–32].

From 1990 to 2019, the global incident cases of lung cancer have escalated, with a 101.07% increase over the past three decades, mirroring the rise in prevalence. Moreover, relative to changes in the age-specific incidence rates, population growth and especially population aging, are the primary contributing factors to the growth in lung cancer incident cases, according to decomposition analysis. It likely explains that ASIR, an index after age standardization for incidence, has fallen over the past decades. Besides, the largest decline in per capita burden of lung cancer was observed in high SDI regions, where more resources are dedicated to implementing early detection and treatment, and strong preventive initiatives are adopted, including tobacco taxation and control of environmental risk factors.

Furthermore, we found that the temporal trend in ASYR—that is, the EAPC—from 1990 to 2019 was significantly negatively correlated with HDI in 1990. Possible explanations for this observation are as follows: (1) people in countries with lower HDI were more likely to be exposed to risk factors, such as smoking, ambient fine particles, radon and asbestos; (2) countries with higher baseline HDI provided better protective and preventive measures. As expected, a significant negative correlation was found between EAPC and ASYR in 1990. This finding may be attributed to the fact that countries with high ASYR consider lung cancer screening a top priority in their disease control guidelines due to the significant economic and social burden of this cancer at that time. Moreover, it should be borne in mind that the higher the baseline ASYR, the more difficult it is to control fluctuations in the ASYR.

Our study was based on estimates from the GBD 2019, which aims to improve analytical strategies and increase the amount of high-quality data [5, 33–35]. However, there were some limitations in our study. Indeed, the quality and quantity of estimates available from the GBD study were crucial for the accuracy of our estimates. For instance, the disease burden could not be assessed in countries and territories lacking a well-established and organized architecture to register, record and report diseases. Moreover, the GBD 2019 did not estimate the burden of small cell lung cancer and non-small cell lung cancer [36, 37], which prevented us from performing a more detailed analysis of lung cancer. Future studies should address these limitations to deepen our understanding of the overall disease burden.

## Conclusions

The ASIR of lung cancer has decreased from 1990 to 2019 globally, although a concomitant increase in ASPR and ASYR was observed. Besides, male patients were significantly more affected. The increase in incidence is mainly attributed to population aging. Additionally, the EAPC of ASYR was negatively correlated with HDI and ASYR in 1990. Differences in geographic and country-specific population characteristics emphasize the need for targeted strategies to reduce the lung cancer burden.

## Supplementary Information

**Additional file 1: TableS1.**The change of lung cancer prevalence between 1990 and 2019 at 204 countries andterritories.** Table S2.** The change oflung cancer incidence between 1990 and 2019 at 204 countries and territories.** Table S3.** The change of lung cancerYLDs between 1990 and 2019 at 204 countries and territories. YLDs, years livedwith disability.

**Additional file 2: Figure S1.** The prevalence of lungcancer for both sexes in 204 countries and territories. (A) The ASPR in 2019;(B) The prevalent cases in 2019; (C) The fold change in prevalent cases from1990 to 2019. ASPR, age-standardized prevalence rate. **Figure S2.**The incidence of lung cancer for both sexes in 204 countries and territories.(A) The incident cases in 2019; (B) The fold change in incident cases from 1990to 2019; (C) The ASIR in 2019. ASIR, age-standardized incidence rate. **Figure S3.** The YLDs of lung cancer for both sexes in 204 countries andterritories. (A) The YLDs in 2019; (B) The fold change in YLDs from 1990 to2019; (C) The ASYR in 2019. YLDs, years lived with disability; ASYR, agestandardized years lived with disability rate.

## Data Availability

We confirm that all methods were performed in accordance with the relevant guidelines and regulations. The datasets analyzed during the current study are available in http://ghdx.healthdata.org/gbd-results-tool and http://hdr.undp.org/en/data.

## Abbreviations

GBD: Global Burden of Disease Study
YLDs: Years Lived With Disability
WHO: World Health Organization
SDI: Socio-demographic Index
CODEm: Cause of Death Ensemble model
ICD: International Classification of Diseases
HDI: Human Development Index
ASR: Age-Standardized Rate
ASPR: Age-Standardized Prevalence Rate
ASIR: Age-Standardized Incidence Rate
ASYR: Age-Standardized YLDs Rate
EAPC: Estimated Annual Percentage Change
CI: Confidence Interval
