# Supplementary material for: The spatiotemporal dynamics of lung cancer: 30-year trends of epidemiology across 204 countries and territories
Source: BMC Public Health. 2022 May 16;22:987. doi: 10.1186/s12889-022-13281-y (PMC9109351; doi:10.1186/s12889-022-13281-y)
Supplement: Supplementary file 1 — Additional file 1: TableS1.The change of lung cancer prevalence between 1990 and 2019 at 204 countries andterritories. Table S2. The change oflung cancer incidence between 1990 and 2019 at 204 countries and territories. Table S3. The change of lung cancerYLDs between 1990 and 2019 at 204 countries and territories. YLDs, years livedwith disability. [file 12889_2022_13281_MOESM1_ESM.zip › Revised Table S1.docx]

| Table S1. The change of lung cancer prevalence between 1990 and 2019 at 204 countries and territories. | | | | | | | | | | |
| --- | --- | --- | --- | --- | --- | --- | --- | --- | --- | --- |
|  |  | All-age Prevalence | | |  |  | ASPR | | |  |
| Region | 1990 No. (95% UI) | | 2019 No. (95% UI) | Change in absolute number  (95% UI) | | 1990 per 100,000 No. (95% UI) | | 2019 per 100,000 No. (95% UI) | EAPC No. (95% CI) | |
| Afghanistan | 931.87(445.13-1609.47) | | 1575.6(895.1-2505.23) | 0.69(0.15-1.6) | | 12.41(6.16-21.21) | | 11.25(6.78-17.88) | -0.34(-3.07-2.47) | |
| Albania | 680.35(635.47-726.57) | | 1330.78(964.24-1798) | 0.96(0.4-1.65) | | 31.05(28.95-33.18) | | 31.26(22.77-42.06) | 0.02(-1.67-1.75) | |
| Algeria | 1406.6(1123.67-1763.23) | | 3358.76(2575.58-4327.03) | 1.39(0.68-2.33) | | 11.21(9.06-14) | | 9.75(7.51-12.52) | -0.48(-3.38-2.51) | |
| American Samoa | 6.39(5.48-7.29) | | 12.82(10.87-14.91) | 1.01(0.62-1.48) | | 26.76(23.08-30.57) | | 26.03(22.18-29.98) | -0.1(-1.94-1.78) | |
| Andorra | 32.08(24.68-43.81) | | 86.58(65.51-110.3) | 1.7(0.82-2.84) | | 55.46(42.68-75.4) | | 62.49(47.32-79.53) | 0.41(-0.83-1.67) | |
| Angola | 597.69(386.68-852.66) | | 1662.68(1278.49-2152.36) | 1.78(0.86-3.32) | | 13.82(9.26-19.32) | | 13.64(10.76-17.45) | -0.04(-2.59-2.57) | |
| Antigua and Barbuda | 5.12(4.69-5.59) | | 10.76(9.07-12.61) | 1.1(0.71-1.52) | | 10.07(9.24-11.03) | | 10.35(8.79-12.07) | 0.09(-2.86-3.13) | |
| Argentina | 10672.6(10314.06-11055.77) | | 15088.92(11888.74-19081.65) | 0.41(0.11-0.79) | | 32.62(31.53-33.82) | | 28.67(22.53-36.26) | -0.45(-2.15-1.29) | |
| Armenia | 1279.67(1213.55-1348.63) | | 1489.61(1239.77-1771.27) | 0.16(-0.04-0.4) | | 41.85(39.76-44.04) | | 35.13(29.38-41.81) | -0.6(-2.13-0.95) | |
| Australia | 10789.51(10322.96-11267.31) | | 23935.24(18410.86-30509.78) | 1.22(0.7-1.86) | | 55.19(52.8-57.61) | | 59.09(45.35-75.66) | 0.24(-1.02-1.51) | |
| Austria | 4407.59(4184.59-4654.22) | | 8917.52(7122.88-10868.5) | 1.02(0.62-1.48) | | 40.25(38.22-42.56) | | 56.33(45.03-69.1) | 1.17(-0.24-2.59) | |
| Azerbaijan | 1649.44(1501.44-1799.75) | | 2746.29(2010.7-3521.5) | 0.66(0.23-1.18) | | 29.23(26.73-31.81) | | 25.34(18.71-32.12) | -0.49(-2.3-1.35) | |
| Bahamas | 28.44(25.6-31.56) | | 65.73(53.15-82.61) | 1.31(0.82-1.97) | | 17.67(15.88-19.56) | | 15.92(12.92-19.88) | -0.36(-2.66-1.99) | |
| Bahrain | 58.32(49.43-67.88) | | 154.53(116.66-205.12) | 1.65(0.92-2.8) | | 36.04(30.59-41.69) | | 18.01(13.69-22.9) | -2.36(-4.25--0.44) | |
| Bangladesh | 4298.12(3091-5595.86) | | 9890.12(6398.48-15551.36) | 1.3(0.48-2.34) | | 8.89(6.41-11.56) | | 7.4(4.82-11.58) | -0.63(-3.92-2.77) | |
| Barbados | 27.16(24.96-29.34) | | 53.1(42.99-63.96) | 0.95(0.55-1.39) | | 9.95(9.14-10.8) | | 10.88(8.83-13.12) | 0.31(-2.62-3.33) | |
| Belarus | 5110.97(4902.84-5317.42) | | 4689.51(3579.91-6124.14) | -0.08(-0.3-0.2) | | 38.39(36.93-39.91) | | 29.73(22.75-39.08) | -0.88(-2.5-0.77) | |
| Belgium | 8894.41(8466.57-9360.6) | | 12260.29(9448-15644.14) | 0.38(0.05-0.76) | | 60.3(57.56-63.42) | | 59.89(45.8-76.82) | -0.02(-1.25-1.22) | |
| Belize | 9.65(8.62-10.67) | | 41.7(35.56-48.61) | 3.32(2.56-4.22) | | 10.32(9.22-11.43) | | 14.25(12.11-16.46) | 1.12(-1.64-3.95) | |
| Benin | 185.62(153.66-219.61) | | 483.47(359.19-639.95) | 1.6(0.92-2.52) | | 9.23(7.65-10.93) | | 9.8(7.48-12.84) | 0.21(-2.85-3.36) | |
| Bermuda | 26.15(24.2-28.29) | | 47.63(39.9-58.03) | 0.82(0.49-1.27) | | 41.38(38.36-44.75) | | 37.9(31.6-46.02) | -0.3(-1.81-1.22) | |
| Bhutan | 15.72(10.59-22.36) | | 43.39(31.15-59.94) | 1.76(0.91-3.06) | | 5.9(4.1-8.36) | | 7.58(5.44-10.45) | 0.87(-2.81-4.68) | |
| Bolivia (Plurinational State of) | 405.45(271.91-527.83) | | 1137.38(747.64-1569.14) | 1.81(1.05-2.81) | | 12.18(8.12-15.8) | | 12.72(8.31-17.5) | 0.15(-2.53-2.9) | |
| Bosnia and Herzegovina | 1863.8(1748.08-1975.93) | | 2753.69(2126.68-3521.2) | 0.48(0.13-0.89) | | 40.87(38.54-43.15) | | 46.12(35.61-58.73) | 0.42(-1.03-1.89) | |
| Botswana | 109.91(84.31-139.84) | | 326.39(223.63-440.89) | 1.97(1.03-3.23) | | 18(13.9-22.7) | | 22.1(15.46-29.39) | 0.71(-1.43-2.9) | |
| Brazil | 16199.17(15748.85-16591.04) | | 39012.31(36896.68-40900.84) | 1.41(1.29-1.53) | | 17.33(16.8-17.76) | | 16.24(15.31-17.02) | -0.22(-2.53-2.13) | |
| Brunei Darussalam | 45.72(37.47-53.86) | | 158.56(138.78-179.78) | 2.47(1.81-3.46) | | 47.31(38.93-55.76) | | 54.57(48.21-61.68) | 0.49(-0.85-1.85) | |
| Bulgaria | 4148.26(3889.91-4414.64) | | 5737.34(4490.47-7219.35) | 0.38(0.09-0.75) | | 32.86(30.83-34.91) | | 45.03(34.97-57.09) | 1.09(-0.46-2.67) | |
| Burkina Faso | 349.29(265.22-477.8) | | 880.16(675.52-1183.15) | 1.52(0.94-2.28) | | 7.74(5.93-10.54) | | 9.39(7.32-12.66) | 0.67(-2.58-4.03) | |
| Burundi | 185.37(137.82-242.42) | | 320.06(221.7-447.06) | 0.73(0.12-1.7) | | 7.64(5.73-10.01) | | 6.69(4.68-9.14) | -0.46(-3.96-3.17) | |
| Cabo Verde | 24.17(21.64-26.83) | | 71.48(56.88-86.44) | 1.96(1.3-2.63) | | 10.48(9.43-11.6) | | 16.92(13.33-20.51) | 1.67(-1-4.4) | |
| Cambodia | 1081.64(826.02-1387.25) | | 2961.87(2290.27-3680.8) | 1.74(0.94-2.71) | | 22.84(17.63-28.76) | | 23.96(18.86-29.67) | 0.16(-1.8-2.16) | |
| Cameroon | 485.43(377.41-620.16) | | 1586.55(1124.85-2201.85) | 2.27(1.23-3.69) | | 10.47(8.17-13.33) | | 12.91(9.24-17.74) | 0.72(-2.07-3.6) | |
| Canada | 23852.88(22542.79-25339.25) | | 55418.74(41887.88-71874.38) | 1.32(0.75-2.01) | | 74.4(70.41-78.88) | | 81.97(61.93-106.42) | 0.33(-0.75-1.43) | |
| Central African Republic | 183.16(92.42-303.87) | | 301.15(148.17-534.93) | 0.64(0.17-1.19) | | 13.85(7.42-22.56) | | 12.07(6.4-20.76) | -0.47(-3.09-2.21) | |
| Chad | 216.56(157.02-287.18) | | 617.78(438.7-881.04) | 1.85(1.09-2.78) | | 7.53(5.46-10.02) | | 10.87(7.77-15.51) | 1.28(-1.92-4.57) | |
| Chile | 1943.39(1861.45-2027.66) | | 4682.23(3631.69-5919.37) | 1.41(0.85-2.07) | | 19.03(18.24-19.86) | | 19.38(15.05-24.49) | 0.06(-2.1-2.27) | |
| China | 279310.11(239824.42-320348.04) | | 1137880.03(950548.16-1344733.04) | 3.07(2.22-4.2) | | 31.05(26.74-35.48) | | 55.13(46.21-64.95) | 2(0.46-3.56) | |
| Colombia | 2858.58(2748.04-2964.28) | | 7412.38(5748.64-9436.13) | 1.59(1-2.31) | | 15.78(15.13-16.37) | | 14.08(10.9-17.87) | -0.39(-2.83-2.11) | |
| Comoros | 16.24(8.7-21.5) | | 34.85(25.19-47.02) | 1.15(0.41-3.05) | | 7.16(3.99-9.48) | | 7.04(5.16-9.41) | -0.06(-3.58-3.59) | |
| Congo | 201.18(112.16-306.39) | | 436.51(298.37-636.97) | 1.17(0.44-2.43) | | 17.32(10.05-25.92) | | 15.49(10.98-22.01) | -0.38(-2.71-2) | |
| Cook Islands | 4.52(3.81-5.32) | | 7.61(6.47-9.05) | 0.68(0.32-1.21) | | 34.83(29.54-40.7) | | 30.57(25.89-36.21) | -0.45(-2.1-1.23) | |
| Costa Rica | 209.79(197.42-221.72) | | 596.04(459.27-761.69) | 1.84(1.18-2.64) | | 12.01(11.28-12.71) | | 11.6(8.93-14.79) | -0.12(-2.86-2.7) | |
| Croatia | 3854.38(3550.39-4194.58) | | 5337.58(4123.26-6768.04) | 0.38(0.06-0.77) | | 57.95(53.52-62.85) | | 65.31(50.42-83.33) | 0.41(-0.8-1.65) | |
| Cuba | 3612.59(3464.5-3753.5) | | 8223.13(6614.79-10108.13) | 1.28(0.83-1.78) | | 35.13(33.64-36.5) | | 43.8(35.09-53.52) | 0.76(-0.77-2.32) | |
| Cyprus | 168.55(144.79-195.04) | | 737.54(626.65-856.86) | 3.38(2.49-4.48) | | 20.34(17.54-23.47) | | 37.79(32.07-43.96) | 2.16(0.28-4.08) | |
| Czechia | 7539.79(7253.62-7881.06) | | 9367.69(7573.79-11559.44) | 0.24(0-0.54) | | 56.17(54.11-58.73) | | 45.93(36.89-56.75) | -0.69(-2.02-0.65) | |
| Côte d'Ivoire | 473.46(341.37-610.24) | | 1307.53(949.09-1715.33) | 1.76(1-2.82) | | 11.12(8.15-14.32) | | 11.99(8.9-15.3) | 0.26(-2.52-3.12) | |
| Democratic People's Republic of Korea | 4854.88(3615.09-6496.08) | | 9574.06(7289.32-12305.07) | 0.97(0.47-1.58) | | 27.41(21.03-36.06) | | 29.05(22.14-37.09) | 0.2(-1.59-2.02) | |
| Democratic Republic of the Congo | 2310.13(1255.21-5192.32) | | 4471.98(2392.78-9048.6) | 0.94(0.33-1.9) | | 13.51(7.59-29.99) | | 11.57(6.14-23.21) | -0.53(-3.19-2.2) | |
| Denmark | 4793.57(4618.01-4975.45) | | 7223.4(5576.08-9128.91) | 0.51(0.16-0.91) | | 64.71(62.33-67.2) | | 66.41(50.97-83.61) | 0.09(-1.09-1.28) | |
| Djibouti | 12.99(8.24-19.86) | | 64.67(39.98-110.54) | 3.98(2.33-6.45) | | 8.58(5.55-12.93) | | 10.31(6.6-17.08) | 0.64(-2.46-3.83) | |
| Dominica | 11.18(9.84-12.78) | | 15.58(12.55-19.06) | 0.39(0.1-0.77) | | 16.39(14.35-18.8) | | 17.41(14-21.3) | 0.21(-2.1-2.57) | |
| Dominican Republic | 432.91(380.71-491.96) | | 1504.51(1070.44-2062.79) | 2.48(1.38-3.93) | | 11.07(9.73-12.57) | | 15.91(11.4-21.77) | 1.26(-1.38-3.97) | |
| Ecuador | 458.7(434.65-484.02) | | 1506.79(1180.41-1948.08) | 2.28(1.57-3.23) | | 8.31(7.87-8.78) | | 9.94(7.82-12.79) | 0.62(-2.53-3.86) | |
| Egypt | 2173.46(1994.46-2385.12) | | 6731.47(4752.21-9204.81) | 2.1(1.11-3.27) | | 6.62(6.01-7.27) | | 9.46(6.65-12.78) | 1.24(-2.17-4.76) | |
| El Salvador | 274.44(257.82-291.67) | | 671.32(504.8-874.7) | 1.45(0.81-2.24) | | 8.93(8.39-9.51) | | 11.44(8.57-14.91) | 0.86(-2.14-3.95) | |
| Equatorial Guinea | 26.8(13.62-45.73) | | 79.84(49.59-124.7) | 1.98(0.42-4.65) | | 12.34(6.57-20.76) | | 15.8(10.22-24.28) | 0.86(-1.7-3.48) | |
| Eritrea | 70.87(49.09-97.68) | | 238.07(179.93-314.42) | 2.36(1.32-3.99) | | 6.27(4.49-8.53) | | 8.11(6.27-10.38) | 0.89(-2.67-4.58) | |
| Estonia | 883.72(849.63-921.05) | | 805.94(626.22-1015.01) | -0.09(-0.29-0.14) | | 42.86(41.25-44.62) | | 33.31(25.74-42.23) | -0.87(-2.4-0.69) | |
| Eswatini | 49.81(33.17-71.38) | | 107.28(63.96-155.17) | 1.15(0.47-2.22) | | 15.97(10.75-22.77) | | 17.39(10.59-24.68) | 0.29(-2.03-2.67) | |
| Ethiopia | 1231.44(780.68-2178.44) | | 2113.86(1476.85-2840.81) | 0.72(0.12-2.16) | | 5.9(3.75-10.36) | | 5.16(3.59-6.98) | -0.47(-4.44-3.67) | |
| Fiji | 38.28(30.54-47.45) | | 75.37(58.53-95.52) | 0.97(0.39-1.83) | | 10.02(7.97-12.39) | | 9.67(7.59-12.03) | -0.12(-3.12-2.97) | |
| Finland | 3223.43(3064.33-3396.33) | | 5056.39(3983.33-6452.46) | 0.57(0.23-1.04) | | 46.25(43.99-48.68) | | 43(33.72-55.32) | -0.25(-1.67-1.19) | |
| France | 30789.61(29522.94-32157.47) | | 67538.08(51577.24-87410.88) | 1.19(0.69-1.83) | | 40.85(39.18-42.68) | | 59.39(45-76.7) | 1.3(-0.08-2.7) | |
| Gabon | 109.25(62.63-165.51) | | 211.96(139.11-307.9) | 0.94(0.34-1.85) | | 18.39(10.75-27.64) | | 19.04(12.73-27.15) | 0.12(-2.07-2.36) | |
| Gambia | 20.45(15.31-26.25) | | 67.65(50.68-88.01) | 2.31(1.25-3.77) | | 5.66(4.29-7.19) | | 7.06(5.34-9.13) | 0.76(-3.01-4.68) | |
| Georgia | 2285.48(2072.72-2510.42) | | 1948.7(1613.94-2329.43) | -0.15(-0.31-0.04) | | 35.09(31.77-38.45) | | 34.83(28.85-41.54) | -0.03(-1.63-1.6) | |
| Germany | 56508.68(53871.73-59489.28) | | 102335.92(77683.59-133472.27) | 0.81(0.37-1.36) | | 47.51(45.27-50.01) | | 61.97(46.93-80.98) | 0.92(-0.39-2.24) | |
| Ghana | 537.47(431.03-661.47) | | 1433.95(1137.42-1806.11) | 1.67(0.92-2.61) | | 7.76(6.29-9.43) | | 8.39(6.77-10.55) | 0.27(-3.05-3.7) | |
| Greece | 7719.15(7319.24-8173.92) | | 12189.65(9411.58-15495.83) | 0.58(0.22-1) | | 51.24(48.6-54.24) | | 60.46(46.18-77.22) | 0.57(-0.71-1.87) | |
| Greenland | 32.91(29.22-37.29) | | 61.12(50.11-72.48) | 0.86(0.48-1.28) | | 87.77(79.19-98.29) | | 82.9(68.47-97.02) | -0.2(-1.22-0.84) | |
| Grenada | 10.14(9.14-11.22) | | 17.74(15.67-20.05) | 0.75(0.49-1.03) | | 15.01(13.49-16.66) | | 15.17(13.5-16.96) | 0.04(-2.39-2.53) | |
| Guam | 29.73(25.58-34.5) | | 63(52.4-75.32) | 1.12(0.68-1.67) | | 36.89(31.46-43.07) | | 32.7(27.24-38.96) | -0.41(-2.02-1.22) | |
| Guatemala | 355.21(312.87-403.72) | | 907.8(702.66-1152.42) | 1.56(0.94-2.35) | | 8.92(7.9-10.05) | | 7.87(6.11-9.97) | -0.43(-3.67-2.92) | |
| Guinea | 273.01(229.16-323.09) | | 552.88(416.1-714.5) | 1.03(0.5-1.74) | | 8(6.73-9.42) | | 9.71(7.39-12.52) | 0.67(-2.53-3.97) | |
| Guinea-Bissau | 54.76(31.45-78.02) | | 93.96(60.39-136.86) | 0.72(0.22-1.41) | | 12.88(7.56-18.24) | | 12.37(7.94-17.79) | -0.14(-2.79-2.58) | |
| Guyana | 33.3(28.65-38.18) | | 59.76(45.85-76.34) | 0.79(0.32-1.38) | | 8.33(7.17-9.5) | | 8.92(6.91-11.32) | 0.24(-2.97-3.56) | |
| Haiti | 461.76(273.45-740.46) | | 808.67(496.6-1313.59) | 0.75(0.21-1.64) | | 13.15(7.91-21.14) | | 10.98(6.78-17.91) | -0.62(-3.33-2.16) | |
| Honduras | 301.48(239.28-359.89) | | 1469.49(958.5-2122.93) | 3.87(2.31-5.97) | | 13.8(10.91-16.4) | | 23.62(15.34-33.93) | 1.87(-0.44-4.23) | |
| Hungary | 8062.59(7773.07-8355.35) | | 11679.77(9517.11-14391.77) | 0.45(0.18-0.77) | | 56.33(54.32-58.41) | | 66.56(54.22-82.49) | 0.58(-0.65-1.82) | |
| Iceland | 142.11(130.28-156.23) | | 341.41(292.47-396.88) | 1.4(1-1.85) | | 51.77(47.53-56.79) | | 65.11(55.8-75.6) | 0.79(-0.47-2.07) | |
| India | 30306.67(25733.55-35305.27) | | 90057.7(73919.11-106986.69) | 1.97(1.19-2.76) | | 6.3(5.34-7.34) | | 7.63(6.27-9.05) | 0.66(-2.93-4.39) | |
| Indonesia | 17412.02(14950.83-19777.11) | | 50233.86(36772.54-62097.63) | 1.89(1.13-2.62) | | 16.5(14.18-18.74) | | 22.23(16.26-27.3) | 1.03(-1.16-3.28) | |
| Iran (Islamic  Republic of) | 3080.1(2585.72-3687.6) | | 9365.61(8688.94-10053.26) | 2.04(1.4-2.81) | | 10.76(8.93-12.93) | | 12.53(11.58-13.47) | 0.52(-2.26-3.39) | |
| Iraq | 1228.32(971.45-1525.27) | | 4484.06(3427.98-5628.77) | 2.65(1.62-4) | | 15.37(12.18-18.98) | | 18.76(14.44-23.11) | 0.69(-1.62-3.06) | |
| Ireland | 1810.88(1719.8-1898.26) | | 3777.23(2927.94-4843.95) | 1.09(0.62-1.69) | | 44.63(42.42-46.82) | | 51.37(39.75-66) | 0.49(-0.89-1.89) | |
| Israel | 1205.92(1147.95-1266.5) | | 3480.03(2683.12-4483.52) | 1.89(1.2-2.72) | | 25.53(24.23-26.83) | | 31.44(24.12-40.59) | 0.72(-1.08-2.55) | |
| Italy | 41825.07(39994.54-43963.97) | | 63160.34(51417.18-76260.72) | 0.51(0.22-0.82) | | 48.46(46.43-50.8) | | 49.95(40.48-60.38) | 0.1(-1.25-1.48) | |
| Jamaica | 281.89(266.19-298.17) | | 646.06(504.29-808.89) | 1.29(0.77-1.89) | | 16.52(15.59-17.49) | | 21.8(17.07-27.35) | 0.96(-1.24-3.21) | |
| Japan | 90557.63(86241.99-94625.17) | | 253320.86(207167.14-301073.65) | 1.8(1.33-2.31) | | 52.66(50.1-55.02) | | 74.49(62.1-88.37) | 1.2(-0.02-2.44) | |
| Jordan | 179.43(147.02-221.08) | | 1014.69(835.62-1232.58) | 4.66(3.21-6.57) | | 12.33(10.03-15.14) | | 14.58(12.05-17.57) | 0.58(-2.02-3.24) | |
| Kazakhstan | 6399.13(6075.36-6757.37) | | 4337.4(3675.62-5018.9) | -0.32(-0.43--0.21) | | 46.69(44.3-49.25) | | 23.34(19.84-26.85) | -2.36(-4.02--0.67) | |
| Kenya | 354.5(267.65-446.23) | | 1204.08(985.79-1460.15) | 2.4(1.72-3.4) | | 4.25(3.21-5.33) | | 5.31(4.41-6.4) | 0.77(-3.57-5.3) | |
| Kiribati | 7.99(6.41-9.82) | | 14.77(10.74-19.61) | 0.85(0.25-1.8) | | 19.07(15.31-23.2) | | 18.86(13.98-24.6) | -0.04(-2.21-2.18) | |
| Kuwait | 77.71(69.37-86) | | 258.87(215.66-310.17) | 2.33(1.75-3.09) | | 12.08(10.81-13.5) | | 10.32(8.46-12.42) | -0.54(-3.35-2.35) | |
| Kyrgyzstan | 924.83(868.5-984.03) | | 636.25(550.11-726.34) | -0.31(-0.42--0.21) | | 29.08(27.34-30.9) | | 12.91(11.21-14.63) | -2.76(-4.93--0.54) | |
| Lao People's Democratic Republic | 545.25(370.62-769.51) | | 1007.06(730.37-1329.52) | 0.85(0.21-1.67) | | 24.42(17.04-34.17) | | 22(16.25-28.49) | -0.36(-2.32-1.64) | |
| Latvia | 1508.2(1442.42-1574.3) | | 1259.2(1022.81-1549.3) | -0.17(-0.33-0.02) | | 41.92(40.12-43.71) | | 34.89(28.1-43.28) | -0.63(-2.16-0.92) | |
| Lebanon | 495.8(368.88-634.83) | | 1576.93(1266.34-2051.06) | 2.18(1.23-3.87) | | 20.61(15.41-26.17) | | 30.4(24.38-39.49) | 1.35(-0.59-3.32) | |
| Lesotho | 123.78(95.18-176.95) | | 237.46(158.36-336.97) | 0.92(0.22-1.88) | | 11.97(9.29-17.06) | | 17.52(11.78-24.57) | 1.32(-1.21-3.92) | |
| Liberia | 101.74(79.3-125.33) | | 176.08(117.82-243.69) | 0.73(0.18-1.46) | | 8.93(7.02-10.99) | | 8.55(5.86-11.78) | -0.15(-3.33-3.13) | |
| Libya | 378.22(287.5-484.11) | | 975.54(724.2-1248.31) | 1.58(0.73-2.82) | | 19.61(15-25.08) | | 18.43(13.68-23.46) | -0.21(-2.38-2) | |
| Lithuania | 1816.61(1745.14-1896.57) | | 1673.09(1352.39-2042.87) | -0.08(-0.26-0.13) | | 39.92(38.38-41.68) | | 31.68(25.49-38.86) | -0.79(-2.38-0.81) | |
| Luxembourg | 276.48(259.75-295.62) | | 482.01(392.48-590.06) | 0.74(0.42-1.16) | | 52.16(48.99-55.75) | | 50.77(41.27-62.19) | -0.09(-1.42-1.25) | |
| Madagascar | 351.93(287.94-428.33) | | 710.24(512.51-946.68) | 1.02(0.4-1.79) | | 6.52(5.34-7.93) | | 6.07(4.48-8.02) | -0.25(-3.98-3.63) | |
| Malawi | 201.26(165.03-240.91) | | 415.6(321.06-524.24) | 1.06(0.56-1.64) | | 5.07(4.2-6.03) | | 5.59(4.39-6.97) | 0.33(-3.74-4.58) | |
| Malaysia | 1518.24(1333.2-1749.54) | | 5544.16(4257.62-7066.08) | 2.65(1.69-3.94) | | 16.26(14.27-18.73) | | 20.2(15.66-25.69) | 0.75(-1.49-3.05) | |
| Maldives | 11.39(7.55-15.28) | | 29.29(24.05-34.9) | 1.57(0.7-3.12) | | 12.62(8.79-16.87) | | 9.72(7.95-11.65) | -0.89(-3.71-2) | |
| Mali | 251.97(210.91-295.85) | | 600.34(450.92-795.6) | 1.38(0.73-2.26) | | 5.84(4.91-6.8) | | 6.84(5.22-8.92) | 0.55(-3.21-4.45) | |
| Malta | 126.85(117.58-137.53) | | 265.01(222.67-314.41) | 1.09(0.73-1.54) | | 29.39(27.22-31.87) | | 30.63(25.7-36.52) | 0.14(-1.59-1.91) | |
| Marshall Islands | 4.54(2.73-6.91) | | 10.59(6.32-15.94) | 1.33(0.69-2.2) | | 25.8(15.67-39.42) | | 28.72(17.81-42.33) | 0.37(-1.45-2.23) | |
| Mauritania | 104.1(81.99-127.6) | | 210.57(141.63-300.53) | 1.02(0.33-2.2) | | 10.09(7.9-12.32) | | 10.05(6.93-14.04) | -0.01(-2.98-3.04) | |
| Mauritius | 108.97(102.17-116.29) | | 221.19(179.82-269.57) | 1.03(0.62-1.51) | | 14.09(13.25-15.05) | | 12.39(10.13-15.04) | -0.44(-3.03-2.21) | |
| Mexico | 5995.76(5839.6-6104.16) | | 11875.05(10224.84-13704.76) | 0.98(0.71-1.27) | | 13.79(13.33-14.07) | | 10.12(8.73-11.67) | -1.06(-3.79-1.75) | |
| Micronesia (Federated States of) | 13.3(9.19-19.59) | | 23.5(13.6-35.83) | 0.77(0.14-1.58) | | 26.6(18.72-38.72) | | 30.68(18.38-45.54) | 0.49(-1.29-2.31) | |
| Monaco | 37.24(29.39-45.19) | | 97.2(77.61-117.77) | 1.61(0.94-2.47) | | 63.19(49.98-76.91) | | 119(94.66-147.2) | 2.21(1.14-3.29) | |
| Mongolia | 404.87(329.09-486.94) | | 691.49(531.75-911.41) | 0.71(0.25-1.38) | | 38.07(31.09-45.61) | | 29.55(23.23-38.17) | -0.87(-2.5-0.79) | |
| Montenegro | 374.85(326.43-423.49) | | 692.01(568.35-840.1) | 0.85(0.44-1.35) | | 57.57(50.2-64.83) | | 70.6(57.82-85.83) | 0.71(-0.5-1.92) | |
| Morocco | 2150.34(1634.11-2663.13) | | 5601.63(4045.6-7309) | 1.6(0.82-2.59) | | 14.73(11.23-18.12) | | 16.63(11.96-21.41) | 0.42(-1.98-2.88) | |
| Mozambique | 297.2(242.21-354.31) | | 855.82(631.05-1151.43) | 1.88(1.01-2.91) | | 4.91(4.04-5.78) | | 7.66(5.72-10.13) | 1.55(-2.35-5.59) | |
| Myanmar | 5874.07(4013.47-9130.06) | | 10552.35(7739.66-14580.53) | 0.8(0.25-1.67) | | 23.69(16.35-36.21) | | 21.88(16.11-29.98) | -0.27(-2.25-1.75) | |
| Namibia | 44.48(36.27-54.43) | | 107.2(82.19-138.9) | 1.41(0.8-2.28) | | 5.96(4.89-7.29) | | 7.49(5.84-9.57) | 0.79(-2.88-4.6) | |
| Nauru | 1.62(1.03-2.28) | | 1.8(1.07-2.48) | 0.11(-0.18-0.5) | | 38.27(25.67-53.29) | | 37.33(23.86-49.8) | -0.09(-1.63-1.48) | |
| Nepal | 681.31(461-961.94) | | 1763.67(1266.75-2294.01) | 1.59(0.83-2.66) | | 6.83(4.56-9.73) | | 7.71(5.53-9.98) | 0.42(-3.09-4.05) | |
| Netherlands | 11957.36(11380.82-12559.81) | | 23168.58(17991.96-29380.26) | 0.94(0.48-1.46) | | 62.02(59.04-65.07) | | 71.87(55.63-91.28) | 0.51(-0.66-1.69) | |
| New Zealand | 1914.47(1799.49-2028) | | 4245.71(3481.81-5042.69) | 1.22(0.82-1.69) | | 49.08(46.28-51.9) | | 55.33(45.59-65.68) | 0.41(-0.91-1.75) | |
| Nicaragua | 116.66(103.74-131.07) | | 404.05(325.5-494.3) | 2.46(1.7-3.38) | | 7.4(6.5-8.48) | | 9.14(7.4-11.06) | 0.73(-2.58-4.15) | |
| Niger | 228.63(157.11-319.45) | | 656.03(408.95-957.98) | 1.87(1.08-2.96) | | 7.86(5.37-10.89) | | 8.33(5.3-12.02) | 0.2(-3.11-3.63) | |
| Nigeria | 2591.2(1871.96-3495.56) | | 5794.47(4461.85-7516.57) | 1.24(0.55-2.2) | | 5.83(4.27-7.79) | | 6.85(5.34-8.75) | 0.56(-3.2-4.46) | |
| Niue | 0.57(0.47-0.7) | | 0.67(0.53-0.83) | 0.17(-0.12-0.55) | | 26.85(22.1-32.95) | | 30.71(24.29-38.24) | 0.46(-1.31-2.27) | |
| North Macedonia | 664.2(609.09-721.56) | | 1598.14(1208.28-2059.36) | 1.41(0.82-2.15) | | 32.81(30.12-35.61) | | 48.11(36.56-61.85) | 1.33(-0.21-2.89) | |
| Northern Mariana Islands | 10.81(8.98-13.35) | | 26.19(21.96-30.18) | 1.42(0.93-2.02) | | 55.91(48.1-65.89) | | 46.99(40.48-53.3) | -0.6(-1.92-0.74) | |
| Norway | 2357.25(2227.06-2477.49) | | 5377.33(4455.32-6381.86) | 1.28(0.9-1.69) | | 38.09(36.15-39.97) | | 58.13(48.21-68.66) | 1.47(0.05-2.91) | |
| Oman | 62.25(44.81-81.23) | | 168.86(132.47-223.9) | 1.71(0.85-3.05) | | 8.89(6.48-11.48) | | 9.74(7.92-12.01) | 0.32(-2.78-3.51) | |
| Pakistan | 7976.78(6719.07-9263.04) | | 19631.92(14826.19-26083.02) | 1.46(0.77-2.5) | | 13.64(11.45-15.89) | | 16.44(12.48-21.7) | 0.65(-1.81-3.17) | |
| Palau | 4.26(3.35-5.46) | | 10.31(8.16-13.05) | 1.42(0.74-2.24) | | 41.49(32.88-52.95) | | 45.43(36.2-56.64) | 0.31(-1.13-1.78) | |
| Palestine | 175.52(130.2-232.71) | | 568.27(481.15-667.11) | 2.24(1.25-3.54) | | 19.45(14.51-25.84) | | 22.43(18.96-26.21) | 0.49(-1.59-2.62) | |
| Panama | 225.32(211.04-239.28) | | 510.75(391.12-654.21) | 1.27(0.73-1.92) | | 14.93(13.95-15.87) | | 12.37(9.5-15.85) | -0.65(-3.2-1.97) | |
| Papua New Guinea | 347.45(235.36-535.82) | | 1035.89(714.38-1580.36) | 1.98(1.18-3.26) | | 17.32(11.83-27.04) | | 20.39(14.46-30.87) | 0.56(-1.63-2.81) | |
| Paraguay | 239.75(212.12-273.61) | | 908.77(681.69-1174) | 2.79(1.76-4.07) | | 10.56(9.34-12.06) | | 16.19(12.19-20.83) | 1.48(-1.19-4.23) | |
| Peru | 1823.41(1557.28-2129.04) | | 3526.94(2639.54-4681.07) | 0.93(0.39-1.73) | | 14.7(12.58-17.17) | | 10.99(8.23-14.58) | -1(-3.63-1.71) | |
| Philippines | 7213.35(6339.29-8207.61) | | 14616.29(11580.69-18202.2) | 1.03(0.54-1.73) | | 22.75(20.04-25.73) | | 17.65(14.07-21.88) | -0.87(-2.97-1.28) | |
| Poland | 20546.36(20209.86-20882.61) | | 30292.81(25281.18-36146.49) | 0.47(0.23-0.76) | | 46.56(45.79-47.32) | | 44.54(37.08-53.18) | -0.15(-1.56-1.27) | |
| Portugal | 2806.82(2706.3-2908.71) | | 5227.42(4020.78-6686.08) | 0.86(0.42-1.39) | | 20.48(19.77-21.22) | | 25.58(19.5-32.89) | 0.77(-1.23-2.81) | |
| Puerto Rico | 649.35(617.28-682.6) | | 1083.71(838.9-1385.69) | 0.67(0.3-1.16) | | 17.92(17.06-18.83) | | 16.26(12.54-20.94) | -0.33(-2.61-2) | |
| Qatar | 18.26(13.66-23.53) | | 152.93(107.22-212.71) | 7.37(4.13-12.71) | | 16.31(12.37-21.29) | | 17.54(13.23-22.69) | 0.25(-2.05-2.61) | |
| Republic of Korea | 9380.52(8910.86-9928.6) | | 68345.39(55468.15-83426.36) | 6.29(4.9-8.03) | | 28.66(27.24-30.46) | | 75.66(61.36-92.07) | 3.4(1.88-4.95) | |
| Republic of Moldova | 1581.88(1510.19-1652.42) | | 1218.09(1046.71-1406.65) | -0.23(-0.34--0.11) | | 33.53(32.08-35.02) | | 21.03(18.03-24.26) | -1.6(-3.43-0.27) | |
| Romania | 8881.8(8534.18-9227.34) | | 13733.6(11257.44-16702.58) | 0.55(0.27-0.88) | | 30.45(29.29-31.58) | | 41.06(33.48-49.93) | 1.04(-0.58-2.68) | |
| Russian Federation | 72385.89(69849.18-73810.59) | | 74012.48(63149.49-86679.28) | 0.02(-0.12-0.2) | | 38.26(36.87-39.04) | | 31.52(26.9-36.93) | -0.67(-2.27-0.96) | |
| Rwanda | 252.13(197.86-316.78) | | 482.16(331.52-709.7) | 0.91(0.24-2.22) | | 8.28(6.56-10.29) | | 7.76(5.36-11.3) | -0.22(-3.54-3.2) | |
| Saint Kitts and Nevis | 4.69(4.25-5.15) | | 8.91(7.21-10.79) | 0.9(0.52-1.35) | | 13.12(11.87-14.37) | | 12.53(10.29-15.02) | -0.16(-2.79-2.54) | |
| Saint Lucia | 12.04(11.1-13.06) | | 29.08(24.4-34.65) | 1.42(1-1.92) | | 13.75(12.65-14.91) | | 13.28(11.18-15.76) | -0.12(-2.68-2.51) | |
| Saint Vincent and the Grenadines | 6.81(6.21-7.47) | | 14.7(12.73-17.01) | 1.16(0.84-1.56) | | 9.49(8.65-10.41) | | 10.66(9.24-12.31) | 0.4(-2.58-3.48) | |
| Samoa | 10.13(8.15-12.21) | | 17.1(13.15-22.12) | 0.69(0.25-1.3) | | 11.13(9.03-13.35) | | 11.32(8.77-14.41) | 0.06(-2.76-2.95) | |
| San Marino | 14.8(12.82-17.08) | | 32.7(24.33-43.32) | 1.21(0.61-2.02) | | 45.53(39.46-52.36) | | 57.2(42.27-75.82) | 0.79(-0.55-2.15) | |
| Sao Tome and Principe | 8.62(6.8-10.42) | | 19.52(14.89-24.81) | 1.26(0.68-2.06) | | 12.94(10.26-15.53) | | 18.01(14.01-22.56) | 1.15(-1.31-3.67) | |
| Saudi Arabia | 434.19(319.87-565.5) | | 1848.16(1411.21-2304.75) | 3.26(1.86-5.45) | | 7.21(5.39-9.3) | | 9.01(7.13-10.91) | 0.77(-2.58-4.23) | |
| Senegal | 336.13(247.73-432.35) | | 863.12(639.4-1140.56) | 1.57(0.87-2.57) | | 10.12(7.57-13) | | 11.37(8.58-14.92) | 0.4(-2.49-3.38) | |
| Serbia | 5392.42(4515.15-5937.45) | | 9489.67(7405.09-12061) | 0.76(0.34-1.33) | | 43.62(36.72-47.74) | | 63.36(49.19-80.68) | 1.3(-0.04-2.65) | |
| Seychelles | 10.22(8.94-11.42) | | 20.28(17.68-23.58) | 0.98(0.64-1.37) | | 18.09(15.73-20.22) | | 17.92(15.66-20.75) | -0.03(-2.26-2.24) | |
| Sierra Leone | 174.13(131.69-217.67) | | 356.51(251.44-482.37) | 1.05(0.46-1.78) | | 9(6.9-11.24) | | 9.79(6.95-13.19) | 0.29(-2.79-3.47) | |
| Singapore | 1227.64(1155.17-1302.33) | | 4818.05(3740.53-6167.55) | 2.92(2.06-4.02) | | 54.94(51.6-58.3) | | 61.39(48.02-78.24) | 0.38(-0.87-1.65) | |
| Slovakia | 3911.43(3660.46-4199.85) | | 5221.87(3981.14-6781.43) | 0.34(0-0.79) | | 65.77(61.68-70.65) | | 56.7(43.32-73.57) | -0.51(-1.72-0.72) | |
| Slovenia | 1070.12(817.91-1391.09) | | 1980.98(1511.2-2609.22) | 0.85(0.23-1.66) | | 43.48(33.3-56.32) | | 51.07(38.75-67.39) | 0.56(-0.84-1.97) | |
| Solomon Islands | 44.14(23.46-74) | | 112.49(57.41-185.15) | 1.55(0.79-2.53) | | 28.59(15.97-47.1) | | 32.53(17.81-51.95) | 0.45(-1.28-2.2) | |
| Somalia | 170.26(111-265.83) | | 377.76(211.97-634.66) | 1.22(0.41-2.19) | | 6.3(4.23-9.67) | | 5.32(3.01-8.76) | -0.58(-4.46-3.45) | |
| South Africa | 4911.88(4100.13-6450.66) | | 8877.89(7899.72-10193.71) | 0.81(0.47-1.12) | | 22.4(18.6-29.78) | | 19.17(17.1-21.95) | -0.53(-2.6-1.58) | |
| South Sudan | 224.8(147.63-342.84) | | 317.32(204.57-457.62) | 0.41(-0.01-1.07) | | 9.22(6.11-14.02) | | 8.16(5.35-11.64) | -0.42(-3.6-2.87) | |
| Spain | 24723.7(23472.95-26059.74) | | 49699.67(37825.2-64079.58) | 1.01(0.52-1.63) | | 46.93(44.62-49.39) | | 59.35(44.88-77.64) | 0.81(-0.51-2.15) | |
| Sri Lanka | 902.72(790.25-1015.58) | | 2833.98(2039.4-3892.4) | 2.14(1.21-3.34) | | 7.77(6.8-8.72) | | 10.73(7.78-14.6) | 1.12(-2.05-4.39) | |
| Sudan | 712.75(406.35-1296.86) | | 1589.38(1011.05-2462.41) | 1.23(0.44-2.73) | | 7.36(4.25-13.48) | | 8.03(5.16-12.46) | 0.3(-3.1-3.82) | |
| Suriname | 35.43(31.88-38.96) | | 96.83(79.06-117.42) | 1.73(1.19-2.38) | | 13.17(11.95-14.42) | | 15.61(12.84-18.89) | 0.59(-1.93-3.16) | |
| Sweden | 3121.91(2996.96-3240.41) | | 4989.65(4223.55-5805.28) | 0.6(0.36-0.86) | | 23.18(22.26-24.11) | | 25.73(21.72-30.01) | 0.36(-1.56-2.32) | |
| Switzerland | 4983.32(4733.39-5264.64) | | 6642.49(5128.86-8518.36) | 0.33(0.03-0.73) | | 52.04(49.4-55.13) | | 42.12(32.44-54.4) | -0.73(-2.11-0.67) | |
| Syrian Arab Republic | 586.94(452.65-737.85) | | 1509.37(1100.36-2011.15) | 1.57(0.74-2.84) | | 10.05(7.71-12.64) | | 11.37(8.39-15.01) | 0.43(-2.47-3.41) | |
| Taiwan (Province of China) | 4535.43(4392.63-4689.27) | | 16026.45(12394.57-20727.91) | 2.53(1.73-3.56) | | 26.77(25.9-27.62) | | 40.73(31.55-52.71) | 1.46(-0.23-3.18) | |
| Tajikistan | 644.74(588.41-716.75) | | 665.01(531.77-838.13) | 0.03(-0.18-0.31) | | 21.2(19.4-23.48) | | 11.93(9.67-14.81) | -1.96(-4.33-0.47) | |
| Thailand | 11224.49(9955.43-12561.5) | | 24360.83(18211.19-32137.58) | 1.17(0.57-1.94) | | 29.11(25.9-32.5) | | 23.76(17.79-31.24) | -0.7(-2.54-1.18) | |
| Timor-Leste | 47.55(35.82-63.58) | | 159.62(113-208.54) | 2.36(1.26-3.78) | | 15.62(11.97-20.65) | | 18.82(13.4-24.66) | 0.64(-1.66-3) | |
| Togo | 124(100.14-154.7) | | 404.22(291-541.69) | 2.26(1.33-3.51) | | 9.64(7.78-12.03) | | 10.6(7.84-14.06) | 0.33(-2.64-3.39) | |
| Tokelau | 0.28(0.22-0.35) | | 0.32(0.25-0.42) | 0.16(-0.15-0.56) | | 20.42(15.98-26.03) | | 23.86(18.49-30.98) | 0.54(-1.49-2.61) | |
| Tonga | 14.31(11.26-17.7) | | 21.21(17.17-25.94) | 0.48(0.09-0.99) | | 25.15(19.81-31.44) | | 26.8(21.71-32.72) | 0.22(-1.64-2.12) | |
| Trinidad and Tobago | 103.31(96.9-109.98) | | 214.03(160.12-281.65) | 1.07(0.54-1.75) | | 12(11.23-12.79) | | 11.41(8.53-15.01) | -0.17(-2.93-2.66) | |
| Tunisia | 971.26(777.04-1172.01) | | 2591.78(1812.43-3593.44) | 1.67(0.77-2.97) | | 18.54(14.88-22.33) | | 19.87(14.07-27.4) | 0.24(-1.92-2.45) | |
| Turkey | 14712.4(11389.47-18406.06) | | 31739.56(25030.55-39550.04) | 1.16(0.52-2.05) | | 38.27(29.91-47.54) | | 34.95(27.58-43.51) | -0.31(-1.88-1.28) | |
| Turkmenistan | 407.1(386.65-427.25) | | 478.24(376.27-606.32) | 0.17(-0.08-0.49) | | 18.68(17.76-19.6) | | 10.82(8.57-13.61) | -1.87(-4.37-0.7) | |
| Tuvalu | 1.68(1.27-2.47) | | 2.64(1.94-3.64) | 0.57(0.14-1.2) | | 22.98(17.61-33.49) | | 25.04(18.42-34.17) | 0.3(-1.64-2.27) | |
| Uganda | 369.86(296.94-446.5) | | 1035.09(817.33-1262.8) | 1.8(1.11-2.73) | | 5.54(4.5-6.63) | | 7.02(5.61-8.41) | 0.82(-2.98-4.77) | |
| Ukraine | 40786.52(38779.85-42943.43) | | 30169.6(24474.94-36910.48) | -0.26(-0.41--0.09) | | 56.47(53.75-59.43) | | 41.65(33.82-50.95) | -1.04(-2.4-0.33) | |
| United Arab Emirates | 66.55(49.88-86.76) | | 627.4(453.55-834.77) | 8.43(5.05-13.69) | | 17.08(12.77-21.97) | | 16.87(12.24-23.29) | -0.04(-2.33-2.3) | |
| United Kingdom | 54867.34(53284.46-56013.28) | | 82805.64(69271.25-97595.35) | 0.51(0.26-0.78) | | 61.95(60.27-63.18) | | 67.09(55.84-79.44) | 0.28(-0.91-1.48) | |
| United Republic of Tanzania | 878.94(641.77-1192.4) | | 2153.54(1527.09-3124.87) | 1.45(0.85-2.13) | | 7.73(5.69-10.41) | | 8.59(6.18-12.39) | 0.36(-2.94-3.78) | |
| United States of America | 282848.31(275206.21-289445.35) | | 444083.03(384698.74-516048.29) | 0.57(0.36-0.83) | | 92.42(90.07-94.46) | | 80.19(69.4-93.32) | -0.49(-1.51-0.54) | |
| United States Virgin Islands | 16.26(13.47-19.54) | | 49.09(40.36-57.27) | 2.02(1.38-2.88) | | 18.18(15.2-21.72) | | 26.55(21.73-31.18) | 1.31(-0.75-3.42) | |
| Uruguay | 1773.82(1688.96-1863.25) | | 1936.94(1500.25-2434.61) | 0.09(-0.17-0.4) | | 46.97(44.8-49.41) | | 39.46(30.39-49.94) | -0.6(-2.04-0.86) | |
| Uzbekistan | 2461.97(2350.17-2575.19) | | 3246.61(2682.2-3914.21) | 0.32(0.09-0.59) | | 19.9(19.02-20.79) | | 13.26(11.04-15.75) | -1.39(-3.73-1) | |
| Vanuatu | 13.35(8.4-21.64) | | 42.01(27.72-62.97) | 2.15(1.19-4.12) | | 19.49(12.32-31.01) | | 23.36(15.51-34.99) | 0.63(-1.44-2.73) | |
| Venezuela (Bolivarian Republic of) | 1964.38(1879.91-2047.3) | | 6673.45(4864.1-8705.77) | 2.4(1.49-3.45) | | 19.4(18.55-20.23) | | 22.34(16.41-29.01) | 0.49(-1.6-2.62) | |
| Viet Nam | 8942.5(6997.13-10976.49) | | 28986.91(22215.48-37389.29) | 2.24(1.32-3.48) | | 21.38(16.79-26.26) | | 28.89(22.25-36.69) | 1.04(-0.89-3.01) | |
| Yemen | 480.58(300.26-768.47) | | 1345.21(909.07-2008.32) | 1.8(0.89-3.39) | | 9.12(5.85-14.37) | | 9.57(6.56-14.22) | 0.17(-2.92-3.35) | |
| Zambia | 274.95(219.83-347.88) | | 725.38(516.42-959.22) | 1.64(0.87-2.57) | | 9.27(7.44-11.68) | | 10.31(7.43-13.4) | 0.37(-2.65-3.49) | |
| Zimbabwe | 562.51(493.21-637.3) | | 1059.16(806.46-1300.35) | 0.88(0.43-1.4) | | 13.06(11.49-14.77) | | 14.11(10.91-17.15) | 0.27(-2.3-2.9) | |
| Abbreviations: ASPR, age standardized prevalence rate; UI, uncertainty interval; EAPC, estimated annual percentage change; CI, confidence interval. | | | | | | | | | | |
