# Supplementary material for: The spatiotemporal dynamics of lung cancer: 30-year trends of epidemiology across 204 countries and territories
Source: BMC Public Health. 2022 May 16;22:987. doi: 10.1186/s12889-022-13281-y (PMC9109351; doi:10.1186/s12889-022-13281-y)
Supplement: Supplementary file 1 — Additional file 1: TableS1.The change of lung cancer prevalence between 1990 and 2019 at 204 countries andterritories. Table S2. The change oflung cancer incidence between 1990 and 2019 at 204 countries and territories. Table S3. The change of lung cancerYLDs between 1990 and 2019 at 204 countries and territories. YLDs, years livedwith disability. [file 12889_2022_13281_MOESM1_ESM.zip › Revised Table S2.docx]

| Table S2. The change of lung cancer incidence between 1990 and 2019 at 204 countries and territories. | | | | | | | | | | |
| --- | --- | --- | --- | --- | --- | --- | --- | --- | --- | --- |
|  |  | All-age Incidence | | |  |  | ASIR | | |  |
| Region | 1990 No. (95% UI) | | 2019 No. (95% UI) | Change in absolute number (95% UI) | | 1990 per 100,000 No. (95% UI) | | 2019 per 100,000 No. (95% UI) | EAPC No. (95% CI) | |
| Afghanistan | 947.81(469.46-1627.31) | | 1476.18(871.21-2335.11) | 0.56(0.08-1.35) | | 13.2(6.91-22.44) | | 11.74(7.33-18.53) | -0.4(-3.07,2.33) | |
| Albania | 622.4(580.81-665.03) | | 1174.38(861.5-1565.46) | 0.89(0.37-1.52) | | 29.74(27.72-31.8) | | 27.11(19.95-36.05) | -0.32(-2.09,1.49) | |
| Algeria | 1374.75(1101.44-1715.57) | | 3192.93(2459.09-4110.39) | 1.32(0.65-2.23) | | 11.76(9.57-14.55) | | 9.79(7.6-12.52) | -0.63(-3.49,2.32) | |
| American Samoa | 6.13(5.28-7) | | 12.4(10.63-14.19) | 1.02(0.65-1.46) | | 28.05(24.4-31.93) | | 26.51(22.95-30.08) | -0.19(-2,1.65) | |
| Andorra | 25.32(19.58-34.21) | | 55.08(42.58-69.51) | 1.18(0.49-2.03) | | 45.02(34.92-60.37) | | 39.49(30.5-49.93) | -0.45(-1.91,1.03) | |
| Angola | 574.24(379.38-810.01) | | 1566.39(1215.5-2010.16) | 1.73(0.85-3.17) | | 14.37(9.77-19.81) | | 14.04(11.32-17.62) | -0.08(-2.58,2.49) | |
| Antigua and Barbuda | 5.13(4.71-5.61) | | 9.52(8.15-11.05) | 0.86(0.55-1.21) | | 9.76(8.97-10.65) | | 9.5(8.18-11) | -0.09(-3.13,3.03) | |
| Argentina | 10073.61(9742.56-10415.08) | | 13856.2(10992.52-17375.09) | 0.38(0.08-0.73) | | 30.91(29.87-31.98) | | 25.89(20.52-32.54) | -0.61(-2.38,1.2) | |
| Armenia | 1108.25(1051.87-1166.45) | | 1345.78(1129.72-1595.96) | 0.21(0.01-0.45) | | 37.53(35.7-39.35) | | 31.8(26.74-37.68) | -0.57(-2.18,1.06) | |
| Australia | 7486.2(7211.1-7719.43) | | 12796.62(10031.21-16136.18) | 0.71(0.34-1.15) | | 37.87(36.46-39.06) | | 30.45(23.87-38.45) | -0.75(-2.37,0.9) | |
| Austria | 3485.17(3357.12-3597.19) | | 5117.19(4162.31-6261.27) | 0.47(0.2-0.8) | | 30.41(29.42-31.41) | | 30.48(24.69-37.43) | 0.01(-1.71,1.76) | |
| Azerbaijan | 1440.24(1315.47-1573.09) | | 2375.07(1746.37-3041.88) | 0.65(0.22-1.15) | | 26.57(24.33-28.83) | | 23.47(17.37-29.48) | -0.43(-2.32,1.5) | |
| Bahamas | 26.07(23.53-28.8) | | 57.32(46.67-71.27) | 1.2(0.73-1.78) | | 16.79(15.18-18.53) | | 14.49(11.86-17.87) | -0.51(-2.89,1.93) | |
| Bahrain | 59.49(50.38-69.12) | | 140.72(106.64-185.66) | 1.37(0.71-2.37) | | 41.42(35.26-47.78) | | 19.84(15.2-25.09) | -2.51(-4.29,-0.69) | |
| Bangladesh | 4245.72(3061.19-5535.46) | | 9652.31(6331.5-15119.7) | 1.27(0.48-2.27) | | 9.18(6.62-11.95) | | 7.43(4.91-11.56) | -0.73(-3.98,2.64) | |
| Barbados | 27.62(25.25-29.84) | | 47.88(39.19-56.96) | 0.73(0.41-1.1) | | 9.61(8.82-10.4) | | 9.69(7.96-11.54) | 0.03(-3,3.15) | |
| Belarus | 4529.17(4362.12-4690.53) | | 3801.17(2924.07-4935.88) | -0.16(-0.36-0.09) | | 34.09(32.88-35.26) | | 23.87(18.46-31.1) | -1.22(-2.99,0.58) | |
| Belgium | 7839.16(7538.58-8143.95) | | 8675.64(6828.49-10947.82) | 0.11(-0.13-0.4) | | 51.59(49.65-53.54) | | 39.84(31.05-50.57) | -0.89(-2.29,0.54) | |
| Belize | 9.46(8.48-10.44) | | 37.05(31.67-42.65) | 2.92(2.25-3.69) | | 10.29(9.24-11.37) | | 13.32(11.42-15.25) | 0.89(-1.9,3.76) | |
| Benin | 193.31(160.78-228.93) | | 485.42(369.95-634.17) | 1.51(0.88-2.34) | | 9.92(8.25-11.72) | | 10.52(8.15-13.6) | 0.2(-2.75,3.24) | |
| Bermuda | 24.15(22.5-26.01) | | 34.9(29.57-42.22) | 0.45(0.21-0.77) | | 38.82(36.22-41.74) | | 26.8(22.64-32.35) | -1.27(-2.93,0.42) | |
| Bhutan | 14.9(10.22-21.13) | | 42.59(30.65-58.41) | 1.86(0.99-3.17) | | 6.03(4.21-8.48) | | 7.73(5.57-10.59) | 0.86(-2.77,4.64) | |
| Bolivia (Plurinational State of) | 406.23(271.04-525.67) | | 1147.65(745.86-1579.35) | 1.83(1.09-2.81) | | 12.85(8.55-16.65) | | 13.37(8.62-18.31) | 0.14(-2.47,2.81) | |
| Bosnia and Herzegovina | 1652.44(1555.13-1748.3) | | 2434.6(1889.96-3062.17) | 0.47(0.13-0.86) | | 37.96(35.87-40.06) | | 40.26(31.35-50.7) | 0.2(-1.32,1.75) | |
| Botswana | 106.32(82.22-133.99) | | 298.44(206.58-397.98) | 1.81(0.96-2.97) | | 18.32(14.3-22.9) | | 21.73(15.57-28.61) | 0.59(-1.54,2.77) | |
| Brazil | 15433.82(14973.52-15826.56) | | 36410.39(34234.23-38236.42) | 1.36(1.24-1.48) | | 17.29(16.69-17.77) | | 15.4(14.45-16.17) | -0.4(-2.73,1.99) | |
| Brunei Darussalam | 40.11(32.74-47.4) | | 115.51(101.92-130.45) | 1.88(1.31-2.68) | | 46.62(38.32-55.13) | | 46.15(40.91-51.83) | -0.03(-1.43,1.38) | |
| Bulgaria | 3633.46(3422.3-3854.66) | | 4837.61(3859.19-6016.14) | 0.33(0.05-0.68) | | 28.59(26.92-30.29) | | 36.36(28.73-45.51) | 0.83(-0.86,2.55) | |
| Burkina Faso | 356.98(274.16-486.71) | | 875.54(682.5-1177.84) | 1.45(0.92-2.16) | | 8.35(6.43-11.35) | | 10.03(7.89-13.51) | 0.63(-2.5,3.87) | |
| Burundi | 192.13(144.65-250.41) | | 312.31(218.81-431.03) | 0.63(0.07-1.52) | | 8.22(6.24-10.67) | | 7.08(5-9.6) | -0.51(-3.9,2.99) | |
| Cabo Verde | 24.87(22.19-27.47) | | 70.86(55.69-85.9) | 1.85(1.2-2.5) | | 10.66(9.56-11.8) | | 17.29(13.48-21.08) | 1.68(-0.96,4.39) | |
| Cambodia | 1068.64(821.17-1345.01) | | 2887.6(2266.6-3576.17) | 1.7(0.93-2.61) | | 24(18.73-29.92) | | 24.71(19.4-30.59) | 0.1(-1.82,2.06) | |
| Cameroon | 482.29(376.82-613.35) | | 1560.22(1119.38-2154.77) | 2.24(1.24-3.58) | | 11.13(8.74-14.1) | | 13.73(9.95-18.61) | 0.73(-1.98,3.51) | |
| Canada | 16914.82(16347.1-17465.28) | | 30309.05(23639.56-38238.97) | 0.79(0.4-1.26) | | 52.15(50.42-53.77) | | 43.63(33.99-55.05) | -0.61(-1.98,0.77) | |
| Central African Republic | 178.04(92.4-293.1) | | 284.44(144.48-498.35) | 0.6(0.15-1.11) | | 14.37(8.06-23.36) | | 12.38(6.91-20.88) | -0.51(-3.09,2.13) | |
| Chad | 225.4(164.35-299.36) | | 624.56(446.58-887.92) | 1.77(1.04-2.64) | | 8.04(5.85-10.67) | | 11.74(8.48-16.51) | 1.31(-1.77,4.5) | |
| Chile | 1859.36(1786.52-1935.47) | | 4050.92(3180.67-5096.58) | 1.18(0.69-1.75) | | 18.64(17.9-19.4) | | 16.76(13.15-21.07) | -0.37(-2.61,1.93) | |
| China | 257044.68(221285.9-293647.85) | | 832922.16(700293.15-981631.63) | 2.24(1.59-3.11) | | 30.2(26.2-34.26) | | 41.71(35.22-48.8) | 1.12(-0.5,2.77) | |
| Colombia | 2694.12(2587.02-2795.69) | | 6399.64(5018.73-8152.48) | 1.38(0.86-2.02) | | 15.62(14.95-16.24) | | 12.14(9.52-15.47) | -0.87(-3.4,1.73) | |
| Comoros | 16.83(9.66-22.1) | | 35.3(26.23-46.98) | 1.1(0.41-2.62) | | 7.66(4.65-9.93) | | 7.37(5.52-9.75) | -0.13(-3.55,3.41) | |
| Congo | 197.87(113.57-296.65) | | 416.01(292.47-595.23) | 1.1(0.44-2.25) | | 18.02(10.83-26.28) | | 16.02(11.79-22.45) | -0.41(-2.69,1.93) | |
| Cook Islands | 4.44(3.77-5.18) | | 7.25(6.22-8.51) | 0.63(0.29-1.1) | | 35.93(30.72-41.7) | | 29.07(24.91-34.28) | -0.73(-2.39,0.96) | |
| Costa Rica | 194.48(182.83-205.3) | | 502.62(389.88-633.81) | 1.58(1.01-2.29) | | 11.38(10.68-12.02) | | 9.89(7.67-12.44) | -0.48(-3.36,2.48) | |
| Croatia | 2987.12(2775.11-3204.65) | | 3430.52(2706.26-4299.04) | 0.15(-0.1-0.45) | | 45.27(42.19-48.54) | | 40.39(31.62-51.05) | -0.39(-1.84,1.07) | |
| Cuba | 3428.88(3283.39-3556.31) | | 6827.11(5601.63-8297) | 0.99(0.63-1.4) | | 33.3(31.88-34.52) | | 35.88(29.37-43.68) | 0.26(-1.36,1.9) | |
| Cyprus | 157.94(135.88-181.63) | | 514.05(444.19-591.86) | 2.25(1.62-3.02) | | 19.32(16.76-22.17) | | 26.03(22.56-30.01) | 1.03(-1,3.11) | |
| Czechia | 6640.87(6445.5-6895.62) | | 6942.77(5695.06-8448.38) | 0.05(-0.15-0.29) | | 48.77(47.34-50.65) | | 32.89(26.85-40.09) | -1.35(-2.84,0.17) | |
| Côte d'Ivoire | 458.01(334.26-590.83) | | 1278.89(940.19-1651.18) | 1.79(1.07-2.8) | | 11.85(8.8-15.12) | | 12.85(9.67-16.29) | 0.28(-2.42,3.04) | |
| Democratic People's Republic of Korea | 4429.07(3372.79-5828.15) | | 9042.04(7008.19-11461.48) | 1.04(0.52-1.63) | | 26.58(20.76-34.2) | | 27.88(21.77-35.2) | 0.16(-1.65,2.02) | |
| Democratic Republic of the Congo | 2255.34(1245.44-5015.35) | | 4254.01(2251.37-8568.25) | 0.89(0.3-1.81) | | 14.26(8.22-31.21) | | 11.87(6.26-23.34) | -0.63(-3.24,2.04) | |
| Denmark | 4100.18(3962.42-4222.34) | | 4931.63(3886.79-6075.19) | 0.2(-0.06-0.5) | | 52.86(51.14-54.47) | | 42.91(33.7-53.14) | -0.72(-2.09,0.67) | |
| Djibouti | 12.28(7.86-18.63) | | 61.72(38.92-103.74) | 4.02(2.43-6.25) | | 8.98(5.91-13.38) | | 10.79(7.09-17.39) | 0.63(-2.39,3.75) | |
| Dominica | 11.43(10.12-12.98) | | 15.27(12.34-18.49) | 0.34(0.07-0.69) | | 16.11(14.2-18.36) | | 16.92(13.63-20.52) | 0.17(-2.16,2.55) | |
| Dominican Republic | 411.12(361.88-466.33) | | 1404.72(1015.12-1898.48) | 2.42(1.37-3.82) | | 11.11(9.78-12.6) | | 15.23(11.09-20.5) | 1.1(-1.56,3.83) | |
| Ecuador | 451.3(427.29-475.88) | | 1478.23(1167.64-1879.04) | 2.28(1.58-3.2) | | 8.58(8.1-9.07) | | 10.07(7.98-12.75) | 0.55(-2.56,3.76) | |
| Egypt | 2017.75(1838.15-2214.23) | | 6123(4303.05-8313.47) | 2.03(1.05-3.19) | | 6.57(5.96-7.19) | | 9.22(6.48-12.46) | 1.17(-2.26,4.72) | |
| El Salvador | 261.57(245.98-278.12) | | 619.47(469.27-797) | 1.37(0.78-2.1) | | 8.8(8.25-9.38) | | 10.45(7.87-13.47) | 0.59(-2.47,3.75) | |
| Equatorial Guinea | 26.44(13.89-44.64) | | 76.25(48.52-117.28) | 1.88(0.4-4.29) | | 12.85(7.09-21.26) | | 16.38(10.79-24.65) | 0.84(-1.67,3.41) | |
| Eritrea | 69.63(48.79-95.14) | | 226.34(173.13-293.37) | 2.25(1.25-3.74) | | 6.72(4.85-9.1) | | 8.44(6.52-10.59) | 0.79(-2.67,4.37) | |
| Estonia | 824.46(795.03-855.73) | | 721.23(563.53-905.64) | -0.13(-0.31-0.09) | | 39.89(38.49-41.41) | | 28.28(21.96-35.64) | -1.18(-2.81,0.48) | |
| Eswatini | 48.25(32.35-69.08) | | 103.54(62.86-146.63) | 1.15(0.49-2.18) | | 16.42(11.22-23.53) | | 17.78(11.08-24.87) | 0.27(-2.02,2.62) | |
| Ethiopia | 1272.37(811.1-2237.53) | | 2166.75(1512.97-2920.75) | 0.7(0.12-2.05) | | 6.5(4.08-11.35) | | 5.59(3.91-7.51) | -0.52(-4.33,3.43) | |
| Fiji | 36.54(29.09-45.21) | | 73.11(57.34-91.51) | 1(0.43-1.89) | | 10.52(8.36-13) | | 10.1(8.04-12.47) | -0.14(-3.07,2.88) | |
| Finland | 2400.38(2311.49-2480.58) | | 3113.54(2467.36-3909.51) | 0.3(0.02-0.64) | | 33.63(32.4-34.75) | | 25.16(19.81-31.68) | -1(-2.74,0.78) | |
| France | 26350.53(25493.06-27204.93) | | 45063.66(35105.75-57492.33) | 0.71(0.34-1.17) | | 33.75(32.7-34.79) | | 36.82(28.64-46.99) | 0.3(-1.3,1.93) | |
| Gabon | 108.21(63.57-162.75) | | 201.7(134.68-288.61) | 0.86(0.31-1.67) | | 18.85(11.29-27.99) | | 19.25(13.1-26.93) | 0.07(-2.09,2.29) | |
| Gambia | 20.54(15.5-26.07) | | 68.96(52.35-88.99) | 2.36(1.3-3.74) | | 6.02(4.65-7.53) | | 7.51(5.72-9.63) | 0.76(-2.89,4.56) | |
| Georgia | 1966.41(1786.73-2164.72) | | 1783.98(1487.37-2108.03) | -0.09(-0.26-0.1) | | 30.45(27.79-33.29) | | 31.14(25.89-36.87) | 0.08(-1.63,1.82) | |
| Germany | 41371.79(39948.38-42751.89) | | 63453.31(49670.58-81894.71) | 0.53(0.19-0.97) | | 33.67(32.54-34.73) | | 35.48(27.82-46.13) | 0.18(-1.43,1.82) | |
| Ghana | 509.75(412.77-623.59) | | 1377.25(1108.05-1728.74) | 1.7(0.98-2.63) | | 8.05(6.58-9.72) | | 8.7(7.09-10.83) | 0.27(-2.99,3.64) | |
| Greece | 6515.79(6240.8-6774.86) | | 9237.89(7274.26-11548.65) | 0.42(0.12-0.78) | | 42.58(40.79-44.29) | | 41.98(32.69-53.06) | -0.05(-1.51,1.43) | |
| Greenland | 29.85(26.75-33.59) | | 54.23(44.61-63.64) | 0.82(0.46-1.2) | | 85.59(77.79-95.54) | | 77.71(64.38-90.6) | -0.33(-1.38,0.73) | |
| Grenada | 10.54(9.52-11.65) | | 16.11(14.34-17.96) | 0.53(0.32-0.76) | | 14.97(13.48-16.59) | | 14.23(12.78-15.83) | -0.18(-2.64,2.35) | |
| Guam | 27.52(23.51-32.14) | | 59(49.41-70.21) | 1.14(0.7-1.71) | | 37.75(31.99-44.12) | | 31.12(26.08-36.97) | -0.66(-2.28,0.98) | |
| Guatemala | 334.66(295.38-378.54) | | 867.48(676.76-1094.53) | 1.59(0.99-2.37) | | 9.27(8.21-10.44) | | 7.87(6.16-9.88) | -0.56(-3.77,2.75) | |
| Guinea | 277.23(232.84-325.18) | | 549.35(420.74-706.8) | 0.98(0.48-1.67) | | 8.41(7.04-9.81) | | 10.11(7.83-12.93) | 0.64(-2.49,3.86) | |
| Guinea-Bissau | 55.8(32.8-79.35) | | 91.98(59.47-133.05) | 0.65(0.18-1.3) | | 13.81(8.38-19.35) | | 13.13(8.56-18.74) | -0.17(-2.74,2.46) | |
| Guyana | 32.02(27.53-36.5) | | 55.33(43.03-70.54) | 0.73(0.29-1.27) | | 8.4(7.26-9.54) | | 8.71(6.81-10.97) | 0.13(-3.09,3.45) | |
| Haiti | 447.98(268.37-723.01) | | 780.27(481.06-1268.87) | 0.74(0.21-1.61) | | 13.49(8.22-21.68) | | 11.33(7.19-18.39) | -0.6(-3.27,2.14) | |
| Honduras | 285.66(226.23-339.37) | | 1375.88(893.7-1965.13) | 3.82(2.28-5.82) | | 13.71(10.78-16.29) | | 23.04(15.01-32.67) | 1.81(-0.51,4.18) | |
| Hungary | 7148.45(6919.97-7363.55) | | 9509.8(7849.06-11561.74) | 0.33(0.09-0.61) | | 49.27(47.7-50.73) | | 51.94(42.61-63.65) | 0.18(-1.16,1.54) | |
| Iceland | 102.1(94.88-110.04) | | 187.39(163.01-214.58) | 0.84(0.57-1.12) | | 36.34(33.79-39.13) | | 34.55(30.17-39.47) | -0.17(-1.76,1.44) | |
| India | 29190.37(24752.05-34053.83) | | 87339.21(71865.33-103504.12) | 1.99(1.2-2.77) | | 6.62(5.61-7.71) | | 7.7(6.36-9.1) | 0.53(-3.01,4.19) | |
| Indonesia | 16681.89(14325.01-18960.74) | | 48198.9(35265.54-59309.34) | 1.89(1.14-2.62) | | 16.99(14.61-19.22) | | 22.87(16.78-28.04) | 1.03(-1.13,3.24) | |
| Iran (Islamic Republic of) | 2865.36(2392.93-3440.58) | | 8704.66(8039.65-9366.32) | 2.04(1.39-2.85) | | 11.07(9.1-13.35) | | 12.24(11.26-13.16) | 0.35(-2.43,3.2) | |
| Iraq | 1218.65(970.09-1506.25) | | 4154.17(3199.79-5128.84) | 2.41(1.51-3.65) | | 15.89(12.7-19.67) | | 18.69(14.53-22.68) | 0.56(-1.73,2.91) | |
| Ireland | 1650.02(1580.8-1714.22) | | 2500.48(1975.29-3141.06) | 0.52(0.2-0.92) | | 39.68(38.04-41.2) | | 33.21(26.16-41.73) | -0.61(-2.18,0.98) | |
| Israel | 1115.99(1061.86-1167.45) | | 2670.18(2087.83-3391.41) | 1.39(0.86-2.04) | | 23.26(22.1-24.29) | | 23.37(18.1-29.71) | 0.02(-1.94,2.02) | |
| Italy | 33921.17(33037.78-34638.61) | | 41734.88(34310.61-49564.07) | 0.23(0.01-0.46) | | 38.37(37.41-39.17) | | 30.16(24.64-36.01) | -0.83(-2.45,0.82) | |
| Jamaica | 268.38(253.3-283.93) | | 574.91(453.53-717.66) | 1.14(0.67-1.68) | | 15.42(14.57-16.32) | | 19.46(15.33-24.26) | 0.81(-1.49,3.16) | |
| Japan | 52538.08(50331.31-53952.14) | | 116798.01(95776.19-136540.9) | 1.22(0.87-1.6) | | 30.95(29.57-31.8) | | 30.66(25.55-36) | -0.03(-1.74,1.7) | |
| Jordan | 167.15(136.78-205.67) | | 914.37(748.42-1109.93) | 4.47(3.07-6.33) | | 12.49(10.18-15.37) | | 14.34(11.86-17.36) | 0.48(-2.12,3.14) | |
| Kazakhstan | 5680.08(5403.69-5989.24) | | 3829.27(3259.16-4405.84) | -0.33(-0.43--0.22) | | 42.5(40.43-44.73) | | 21.36(18.36-24.44) | -2.34(-4.08,-0.58) | |
| Kenya | 361.57(273.54-452.12) | | 1181.15(977-1425.67) | 2.27(1.62-3.2) | | 4.59(3.47-5.72) | | 5.62(4.71-6.72) | 0.7(-3.5,5.07) | |
| Kiribati | 7.48(6-9.11) | | 13.65(9.98-17.99) | 0.82(0.23-1.73) | | 19.29(15.69-23.28) | | 19.17(14.44-24.63) | -0.02(-2.18,2.18) | |
| Kuwait | 69.28(62.11-77.06) | | 225.33(184.58-271.79) | 2.25(1.68-2.99) | | 11.95(10.68-13.41) | | 10.1(8.19-12.19) | -0.58(-3.41,2.33) | |
| Kyrgyzstan | 835.69(786.39-888) | | 568.77(492.88-646.55) | -0.32(-0.42--0.22) | | 26.81(25.26-28.45) | | 12.2(10.62-13.86) | -2.68(-4.92,-0.38) | |
| Lao People's Democratic Republic | 540.22(376.2-756.09) | | 973.36(717.55-1264.48) | 0.8(0.18-1.56) | | 25.4(18.29-35.12) | | 22.78(17.04-29.01) | -0.38(-2.3,1.59) | |
| Latvia | 1318.87(1270.08-1369.35) | | 1017.98(837.52-1237.7) | -0.23(-0.37--0.06) | | 36.53(35.19-37.91) | | 26.92(21.98-33.01) | -1.05(-2.73,0.67) | |
| Lebanon | 482.16(360.23-615.31) | | 1421.22(1168.08-1867.91) | 1.95(1.09-3.55) | | 20.94(15.75-26.64) | | 27.29(22.42-35.83) | 0.92(-1.04,2.92) | |
| Lesotho | 122.56(95.58-175.17) | | 233.19(157.63-326.33) | 0.9(0.25-1.83) | | 12.26(9.61-17.52) | | 18.07(12.39-24.97) | 1.35(-1.16,3.91) | |
| Liberia | 106.61(83.21-131.08) | | 174.84(118.05-241.04) | 0.64(0.12-1.29) | | 9.64(7.64-11.82) | | 9.2(6.38-12.55) | -0.16(-3.22,3) | |
| Libya | 365.45(279.84-467.52) | | 924.91(688.44-1183.34) | 1.53(0.71-2.72) | | 19.87(15.19-25.29) | | 18.49(13.72-23.47) | -0.25(-2.4,1.95) | |
| Lithuania | 1584.07(1530.04-1638.82) | | 1395.18(1139.09-1690.82) | -0.12(-0.28-0.07) | | 34.81(33.65-35.99) | | 25.44(20.58-30.98) | -1.08(-2.8,0.68) | |
| Luxembourg | 239.45(226.46-254.14) | | 318.17(261.31-385.88) | 0.33(0.09-0.63) | | 44.29(41.89-46.94) | | 32.55(26.73-39.57) | -1.06(-2.59,0.5) | |
| Madagascar | 348.3(284.87-422.87) | | 677.57(495.24-898.74) | 0.95(0.37-1.67) | | 6.83(5.6-8.25) | | 6.36(4.75-8.3) | -0.25(-3.89,3.54) | |
| Malawi | 204.64(168.71-243.96) | | 415.85(326.32-520.7) | 1.03(0.58-1.57) | | 5.51(4.58-6.52) | | 5.94(4.72-7.28) | 0.25(-3.67,4.34) | |
| Malaysia | 1508.23(1324.23-1732.73) | | 5164.65(3997.2-6560.78) | 2.42(1.51-3.64) | | 17.02(14.78-19.66) | | 19.78(15.37-25.12) | 0.52(-1.7,2.79) | |
| Maldives | 11.06(7.52-14.76) | | 26.49(21.86-31.61) | 1.4(0.59-2.74) | | 13.83(9.95-18.4) | | 9.58(7.88-11.42) | -1.26(-4.02,1.59) | |
| Mali | 253.32(212.65-295.16) | | 595.24(454.29-776.2) | 1.35(0.72-2.16) | | 6.2(5.26-7.21) | | 7.2(5.52-9.29) | 0.52(-3.13,4.31) | |
| Malta | 115.37(107.48-124.14) | | 210.37(179.61-247.57) | 0.82(0.53-1.18) | | 26.68(24.84-28.72) | | 22.98(19.61-27.1) | -0.51(-2.41,1.42) | |
| Marshall Islands | 4.47(2.71-6.86) | | 10.03(6.08-14.94) | 1.24(0.63-2.04) | | 27.14(16.67-41.52) | | 30.36(19.15-44.33) | 0.39(-1.39,2.2) | |
| Mauritania | 106.05(83.09-128.99) | | 213.22(148.67-296.9) | 1.01(0.36-2.08) | | 10.62(8.32-12.88) | | 10.69(7.61-14.57) | 0.02(-2.87,2.99) | |
| Mauritius | 106.08(99.69-113.09) | | 206.51(168.74-249.99) | 0.95(0.55-1.38) | | 14.36(13.5-15.25) | | 11.84(9.72-14.28) | -0.66(-3.26,2.01) | |
| Mexico | 5841.02(5635.95-5959.84) | | 10890.36(9400.44-12582.5) | 0.86(0.61-1.14) | | 14.17(13.6-14.49) | | 9.52(8.23-10.97) | -1.36(-4.12,1.47) | |
| Micronesia (Federated States of) | 12.97(9.11-18.93) | | 21.8(12.7-32.8) | 0.68(0.1-1.44) | | 27.54(19.6-39.76) | | 31.29(19.17-45.55) | 0.44(-1.32,2.23) | |
| Monaco | 30.68(24.66-36.65) | | 67.54(55.14-80.16) | 1.2(0.69-1.9) | | 47.77(38.25-57.9) | | 75.57(61.39-90.82) | 1.59(0.33,2.87) | |
| Mongolia | 412.87(339.57-490.46) | | 662.38(512.2-863.46) | 0.6(0.19-1.19) | | 40.32(33.4-47.7) | | 31.19(24.67-39.68) | -0.88(-2.47,0.73) | |
| Montenegro | 307.84(269.34-346.18) | | 563.1(460.56-685.03) | 0.83(0.46-1.3) | | 48.2(42.22-53.95) | | 56.72(46.5-68.94) | 0.56(-0.76,1.9) | |
| Morocco | 2055.01(1569.6-2531.29) | | 5277.45(3793.49-6807.25) | 1.57(0.81-2.5) | | 14.6(11.04-17.9) | | 16.36(11.74-20.97) | 0.39(-2.02,2.87) | |
| Mozambique | 307.04(251.59-362.97) | | 862.18(642.51-1141.99) | 1.81(0.98-2.8) | | 5.45(4.53-6.43) | | 8.29(6.31-10.86) | 1.46(-2.25,5.31) | |
| Myanmar | 5795.98(4008.69-8844.24) | | 10291.14(7607.84-14071.02) | 0.78(0.26-1.63) | | 24.63(17.31-37.05) | | 22.43(16.85-30.17) | -0.32(-2.27,1.66) | |
| Namibia | 45.77(37.67-55.67) | | 106.57(83.47-134.82) | 1.33(0.77-2.11) | | 6.31(5.24-7.7) | | 7.77(6.2-9.69) | 0.72(-2.86,4.44) | |
| Nauru | 1.47(0.95-2.06) | | 1.55(0.93-2.13) | 0.05(-0.22-0.42) | | 39.68(27.04-54.28) | | 38.45(25.3-50.66) | -0.11(-1.63,1.43) | |
| Nepal | 662.85(444.89-937.63) | | 1759.15(1263.39-2275.02) | 1.65(0.86-2.78) | | 7.12(4.67-10.24) | | 8.03(5.86-10.37) | 0.41(-3.02,3.97) | |
| Netherlands | 9942.86(9617.95-10234.7) | | 15155.49(12033-19018.65) | 0.52(0.2-0.91) | | 50.22(48.58-51.69) | | 44.82(35.35-56.6) | -0.39(-1.77,1) | |
| New Zealand | 1523.65(1453.31-1590.69) | | 2504.6(2076.61-2953.82) | 0.64(0.36-0.97) | | 38.61(36.84-40.31) | | 31.95(26.44-37.83) | -0.65(-2.24,0.97) | |
| Nicaragua | 112.71(99.34-128.57) | | 363.63(295.2-441.82) | 2.23(1.52-3.06) | | 7.58(6.55-8.76) | | 8.68(7.14-10.41) | 0.47(-2.85,3.9) | |
| Niger | 226.27(156.54-315.38) | | 654.17(414.87-949.77) | 1.89(1.13-2.92) | | 8.39(5.81-11.48) | | 9.05(5.77-13.05) | 0.26(-2.93,3.56) | |
| Nigeria | 2664.14(1951.69-3561.02) | | 5886.98(4570.01-7545.31) | 1.21(0.54-2.13) | | 6.29(4.7-8.3) | | 7.5(5.92-9.46) | 0.61(-3,4.35) | |
| Niue | 0.59(0.5-0.72) | | 0.66(0.53-0.81) | 0.11(-0.16-0.45) | | 27.4(22.93-33.15) | | 30.45(24.62-37.5) | 0.36(-1.41,2.17) | |
| North Macedonia | 582.55(535.2-630.47) | | 1339.7(1025.41-1703.6) | 1.3(0.76-1.97) | | 29.51(27.1-31.85) | | 40.42(31.04-51.24) | 1.09(-0.55,2.76) | |
| Northern Mariana Islands | 9.31(7.89-11.36) | | 22.47(19.16-25.47) | 1.41(0.95-1.91) | | 55.9(48.24-65.55) | | 44.82(38.99-50.05) | -0.76(-2.09,0.59) | |
| Norway | 1812.44(1734.1-1871.88) | | 2959.2(2517.3-3446.85) | 0.63(0.4-0.9) | | 27.68(26.62-28.58) | | 30.97(26.34-36.07) | 0.39(-1.37,2.18) | |
| Oman | 59.08(42.87-76.5) | | 146.86(116.66-192.09) | 1.49(0.71-2.67) | | 9.21(6.77-11.74) | | 9.86(8.16-11.94) | 0.23(-2.82,3.39) | |
| Pakistan | 7903.97(6635.06-9209.85) | | 18401.25(13969.72-24265.21) | 1.33(0.68-2.3) | | 13.92(11.67-16.27) | | 16.43(12.47-21.49) | 0.57(-1.87,3.08) | |
| Palau | 4.15(3.3-5.24) | | 9.3(7.43-11.69) | 1.24(0.64-1.99) | | 42.5(33.91-53.83) | | 44.54(36.03-55.03) | 0.16(-1.28,1.62) | |
| Palestine | 173.16(129.77-229.39) | | 523.49(443.92-612.56) | 2.02(1.14-3.22) | | 20.21(15.26-26.61) | | 22.53(19.09-26.1) | 0.38(-1.68,2.48) | |
| Panama | 211.67(198.21-224.59) | | 447.42(345.67-571.57) | 1.11(0.62-1.72) | | 14.3(13.38-15.17) | | 10.85(8.38-13.84) | -0.95(-3.61,1.78) | |
| Papua New Guinea | 341.94(232.86-531.93) | | 996.08(693.93-1508.53) | 1.91(1.15-3.06) | | 18.75(12.9-29.05) | | 21.81(15.59-32.97) | 0.52(-1.6,2.68) | |
| Paraguay | 232.44(205.71-264.9) | | 863.23(652.65-1106.21) | 2.71(1.72-3.96) | | 10.56(9.33-12.03) | | 15.81(12.01-20.31) | 1.4(-1.29,4.16) | |
| Peru | 1767.84(1513.15-2058.71) | | 3343.67(2506.92-4401.88) | 0.89(0.37-1.62) | | 14.91(12.8-17.41) | | 10.49(7.85-13.83) | -1.21(-3.86,1.52) | |
| Philippines | 6862.82(6033.44-7764.5) | | 13827.24(11026.21-17100.02) | 1.01(0.54-1.7) | | 23.05(20.43-25.95) | | 17.71(14.24-21.75) | -0.91(-3,1.23) | |
| Poland | 19923.38(19574.45-20256.95) | | 30018.42(25154.02-35717.87) | 0.51(0.26-0.8) | | 45.05(44.24-45.79) | | 43.06(35.98-51.42) | -0.16(-1.58,1.29) | |
| Portugal | 2741.29(2645.77-2839.83) | | 4679.52(3656.48-5919.02) | 0.71(0.32-1.16) | | 19.71(19.03-20.41) | | 21.38(16.53-27.28) | 0.28(-1.81,2.42) | |
| Puerto Rico | 612.52(580.58-640.86) | | 880.92(689.89-1109.33) | 0.44(0.13-0.82) | | 16.83(15.96-17.64) | | 12.4(9.71-15.74) | -1.05(-3.52,1.49) | |
| Qatar | 16.69(12.51-21.66) | | 124.76(88.77-174.19) | 6.47(3.54-11.22) | | 17.63(13.48-22.88) | | 18.78(14.38-23.88) | 0.22(-2,2.49) | |
| Republic of Korea | 7551.17(7221.43-7985.81) | | 31824.76(26456.32-37538.08) | 3.21(2.51-4.06) | | 24.52(23.44-26.13) | | 35.61(29.58-41.94) | 1.3(-0.49,3.11) | |
| Republic of Moldova | 1411.31(1347.89-1474.23) | | 1067.45(919.87-1227.46) | -0.24(-0.35--0.13) | | 30.25(28.92-31.54) | | 18.27(15.74-20.98) | -1.72(-3.67,0.26) | |
| Romania | 7627.27(7351.33-7887.52) | | 11544.7(9482.85-14024.5) | 0.51(0.24-0.85) | | 26.18(25.28-27) | | 33.21(27.24-40.47) | 0.82(-0.94,2.62) | |
| Russian Federation | 62878.36(60889.02-64076.19) | | 58183.52(49720.66-67801.76) | -0.07(-0.21-0.08) | | 33.42(32.34-34.09) | | 24.53(20.96-28.58) | -1.06(-2.82,0.73) | |
| Rwanda | 258.12(204.29-320.13) | | 471.94(325.99-685.08) | 0.83(0.21-2) | | 8.96(7.14-11.03) | | 8.17(5.71-11.69) | -0.32(-3.52,3) | |
| Saint Kitts and Nevis | 4.78(4.33-5.22) | | 7.6(6.31-9.05) | 0.59(0.29-0.94) | | 12.87(11.71-13.99) | | 11.42(9.7-13.44) | -0.41(-3.11,2.36) | |
| Saint Lucia | 11.92(11-12.9) | | 26.38(22.32-31.06) | 1.21(0.84-1.64) | | 13.73(12.7-14.84) | | 12.24(10.4-14.35) | -0.4(-3.01,2.29) | |
| Saint Vincent and the Grenadines | 6.78(6.19-7.42) | | 13.84(12.05-15.89) | 1.04(0.75-1.4) | | 9.45(8.65-10.34) | | 10.22(8.91-11.7) | 0.27(-2.74,3.37) | |
| Samoa | 9.9(8.05-11.81) | | 16.2(12.56-20.59) | 0.64(0.23-1.21) | | 11.26(9.21-13.38) | | 11.21(8.73-13.94) | -0.02(-2.83,2.87) | |
| San Marino | 12.15(10.66-13.81) | | 22.61(17.15-29.58) | 0.86(0.38-1.52) | | 36.57(32.15-41.66) | | 36.85(27.73-48.61) | 0.03(-1.54,1.62) | |
| Sao Tome and Principe | 8.81(7.01-10.57) | | 18.94(14.68-23.8) | 1.15(0.62-1.85) | | 13.75(11.12-16.35) | | 18.89(14.86-23.45) | 1.1(-1.29,3.55) | |
| Saudi Arabia | 421.61(312.63-549.36) | | 1544.89(1187.06-1899.53) | 2.66(1.5-4.49) | | 7.51(5.61-9.68) | | 8.77(6.98-10.62) | 0.54(-2.79,3.97) | |
| Senegal | 341.06(254.35-439.84) | | 875.33(661.83-1143.76) | 1.57(0.91-2.54) | | 10.78(8.1-13.89) | | 12.19(9.38-15.83) | 0.42(-2.37,3.3) | |
| Serbia | 4738.2(3922.51-5204.92) | | 7699.56(6057.6-9693.98) | 0.62(0.24-1.11) | | 39.09(32.44-42.8) | | 49.38(38.82-62.4) | 0.81(-0.64,2.28) | |
| Seychelles | 10.53(9.24-11.74) | | 19.06(16.62-22.12) | 0.81(0.51-1.15) | | 18.61(16.33-20.78) | | 17.66(15.38-20.33) | -0.18(-2.4,2.09) | |
| Sierra Leone | 183.6(141.14-227.83) | | 360.83(257.49-482.92) | 0.97(0.43-1.62) | | 9.73(7.51-12.03) | | 10.52(7.51-13.87) | 0.27(-2.7,3.33) | |
| Singapore | 905.71(865.53-942.4) | | 2161.73(1723.69-2713.17) | 1.39(0.92-1.96) | | 42.72(40.64-44.53) | | 28.39(22.71-35.43) | -1.4(-3,0.23) | |
| Slovakia | 2747.52(2605.53-2890.45) | | 3129.61(2429.99-4052.56) | 0.14(-0.13-0.51) | | 45.85(43.5-48.17) | | 33.56(26.06-43.42) | -1.07(-2.58,0.46) | |
| Slovenia | 890.2(684.73-1141.29) | | 1395.08(1081.7-1823.05) | 0.57(0.05-1.22) | | 36.18(27.88-46.3) | | 33.89(26.01-44.24) | -0.22(-1.82,1.4) | |
| Solomon Islands | 41.05(22.25-68.18) | | 102.05(53.46-167.1) | 1.49(0.76-2.42) | | 29.21(17.07-47.43) | | 32.7(18.61-51.37) | 0.39(-1.32,2.13) | |
| Somalia | 164.89(108.91-255.77) | | 364.84(204.52-606.84) | 1.21(0.44-2.17) | | 6.67(4.51-10.11) | | 5.55(3.14-9.08) | -0.63(-4.42,3.3) | |
| South Africa | 4658.32(3876.65-6170.1) | | 8517.87(7629.81-9703.58) | 0.83(0.48-1.16) | | 22.01(18.22-29.39) | | 19.13(17.2-21.64) | -0.48(-2.56,1.64) | |
| South Sudan | 230.65(152.98-350.78) | | 318.24(207.94-454.24) | 0.38(-0.02-0.99) | | 9.85(6.57-15.08) | | 8.84(5.9-12.47) | -0.37(-3.44,2.8) | |
| Spain | 18341.92(17681.76-18944.38) | | 29523.44(23091.1-37488.3) | 0.61(0.26-1.06) | | 34.02(32.84-35.11) | | 33.19(25.86-42.5) | -0.08(-1.72,1.58) | |
| Sri Lanka | 851.04(745.83-955.83) | | 2506.52(1822.45-3413.75) | 1.95(1.09-3.05) | | 7.76(6.85-8.68) | | 9.71(7.08-13.11) | 0.78(-2.45,4.11) | |
| Sudan | 715.38(415.97-1303.48) | | 1524.61(982.7-2363.63) | 1.13(0.41-2.47) | | 7.71(4.56-14.04) | | 8.28(5.39-12.91) | 0.25(-3.09,3.7) | |
| Suriname | 34.15(31.02-37.35) | | 91.07(75.03-109.88) | 1.67(1.15-2.26) | | 13.17(12.05-14.41) | | 15.1(12.46-18.2) | 0.47(-2.06,3.07) | |
| Sweden | 2975.04(2853.57-3082.49) | | 4342.07(3694.92-5018.2) | 0.46(0.25-0.68) | | 20.74(19.93-21.45) | | 20.78(17.67-24.16) | 0.01(-2.07,2.13) | |
| Switzerland | 3985.43(3839.95-4131.22) | | 4368.85(3411.5-5522.19) | 0.1(-0.13-0.39) | | 40.04(38.55-41.46) | | 26.16(20.26-33.07) | -1.46(-3.12,0.23) | |
| Syrian Arab Republic | 543.74(417.9-684.5) | | 1372.09(1006.81-1813.76) | 1.52(0.71-2.72) | | 10.09(7.75-12.72) | | 11.09(8.24-14.52) | 0.33(-2.58,3.32) | |
| Taiwan (Province of China) | 4021.02(3901.14-4135.87) | | 12617.6(9962.81-16206.11) | 2.14(1.47-3.01) | | 24.87(24.07-25.62) | | 31.88(25.21-40.93) | 0.86(-0.95,2.7) | |
| Tajikistan | 570.68(522.19-630.37) | | 593.28(478.94-740.28) | 0.04(-0.17-0.31) | | 19.46(17.86-21.47) | | 11.98(9.76-14.83) | -1.66(-4.07,0.81) | |
| Thailand | 10486.35(9341.75-11684.74) | | 22545.27(17018.46-29559.74) | 1.15(0.58-1.87) | | 28.89(25.73-32.25) | | 22.24(16.78-29.04) | -0.9(-2.77,1.01) | |
| Timor-Leste | 44.57(33.84-59.34) | | 158.32(112.77-207.23) | 2.55(1.41-4.08) | | 16.24(12.41-21.36) | | 19.48(13.86-25.27) | 0.63(-1.63,2.94) | |
| Togo | 122.71(99.12-152.76) | | 392.3(287.32-522.86) | 2.2(1.3-3.31) | | 10.24(8.35-12.65) | | 11.19(8.34-14.71) | 0.31(-2.58,3.28) | |
| Tokelau | 0.3(0.23-0.38) | | 0.32(0.25-0.42) | 0.09(-0.2-0.45) | | 21.6(17.07-27.42) | | 24.41(19.2-31.41) | 0.42(-1.56,2.45) | |
| Tonga | 14.51(11.41-18.17) | | 21.61(17.7-26.12) | 0.49(0.11-1) | | 26.87(21.12-33.62) | | 27.76(22.81-33.47) | 0.11(-1.7,1.96) | |
| Trinidad and Tobago | 98.91(92.85-105.23) | | 192(145.1-250.39) | 0.94(0.45-1.56) | | 11.74(11.01-12.51) | | 10.29(7.77-13.4) | -0.45(-3.28,2.46) | |
| Tunisia | 962.08(769.47-1160.53) | | 2461.92(1744.5-3393.33) | 1.56(0.69-2.76) | | 19.13(15.32-23.04) | | 19.37(13.86-26.55) | 0.04(-2.11,2.25) | |
| Turkey | 13982.78(10936.49-17375.57) | | 29510.56(23370.09-36799.05) | 1.11(0.49-1.94) | | 37.91(29.95-46.82) | | 33.08(26.25-41.11) | -0.47(-2.06,1.14) | |
| Turkmenistan | 354.53(336.95-371.49) | | 412.75(326.1-520.92) | 0.16(-0.09-0.47) | | 16.95(16.14-17.76) | | 9.9(7.84-12.37) | -1.84(-4.46,0.85) | |
| Tuvalu | 1.67(1.29-2.45) | | 2.63(1.94-3.57) | 0.57(0.14-1.18) | | 23.96(18.48-34.61) | | 25.98(19.42-35.01) | 0.28(-1.62,2.22) | |
| Uganda | 378.03(307.06-452.2) | | 1006.4(801.19-1212.47) | 1.66(1.03-2.5) | | 5.94(4.85-7.03) | | 7.34(5.95-8.68) | 0.73(-2.96,4.56) | |
| Ukraine | 31106.93(30002.77-32139.34) | | 20132.69(16536.45-24383.32) | -0.35(-0.47--0.22) | | 42.79(41.25-44.19) | | 27.19(22.38-33.08) | -1.55(-3.17,0.09) | |
| United Arab Emirates | 62.08(46.32-79.83) | | 541.68(393.37-721.35) | 7.73(4.66-12.57) | | 19.16(14.25-24.68) | | 18.62(13.54-25.75) | -0.1(-2.27,2.12) | |
| United Kingdom | 45591.53(44273.48-46372.04) | | 51489.73(43155.66-60653.97) | 0.13(-0.05-0.33) | | 49.95(48.58-50.78) | | 40.16(33.54-47.33) | -0.75(-2.16,0.68) | |
| United Republic of Tanzania | 887.25(651.53-1195.21) | | 2130.97(1530.89-3072.58) | 1.4(0.84-2.05) | | 8.21(6.08-10.95) | | 8.99(6.57-12.76) | 0.31(-2.91,3.64) | |
| United States of America | 184614.66(178855.66-188221.07) | | 254807.52(220998.46-294428.74) | 0.38(0.2-0.59) | | 58.87(57.24-59.96) | | 45.13(39.11-52.21) | -0.91(-2.23,0.42) | |
| United States Virgin Islands | 15.07(12.56-18.03) | | 45.12(37.77-52.24) | 1.99(1.39-2.8) | | 17.74(14.95-21.23) | | 24.07(20.11-27.83) | 1.06(-1.06,3.22) | |
| Uruguay | 1672.31(1596.65-1751.15) | | 1748.41(1377.52-2190.25) | 0.05(-0.19-0.32) | | 43.5(41.56-45.51) | | 34.13(26.64-42.94) | -0.83(-2.35,0.71) | |
| Uzbekistan | 2160.17(2064.92-2255.33) | | 2770.81(2282.13-3324.29) | 0.28(0.06-0.54) | | 18.12(17.35-18.9) | | 12.67(10.65-14.92) | -1.23(-3.64,1.25) | |
| Vanuatu | 13.31(8.4-21.14) | | 41.84(27.69-62.54) | 2.14(1.23-4.11) | | 20.95(13.21-32.94) | | 24.75(16.44-36.32) | 0.58(-1.42,2.61) | |
| Venezuela (Bolivarian Republic of) | 1820.92(1742.88-1898.63) | | 5767.99(4263.15-7448.9) | 2.17(1.36-3.13) | | 18.71(17.85-19.54) | | 19.78(14.72-25.4) | 0.19(-1.97,2.4) | |
| Viet Nam | 8743.67(6880.97-10720.22) | | 25549.85(19741.34-32387.02) | 1.92(1.12-2.97) | | 21.32(16.78-26.04) | | 26.72(20.83-33.51) | 0.78(-1.18,2.78) | |
| Yemen | 466.7(297.07-735.91) | | 1302.23(885.33-1929.71) | 1.79(0.93-3.31) | | 9.4(6.1-14.63) | | 9.95(6.8-14.67) | 0.19(-2.84,3.32) | |
| Zambia | 276.85(222.64-347.89) | | 699.21(500.32-910.93) | 1.53(0.81-2.4) | | 10(8.04-12.54) | | 10.73(7.9-13.78) | 0.24(-2.69,3.27) | |
| Zimbabwe | 549.61(484.28-621.26) | | 1024.42(785.91-1244.53) | 0.86(0.43-1.35) | | 13.42(11.84-15.12) | | 14.53(11.32-17.55) | 0.27(-2.26,2.87) | |
| Abbreviations: ASIR, age standardized incidence rate; UI, uncertainty interval; EAPC, estimated annual percentage change; CI, confidence interval. | | | | | | | | | | |
