# Supplementary material for: The spatiotemporal dynamics of lung cancer: 30-year trends of epidemiology across 204 countries and territories
Source: BMC Public Health. 2022 May 16;22:987. doi: 10.1186/s12889-022-13281-y (PMC9109351; doi:10.1186/s12889-022-13281-y)
Supplement: Supplementary file 1 — Additional file 1: TableS1.The change of lung cancer prevalence between 1990 and 2019 at 204 countries andterritories. Table S2. The change oflung cancer incidence between 1990 and 2019 at 204 countries and territories. Table S3. The change of lung cancerYLDs between 1990 and 2019 at 204 countries and territories. YLDs, years livedwith disability. [file 12889_2022_13281_MOESM1_ESM.zip › Revised Table S3.docx]

| Table S3. The change of lung cancer YLDs between 1990 and 2019 at 204 countries and territories. | | | | | | | | | | |
| --- | --- | --- | --- | --- | --- | --- | --- | --- | --- | --- |
|  |  | All-age YLDs | | |  |  | ASYR | | |  |
| Region | 1990 No. (95% UI) | | 2019 No. (95% UI) | Change in absolute number (95% UI) | | 1990 per 100,000 No. (95% UI) | | 2019 per 100,000 No. (95% UI) | EAPC No. (95% CI) | |
| Afghanistan | 217.02(93.34-420.67) | | 345.82(177.19-583.85) | 0.59(0.06-1.58) | | 2.97(1.33-5.66) | | 2.62(1.44-4.41) | -0.43(-5.97,5.44) | |
| Albania | 141.99(99.97-187.79) | | 269.13(166.28-405.31) | 0.9(0.31-1.61) | | 6.67(4.71-8.75) | | 6.26(3.87-9.45) | -0.22(-3.9,3.61) | |
| Algeria | 321.63(202.91-463.31) | | 750.17(486.97-1095.14) | 1.33(0.57-2.41) | | 2.67(1.71-3.78) | | 2.25(1.45-3.25) | -0.59(-6.49,5.68) | |
| American Samoa | 1.39(0.95-1.84) | | 2.75(1.95-3.68) | 0.98(0.53-1.56) | | 6.13(4.2-8.08) | | 5.75(4.1-7.71) | -0.22(-4.06,3.78) | |
| Andorra | 5.99(3.96-8.8) | | 13.78(8.96-19.42) | 1.3(0.54-2.32) | | 10.53(7.02-15.43) | | 9.9(6.43-13.93) | -0.21(-3.15,2.82) | |
| Angola | 134.89(76.4-215.38) | | 367.3(238.25-541.89) | 1.72(0.75-3.39) | | 3.28(1.91-5.15) | | 3.17(2.09-4.59) | -0.12(-5.3,5.35) | |
| Antigua and Barbuda | 1.19(0.81-1.61) | | 2.25(1.5-3.06) | 0.9(0.45-1.48) | | 2.29(1.57-3.11) | | 2.21(1.47-2.98) | -0.12(-6.29,6.45) | |
| Argentina | 2308.28(1616.37-2982.58) | | 3166.39(2055.69-4556.45) | 0.37(0.02-0.83) | | 7.08(4.94-9.14) | | 5.96(3.84-8.59) | -0.59(-4.26,3.21) | |
| Armenia | 257.98(181.01-341.52) | | 306.99(214.18-415.51) | 0.19(-0.08-0.5) | | 8.63(6.09-11.33) | | 7.26(5.01-9.87) | -0.59(-3.92,2.85) | |
| Australia | 1793.71(1290.12-2294.99) | | 3289.49(2235.33-4678.83) | 0.83(0.38-1.37) | | 9.12(6.54-11.71) | | 7.98(5.41-11.39) | -0.46(-3.67,2.86) | |
| Austria | 817.71(586.27-1055.22) | | 1295.94(875.43-1789.09) | 0.58(0.22-1.03) | | 7.27(5.23-9.45) | | 7.92(5.33-10.86) | 0.3(-3.13,3.84) | |
| Azerbaijan | 341.99(233.73-454.73) | | 562.84(346.47-816.76) | 0.65(0.12-1.3) | | 6.22(4.29-8.32) | | 5.42(3.39-7.73) | -0.47(-4.35,3.56) | |
| Bahamas | 6.12(4.24-8.17) | | 13.65(9.05-18.91) | 1.23(0.65-2.02) | | 3.88(2.69-5.17) | | 3.39(2.27-4.71) | -0.46(-5.34,4.67) | |
| Bahrain | 13.06(8.86-17.3) | | 32.61(21.35-48.65) | 1.5(0.71-2.7) | | 8.61(5.9-11.32) | | 4.15(2.76-5.86) | -2.48(-6.34,1.53) | |
| Bangladesh | 997.86(596.1-1492.65) | | 2280.53(1252.41-4049.16) | 1.29(0.37-2.53) | | 2.12(1.25-3.2) | | 1.74(0.96-3.07) | -0.69(-7.33,6.42) | |
| Barbados | 6.33(4.32-8.49) | | 11.25(7.43-15.53) | 0.78(0.33-1.3) | | 2.25(1.54-3.05) | | 2.29(1.51-3.15) | 0.06(-6.09,6.61) | |
| Belarus | 1039.86(727.72-1350.29) | | 889.54(574.52-1271.17) | -0.14(-0.38-0.16) | | 7.83(5.5-10.15) | | 5.61(3.61-8.05) | -1.14(-4.77,2.62) | |
| Belgium | 1782.41(1284.72-2317.87) | | 2055.1(1357.43-2935.18) | 0.15(-0.12-0.51) | | 11.88(8.55-15.44) | | 9.7(6.38-13.98) | -0.7(-3.56,2.25) | |
| Belize | 2.22(1.52-3.03) | | 8.82(5.92-12.07) | 2.98(2.01-4.09) | | 2.4(1.65-3.28) | | 3.11(2.08-4.27) | 0.9(-4.8,6.93) | |
| Benin | 44.44(29.57-60.89) | | 112.49(72.04-165.12) | 1.53(0.81-2.6) | | 2.26(1.49-3.09) | | 2.37(1.52-3.43) | 0.17(-5.93,6.67) | |
| Bermuda | 5.53(3.85-7.26) | | 8.35(5.73-11.43) | 0.51(0.19-0.94) | | 8.83(6.16-11.55) | | 6.52(4.48-8.95) | -1.04(-4.44,2.47) | |
| Bhutan | 3.62(2.02-5.69) | | 9.94(6.19-15.08) | 1.75(0.82-3.15) | | 1.43(0.82-2.22) | | 1.78(1.11-2.7) | 0.76(-6.61,8.71) | |
| Bolivia (Plurinational State of) | 92.85(50.79-138.83) | | 260.67(151.82-400.67) | 1.81(0.91-2.99) | | 2.88(1.58-4.32) | | 2.99(1.73-4.62) | 0.13(-5.31,5.88) | |
| Bosnia and Herzegovina | 380.94(273.11-499.27) | | 548.22(350.37-785.91) | 0.44(0.05-0.9) | | 8.6(6.16-11.22) | | 9.13(5.82-12.99) | 0.21(-2.96,3.48) | |
| Botswana | 24.1(15.57-35.16) | | 68.28(41.81-103.99) | 1.83(0.82-3.26) | | 4.06(2.66-5.89) | | 4.81(2.95-7.2) | 0.59(-3.89,5.27) | |
| Brazil | 3524.15(2494.44-4482.26) | | 8344.36(5981.28-10706.4) | 1.37(1.19-1.55) | | 3.87(2.75-4.91) | | 3.51(2.51-4.5) | -0.34(-5.18,4.75) | |
| Brunei Darussalam | 9.09(6.14-12.04) | | 27.44(19.35-36.46) | 2.02(1.33-3.03) | | 10.02(6.81-13.36) | | 10.25(7.29-13.39) | 0.08(-2.88,3.13) | |
| Bulgaria | 836(592.28-1102.33) | | 1116.79(742.86-1571.9) | 0.34(0-0.76) | | 6.61(4.69-8.71) | | 8.55(5.57-12.08) | 0.89(-2.58,4.48) | |
| Burkina Faso | 83.27(51.33-121.8) | | 202.88(128.3-301.98) | 1.44(0.76-2.4) | | 1.91(1.18-2.81) | | 2.26(1.44-3.35) | 0.57(-5.89,7.48) | |
| Burundi | 45.14(28.41-64.94) | | 75.07(45.01-116.62) | 0.66(0.03-1.64) | | 1.91(1.21-2.76) | | 1.65(0.99-2.52) | -0.49(-7.39,6.91) | |
| Cabo Verde | 5.71(4.03-7.62) | | 15.95(10.34-21.94) | 1.79(1.06-2.73) | | 2.47(1.74-3.29) | | 3.84(2.48-5.32) | 1.54(-3.91,7.3) | |
| Cambodia | 240.81(156.11-342.14) | | 649.54(419.97-907.18) | 1.7(0.87-2.79) | | 5.27(3.45-7.46) | | 5.43(3.56-7.61) | 0.1(-3.95,4.33) | |
| Cameroon | 111.17(70.48-158.72) | | 362.21(217.38-558.04) | 2.26(1.12-3.76) | | 2.49(1.6-3.55) | | 3.08(1.88-4.61) | 0.73(-4.9,6.7) | |
| Canada | 4050.06(2932.27-5241.99) | | 7744.18(5129.07-10899.36) | 0.91(0.45-1.51) | | 12.55(9.07-16.22) | | 11.31(7.43-16.03) | -0.36(-3.08,2.44) | |
| Central African Republic | 41.32(18.76-75.64) | | 67.45(30.33-129.81) | 0.63(0.09-1.33) | | 3.26(1.6-5.83) | | 2.85(1.38-5.26) | -0.46(-5.77,5.15) | |
| Chad | 52.25(31.17-79.85) | | 143.13(86.15-222.87) | 1.74(0.91-2.9) | | 1.85(1.1-2.83) | | 2.62(1.57-4.04) | 1.2(-5.16,8) | |
| Chile | 424(298.5-556.31) | | 929.9(603.86-1333.79) | 1.19(0.61-1.93) | | 4.21(2.97-5.51) | | 3.85(2.5-5.53) | -0.31(-4.95,4.55) | |
| China | 59316.18(41687.83-77778.55) | | 199351.51(138983.38-264035.88) | 2.36(1.65-3.3) | | 6.81(4.78-8.9) | | 9.84(6.88-12.99) | 1.28(-2.08,4.75) | |
| Colombia | 620.4(439.8-830.23) | | 1506.29(961.77-2199.1) | 1.43(0.77-2.22) | | 3.52(2.48-4.71) | | 2.86(1.82-4.15) | -0.72(-5.92,4.77) | |
| Comoros | 3.93(2.05-6) | | 8.29(5.26-12.56) | 1.11(0.36-2.89) | | 1.77(0.97-2.68) | | 1.71(1.07-2.58) | -0.13(-7.11,7.38) | |
| Congo | 45.09(21.47-75.8) | | 96.8(56.72-154.46) | 1.15(0.36-2.59) | | 4.01(2.01-6.68) | | 3.59(2.17-5.62) | -0.38(-5.15,4.63) | |
| Cook Islands | 0.99(0.67-1.36) | | 1.6(1.1-2.16) | 0.61(0.2-1.14) | | 7.88(5.36-10.7) | | 6.43(4.4-8.69) | -0.7(-4.2,2.93) | |
| Costa Rica | 44.92(31.35-59.93) | | 118.97(74.52-173.1) | 1.65(0.9-2.51) | | 2.6(1.81-3.49) | | 2.33(1.47-3.39) | -0.39(-6.28,5.88) | |
| Croatia | 699.64(488.17-925.17) | | 835.54(552.3-1173.56) | 0.19(-0.11-0.61) | | 10.58(7.39-13.98) | | 10.03(6.57-14.15) | -0.19(-3.11,2.83) | |
| Cuba | 773.78(538.18-998.11) | | 1563.3(1062.88-2173.2) | 1.02(0.56-1.56) | | 7.52(5.22-9.71) | | 8.27(5.61-11.55) | 0.33(-3.03,3.8) | |
| Cyprus | 35.82(25.2-47.54) | | 124.12(88.25-167.66) | 2.46(1.67-3.53) | | 4.36(3.06-5.74) | | 6.32(4.5-8.52) | 1.29(-2.89,5.64) | |
| Czechia | 1505.8(1079.85-1949.71) | | 1603.63(1072.79-2221.38) | 0.06(-0.18-0.37) | | 11.13(7.92-14.4) | | 7.74(5.19-10.73) | -1.25(-4.32,1.93) | |
| Côte d'Ivoire | 106.73(64.25-158.14) | | 297.07(192.37-434.24) | 1.78(0.91-3.06) | | 2.64(1.61-3.88) | | 2.87(1.85-4.17) | 0.28(-5.34,6.23) | |
| Democratic People's Republic of Korea | 1043.9(658.64-1535.64) | | 2083.5(1352.59-2984.96) | 1(0.43-1.74) | | 6.11(3.96-8.84) | | 6.38(4.17-9.12) | 0.15(-3.61,4.05) | |
| Democratic Republic of the Congo | 520.84(243.62-1244.33) | | 989.03(458.71-2131.01) | 0.9(0.25-2.01) | | 3.19(1.49-7.44) | | 2.67(1.23-5.58) | -0.6(-6.02,5.13) | |
| Denmark | 933.5(674.78-1193.61) | | 1179.54(795.92-1659.6) | 0.26(-0.03-0.63) | | 12.25(8.86-15.64) | | 10.5(6.99-14.82) | -0.53(-3.32,2.34) | |
| Djibouti | 2.96(1.68-4.85) | | 14.6(7.44-25.72) | 3.93(2.04-6.75) | | 2.09(1.19-3.39) | | 2.45(1.27-4.28) | 0.55(-5.65,7.16) | |
| Dominica | 2.54(1.72-3.39) | | 3.44(2.26-4.87) | 0.36(0.02-0.84) | | 3.64(2.5-4.87) | | 3.83(2.53-5.42) | 0.17(-4.66,5.25) | |
| Dominican Republic | 96.85(66.45-131.62) | | 327(202.47-494.01) | 2.38(1.21-4.01) | | 2.56(1.75-3.5) | | 3.51(2.19-5.3) | 1.09(-4.37,6.86) | |
| Ecuador | 106.27(72.27-143.39) | | 339.5(220.77-482.34) | 2.19(1.37-3.24) | | 1.99(1.35-2.69) | | 2.28(1.49-3.24) | 0.48(-5.9,7.29) | |
| Egypt | 492.2(333.18-656.37) | | 1475.3(859.49-2283.7) | 2(0.93-3.39) | | 1.57(1.05-2.11) | | 2.16(1.25-3.31) | 1.1(-5.81,8.53) | |
| El Salvador | 61.41(42.31-82.49) | | 144.28(90.99-209.43) | 1.35(0.65-2.23) | | 2.04(1.4-2.75) | | 2.44(1.53-3.56) | 0.62(-5.63,7.28) | |
| Equatorial Guinea | 6.1(2.78-11.36) | | 17.61(9.65-28.74) | 1.89(0.43-4.65) | | 2.91(1.4-5.27) | | 3.65(2.04-5.78) | 0.79(-4.43,6.29) | |
| Eritrea | 16.81(9.4-26.2) | | 54.45(34.93-79.33) | 2.24(1.14-3.94) | | 1.57(0.91-2.4) | | 1.96(1.26-2.8) | 0.76(-6.28,8.32) | |
| Estonia | 185.51(135.24-242.49) | | 163.17(107.96-233.29) | -0.12(-0.33-0.14) | | 9(6.59-11.71) | | 6.54(4.33-9.39) | -1.1(-4.47,2.4) | |
| Eswatini | 11.05(6.41-17.01) | | 23.45(12.44-37.37) | 1.12(0.41-2.32) | | 3.66(2.14-5.59) | | 3.93(2.13-6.15) | 0.25(-4.56,5.29) | |
| Ethiopia | 297.41(154.59-573.39) | | 509.96(297.61-761.84) | 0.71(0.09-2.15) | | 1.48(0.79-2.8) | | 1.29(0.75-1.93) | -0.49(-8.26,7.95) | |
| Fiji | 8.58(5.57-12.39) | | 16.93(10.81-24.33) | 0.97(0.37-1.92) | | 2.37(1.53-3.38) | | 2.26(1.46-3.22) | -0.16(-6.24,6.32) | |
| Finland | 570.79(413.57-729.93) | | 770.54(513.06-1089.36) | 0.35(0.03-0.78) | | 8.08(5.87-10.32) | | 6.39(4.23-9.04) | -0.8(-4.29,2.81) | |
| France | 6116.21(4311-7900.82) | | 11165.9(7338.34-15731.64) | 0.83(0.37-1.41) | | 7.95(5.67-10.33) | | 9.41(6.16-13.41) | 0.58(-2.64,3.91) | |
| Gabon | 24.66(12.46-41.53) | | 46.44(26.51-72.99) | 0.88(0.26-1.92) | | 4.24(2.19-7.05) | | 4.32(2.51-6.64) | 0.06(-4.46,4.79) | |
| Gambia | 4.91(3.11-7.26) | | 16.18(10.19-23.76) | 2.29(1.18-4.08) | | 1.41(0.91-2.06) | | 1.73(1.11-2.53) | 0.71(-6.72,8.72) | |
| Georgia | 461.63(323.52-614.41) | | 405.58(275.92-552.45) | -0.12(-0.33-0.13) | | 7.13(4.97-9.52) | | 7.15(4.84-9.78) | 0.01(-3.5,3.65) | |
| Germany | 9999.8(7158.52-12934.31) | | 15664.54(10466.9-22084.57) | 0.57(0.17-1.07) | | 8.25(5.89-10.68) | | 9.07(6.06-12.93) | 0.33(-2.88,3.64) | |
| Ghana | 122.79(82.19-172.63) | | 326.52(209.93-468.51) | 1.66(0.82-2.79) | | 1.88(1.26-2.62) | | 2.01(1.3-2.88) | 0.22(-6.42,7.34) | |
| Greece | 1510.19(1069.17-1952.77) | | 2173.21(1423.32-3070.77) | 0.44(0.09-0.86) | | 9.94(7.01-12.81) | | 10.22(6.65-14.57) | 0.1(-2.87,3.16) | |
| Greenland | 6.71(4.82-8.9) | | 12.11(8.23-16.27) | 0.8(0.42-1.27) | | 18.6(13.36-24.32) | | 16.96(11.61-22.67) | -0.32(-2.55,1.97) | |
| Grenada | 2.35(1.63-3.14) | | 3.75(2.58-5.02) | 0.59(0.27-1.01) | | 3.4(2.37-4.56) | | 3.26(2.27-4.4) | -0.14(-5.24,5.23) | |
| Guam | 6.35(4.39-8.57) | | 13.44(9.03-18.58) | 1.12(0.63-1.8) | | 8.35(5.79-11.18) | | 7.04(4.79-9.69) | -0.59(-3.97,2.91) | |
| Guatemala | 79.43(54.25-106.39) | | 201.92(131.05-294.05) | 1.54(0.77-2.46) | | 2.12(1.44-2.86) | | 1.8(1.17-2.61) | -0.56(-7.14,6.48) | |
| Guinea | 65.17(42.99-90.02) | | 128.53(82.86-189.18) | 0.97(0.34-1.82) | | 1.95(1.3-2.68) | | 2.32(1.5-3.39) | 0.6(-5.79,7.42) | |
| Guinea-Bissau | 12.75(6.28-19.92) | | 21.28(11.87-34.58) | 0.67(0.1-1.52) | | 3.09(1.58-4.76) | | 2.94(1.66-4.68) | -0.18(-5.53,5.48) | |
| Guyana | 7.56(5.14-10.36) | | 13.12(8.14-19.18) | 0.74(0.21-1.44) | | 1.95(1.33-2.66) | | 2.02(1.25-2.93) | 0.13(-6.44,7.16) | |
| Haiti | 104.68(52.97-181.54) | | 182.72(101.08-315.4) | 0.75(0.16-1.74) | | 3.08(1.57-5.33) | | 2.58(1.44-4.4) | -0.62(-6.13,5.22) | |
| Honduras | 67.17(44.26-93.22) | | 313.04(177.61-491.6) | 3.66(2.06-5.85) | | 3.17(2.07-4.39) | | 5.15(2.92-8.04) | 1.69(-3.1,6.72) | |
| Hungary | 1622.26(1162.68-2089.44) | | 2173.52(1496.36-2962.28) | 0.34(0.06-0.68) | | 11.25(8.08-14.51) | | 12.09(8.24-16.64) | 0.25(-2.52,3.1) | |
| Iceland | 24.53(17.34-32.34) | | 48.31(33.47-64.74) | 0.97(0.62-1.41) | | 8.83(6.23-11.63) | | 9.05(6.23-12.14) | 0.09(-3.06,3.34) | |
| India | 6897.52(4716.28-9362.99) | | 20367.52(14189.78-27535.34) | 1.95(1.16-2.72) | | 1.51(1.02-2.05) | | 1.77(1.23-2.38) | 0.54(-6.72,8.35) | |
| Indonesia | 3825.32(2634.6-5094.96) | | 10918.72(6937.99-15201.44) | 1.85(1.07-2.63) | | 3.77(2.61-4.98) | | 5.01(3.21-6.99) | 0.99(-3.56,5.75) | |
| Iran (Islamic Republic of) | 675.48(459.05-918.52) | | 2023.34(1445.19-2616.98) | 2(1.35-2.74) | | 2.5(1.72-3.4) | | 2.78(1.99-3.59) | 0.36(-5.38,6.45) | |
| Iraq | 277.25(180.62-389.97) | | 957.44(629.52-1387.75) | 2.45(1.37-3.97) | | 3.55(2.3-4.97) | | 4.17(2.74-5.92) | 0.56(-4.23,5.59) | |
| Ireland | 371.03(263.03-474.91) | | 604.19(402.57-849.39) | 0.63(0.22-1.12) | | 9.01(6.4-11.55) | | 8.11(5.4-11.42) | -0.36(-3.57,2.95) | |
| Israel | 255.69(181.88-333.12) | | 633.62(413.76-901.61) | 1.48(0.82-2.3) | | 5.36(3.8-7.01) | | 5.62(3.66-8.02) | 0.16(-3.84,4.33) | |
| Italy | 7852.05(5700.56-9991.22) | | 10069.35(7037.6-13529.42) | 0.28(0.05-0.54) | | 8.98(6.51-11.42) | | 7.57(5.24-10.21) | -0.59(-3.85,2.78) | |
| Jamaica | 62.14(43.46-82.53) | | 132.39(85.02-191.41) | 1.13(0.55-1.92) | | 3.6(2.52-4.77) | | 4.47(2.89-6.47) | 0.75(-3.96,5.69) | |
| Japan | 13493.51(9903.92-16990.17) | | 32090(22515.24-42645.86) | 1.38(1-1.81) | | 7.9(5.81-9.96) | | 8.93(6.31-11.83) | 0.42(-2.84,3.79) | |
| Jordan | 39.46(26.47-55.53) | | 216.36(142.14-305.8) | 4.48(2.91-6.69) | | 2.85(1.94-3.99) | | 3.26(2.17-4.61) | 0.47(-4.89,6.13) | |
| Kazakhstan | 1316.32(915.49-1730) | | 886.63(608.48-1203.93) | -0.33(-0.47--0.16) | | 9.76(6.8-12.8) | | 4.88(3.34-6.58) | -2.37(-5.96,1.36) | |
| Kenya | 84.79(54.59-121.53) | | 281.09(190.13-392.38) | 2.32(1.65-3.24) | | 1.05(0.68-1.51) | | 1.3(0.88-1.81) | 0.73(-7.81,10.05) | |
| Kiribati | 1.73(1.17-2.41) | | 3.17(1.99-4.79) | 0.84(0.19-1.85) | | 4.31(2.92-5.93) | | 4.28(2.76-6.33) | -0.03(-4.54,4.69) | |
| Kuwait | 16.67(11.48-22.49) | | 53.59(36.47-74.87) | 2.21(1.39-3.22) | | 2.75(1.88-3.71) | | 2.28(1.52-3.22) | -0.64(-6.48,5.56) | |
| Kyrgyzstan | 195.19(136.47-258.11) | | 135.63(92.3-187.42) | -0.31(-0.47--0.13) | | 6.22(4.34-8.21) | | 2.84(1.92-3.92) | -2.67(-7.26,2.16) | |
| Lao People's Democratic Republic | 121.73(71.02-189.92) | | 220.41(135.86-318.98) | 0.81(0.17-1.67) | | 5.61(3.36-8.5) | | 5.01(3.1-7.19) | -0.39(-4.45,3.83) | |
| Latvia | 299.47(209.19-388.92) | | 236.21(161.52-331.71) | -0.21(-0.4-0.01) | | 8.31(5.81-10.8) | | 6.37(4.37-8.96) | -0.91(-4.38,2.68) | |
| Lebanon | 109.04(68.94-158.72) | | 319.65(213.78-461.41) | 1.93(0.97-3.55) | | 4.65(2.96-6.68) | | 6.15(4.11-8.88) | 0.97(-3.14,5.25) | |
| Lesotho | 28.19(17.69-42.92) | | 52.75(30.15-80.8) | 0.87(0.17-1.98) | | 2.78(1.75-4.22) | | 4(2.33-6.07) | 1.27(-3.94,6.75) | |
| Liberia | 24.39(15.98-35.32) | | 40.88(24.06-62.05) | 0.68(0.1-1.45) | | 2.18(1.43-3.14) | | 2.08(1.24-3.16) | -0.17(-6.5,6.59) | |
| Libya | 83.5(54.89-118.94) | | 211.57(137.55-305.84) | 1.53(0.66-2.77) | | 4.45(2.93-6.31) | | 4.11(2.67-5.94) | -0.27(-4.78,4.45) | |
| Lithuania | 360.8(256.19-477.89) | | 320.29(218.53-443.07) | -0.11(-0.31-0.15) | | 7.94(5.63-10.49) | | 5.94(4.02-8.28) | -0.99(-4.56,2.7) | |
| Luxembourg | 55.18(39.35-71.75) | | 77.78(51.71-107.19) | 0.41(0.12-0.8) | | 10.29(7.3-13.4) | | 8.06(5.37-11.09) | -0.84(-3.94,2.36) | |
| Madagascar | 82.7(52.58-116.55) | | 164.55(103.94-247.36) | 0.99(0.36-1.89) | | 1.59(1.01-2.24) | | 1.5(0.94-2.25) | -0.21(-7.61,7.77) | |
| Malawi | 48.48(31.84-68.11) | | 99.49(63.77-144.06) | 1.05(0.48-1.82) | | 1.28(0.83-1.78) | | 1.4(0.91-1.99) | 0.3(-7.66,8.95) | |
| Malaysia | 341.16(235.44-462.66) | | 1176.14(742.18-1703.49) | 2.45(1.38-3.85) | | 3.76(2.55-5.14) | | 4.41(2.81-6.35) | 0.55(-4.11,5.44) | |
| Maldives | 2.59(1.43-3.99) | | 6.22(4.23-8.68) | 1.41(0.53-2.94) | | 3.06(1.8-4.65) | | 2.17(1.46-3.02) | -1.19(-6.94,4.92) | |
| Mali | 60.28(40.27-84.24) | | 140.4(89.2-205.21) | 1.33(0.6-2.36) | | 1.45(0.98-2.01) | | 1.66(1.07-2.42) | 0.48(-6.96,8.5) | |
| Malta | 26.33(18.79-34.7) | | 48.92(33.15-66.42) | 0.86(0.44-1.32) | | 6.1(4.35-8.02) | | 5.47(3.69-7.42) | -0.37(-4.26,3.68) | |
| Marshall Islands | 1.01(0.52-1.66) | | 2.28(1.19-3.71) | 1.26(0.56-2.23) | | 5.96(3.12-9.77) | | 6.58(3.55-10.48) | 0.34(-3.42,4.25) | |
| Mauritania | 24.66(15.88-35.39) | | 49.45(28.43-75.43) | 1(0.24-2.33) | | 2.44(1.57-3.48) | | 2.43(1.43-3.62) | -0.02(-5.96,6.29) | |
| Mauritius | 24.12(16.63-32.35) | | 47.43(31.3-66.86) | 0.97(0.47-1.62) | | 3.2(2.21-4.25) | | 2.69(1.78-3.76) | -0.6(-6,5.11) | |
| Mexico | 1320.26(942.35-1681.02) | | 2525.01(1761.28-3332.92) | 0.91(0.65-1.21) | | 3.13(2.23-3.98) | | 2.18(1.52-2.88) | -1.23(-6.95,4.83) | |
| Micronesia (Federated States of) | 2.95(1.77-4.64) | | 5.01(2.52-8.28) | 0.7(0.05-1.56) | | 6.11(3.72-9.5) | | 6.9(3.72-11.06) | 0.42(-3.28,4.26) | |
| Monaco | 7.1(4.86-9.73) | | 16.16(11.13-21.86) | 1.28(0.71-2.09) | | 11.42(7.74-15.65) | | 18.75(12.59-25.59) | 1.72(-0.82,4.34) | |
| Mongolia | 91.86(61.81-126.91) | | 150.83(97.87-218.46) | 0.64(0.15-1.35) | | 8.85(5.96-12.2) | | 6.83(4.47-9.72) | -0.89(-4.24,2.58) | |
| Montenegro | 71.88(51.12-95.12) | | 129.94(85.64-179.75) | 0.81(0.39-1.33) | | 11.17(8.01-14.72) | | 13.17(8.67-18.21) | 0.57(-2.16,3.37) | |
| Morocco | 477.42(306.72-668) | | 1219.57(746.93-1807.89) | 1.55(0.66-2.72) | | 3.35(2.16-4.71) | | 3.71(2.33-5.46) | 0.36(-4.63,5.6) | |
| Mozambique | 72.29(46.59-100.24) | | 200.33(122.18-301.64) | 1.77(0.84-3.03) | | 1.25(0.81-1.72) | | 1.87(1.16-2.81) | 1.4(-6.22,9.63) | |
| Myanmar | 1310.59(785.89-2173.45) | | 2328.96(1471.64-3601.85) | 0.78(0.19-1.67) | | 5.45(3.31-8.97) | | 4.97(3.19-7.61) | -0.32(-4.41,3.95) | |
| Namibia | 10.77(6.89-15.54) | | 24.73(15.48-36) | 1.3(0.64-2.4) | | 1.47(0.97-2.1) | | 1.77(1.12-2.53) | 0.64(-6.66,8.52) | |
| Nauru | 0.34(0.2-0.55) | | 0.36(0.19-0.58) | 0.06(-0.25-0.49) | | 8.76(5.26-13.44) | | 8.38(4.75-12.57) | -0.16(-3.36,3.16) | |
| Nepal | 156.48(87.9-248.45) | | 412.71(253.56-614.19) | 1.64(0.67-2.9) | | 1.64(0.91-2.66) | | 1.85(1.14-2.74) | 0.41(-6.61,7.95) | |
| Netherlands | 2312.65(1659.15-2967.52) | | 3683.4(2485.24-5155.65) | 0.59(0.22-1.05) | | 11.8(8.47-15.19) | | 11.12(7.43-15.71) | -0.2(-2.98,2.65) | |
| New Zealand | 349.94(252.08-445.65) | | 624.18(439.21-832.96) | 0.78(0.43-1.19) | | 8.92(6.44-11.39) | | 8.07(5.66-10.8) | -0.35(-3.57,2.98) | |
| Nicaragua | 26.63(17.66-37.31) | | 85.41(56.79-119.39) | 2.21(1.36-3.29) | | 1.76(1.16-2.49) | | 1.99(1.31-2.77) | 0.44(-6.35,7.72) | |
| Niger | 53.71(31.43-84.51) | | 153.64(83.87-242.75) | 1.86(0.93-3.14) | | 1.94(1.13-3.01) | | 2.05(1.12-3.24) | 0.19(-6.36,7.21) | |
| Nigeria | 623(382.74-940.13) | | 1375.74(879.58-1970.49) | 1.21(0.53-2.15) | | 1.45(0.89-2.15) | | 1.7(1.11-2.4) | 0.56(-6.84,8.55) | |
| Niue | 0.13(0.09-0.18) | | 0.14(0.1-0.2) | 0.11(-0.19-0.49) | | 6.03(4.07-8.24) | | 6.63(4.53-9.22) | 0.33(-3.42,4.21) | |
| North Macedonia | 135.05(95.15-177.48) | | 309.54(204.23-443.17) | 1.29(0.69-2.13) | | 6.78(4.79-8.89) | | 9.34(6.15-13.35) | 1.11(-2.28,4.62) | |
| Northern Mariana Islands | 2.19(1.5-3.07) | | 5.16(3.6-6.92) | 1.36(0.79-2.15) | | 12.31(8.63-16.92) | | 9.84(6.94-13.03) | -0.77(-3.59,2.14) | |
| Norway | 423.02(308.82-533.02) | | 753.56(529.17-995.74) | 0.78(0.5-1.11) | | 6.63(4.82-8.35) | | 8.03(5.63-10.63) | 0.66(-2.85,4.3) | |
| Oman | 13.95(8.58-20.83) | | 35.38(22.71-52.66) | 1.54(0.68-2.93) | | 2.1(1.3-3.12) | | 2.21(1.46-3.15) | 0.18(-6.14,6.92) | |
| Pakistan | 1804.12(1202.76-2440.16) | | 4258.89(2794.58-6129.06) | 1.36(0.62-2.4) | | 3.14(2.1-4.24) | | 3.69(2.42-5.32) | 0.56(-4.52,5.92) | |
| Palau | 0.92(0.6-1.32) | | 2.1(1.43-2.99) | 1.28(0.57-2.19) | | 9.23(6.02-13.13) | | 9.68(6.66-13.46) | 0.16(-2.91,3.32) | |
| Palestine | 39.03(24.4-58.09) | | 119.68(81.65-162.68) | 2.07(1.07-3.52) | | 4.47(2.83-6.6) | | 4.96(3.4-6.72) | 0.36(-3.97,4.88) | |
| Panama | 48.84(33.18-64.55) | | 105.49(67.11-153.73) | 1.16(0.56-1.92) | | 3.27(2.2-4.33) | | 2.56(1.62-3.73) | -0.85(-6.29,4.9) | |
| Papua New Guinea | 78.57(46.53-128.96) | | 228.17(130.62-364.24) | 1.9(1.07-3.27) | | 4.14(2.44-6.81) | | 4.76(2.81-7.5) | 0.49(-3.98,5.16) | |
| Paraguay | 54.43(36.96-73.73) | | 197.42(123.93-290.25) | 2.63(1.54-4.11) | | 2.45(1.66-3.31) | | 3.57(2.26-5.25) | 1.32(-4.21,7.16) | |
| Peru | 411.18(281.45-565.41) | | 777.07(484.51-1157.59) | 0.89(0.3-1.67) | | 3.41(2.34-4.69) | | 2.43(1.51-3.62) | -1.16(-6.61,4.61) | |
| Philippines | 1566.3(1090.69-2027.3) | | 3186.74(2119.98-4428.65) | 1.03(0.53-1.73) | | 5.12(3.59-6.6) | | 3.98(2.67-5.49) | -0.86(-5.24,3.72) | |
| Poland | 4415.32(3165.2-5622.08) | | 6569.69(4446.7-8871.31) | 0.49(0.24-0.77) | | 10(7.15-12.72) | | 9.53(6.45-12.93) | -0.17(-3.18,2.93) | |
| Portugal | 617.48(431.06-818.6) | | 1067.25(690.71-1551.33) | 0.73(0.24-1.33) | | 4.47(3.14-5.92) | | 5.02(3.23-7.32) | 0.4(-3.92,4.91) | |
| Puerto Rico | 140.14(98.13-183.68) | | 206.43(133.24-293.29) | 0.47(0.06-0.95) | | 3.86(2.72-5.01) | | 2.99(1.92-4.26) | -0.88(-5.91,4.42) | |
| Qatar | 3.93(2.46-5.68) | | 30.46(18.89-47.9) | 6.75(3.57-12.45) | | 3.81(2.43-5.38) | | 3.96(2.61-5.78) | 0.13(-4.61,5.1) | |
| Republic of Korea | 1805.62(1291.38-2395.7) | | 8770.88(6055.44-11978.9) | 3.86(2.75-5.2) | | 5.72(4.09-7.55) | | 9.76(6.77-13.33) | 1.86(-1.7,5.55) | |
| Republic of Moldova | 324.69(228.14-421.47) | | 249.5(170.32-348.2) | -0.23(-0.39--0.04) | | 6.94(4.9-9.01) | | 4.29(2.93-5.94) | -1.65(-5.64,2.52) | |
| Romania | 1773.78(1235.79-2344.33) | | 2669.03(1820.1-3667.51) | 0.5(0.14-0.93) | | 6.1(4.3-8.1) | | 7.8(5.29-10.84) | 0.85(-2.77,4.6) | |
| Russian Federation | 14332.09(10422.2-18188.54) | | 13686.73(9517.07-18088.91) | -0.05(-0.19-0.11) | | 7.62(5.52-9.69) | | 5.81(4.04-7.66) | -0.93(-4.55,2.83) | |
| Rwanda | 60.29(37.57-88.47) | | 111.31(64.1-183.11) | 0.85(0.15-2.24) | | 2.05(1.32-2.97) | | 1.87(1.1-3.06) | -0.32(-6.9,6.72) | |
| Saint Kitts and Nevis | 1.09(0.75-1.44) | | 1.82(1.21-2.59) | 0.67(0.24-1.24) | | 2.97(2.06-3.92) | | 2.66(1.81-3.72) | -0.38(-5.91,5.47) | |
| Saint Lucia | 2.71(1.9-3.62) | | 6.18(4.14-8.64) | 1.28(0.76-1.91) | | 3.11(2.19-4.14) | | 2.84(1.92-3.98) | -0.31(-5.68,5.38) | |
| Saint Vincent and the Grenadines | 1.56(1.08-2.09) | | 3.24(2.23-4.46) | 1.07(0.58-1.68) | | 2.18(1.51-2.93) | | 2.38(1.65-3.25) | 0.29(-5.86,6.85) | |
| Samoa | 2.31(1.5-3.29) | | 3.82(2.41-5.47) | 0.65(0.18-1.38) | | 2.59(1.7-3.68) | | 2.59(1.66-3.69) | 0(-5.76,6.11) | |
| San Marino | 2.83(1.99-3.8) | | 5.45(3.54-7.96) | 0.92(0.37-1.68) | | 8.6(6.07-11.6) | | 9.16(5.92-13.5) | 0.21(-2.95,3.48) | |
| Sao Tome and Principe | 1.99(1.31-2.8) | | 4.32(2.82-6.15) | 1.17(0.55-2.09) | | 3.06(2.02-4.25) | | 4.17(2.73-5.96) | 1.07(-3.94,6.35) | |
| Saudi Arabia | 100.22(63.46-146.24) | | 382.08(252.06-553.89) | 2.81(1.52-4.95) | | 1.74(1.11-2.54) | | 2.02(1.31-2.9) | 0.52(-6.27,7.81) | |
| Senegal | 78.9(50.52-115.13) | | 199.5(124.99-293.79) | 1.53(0.73-2.71) | | 2.45(1.58-3.55) | | 2.71(1.72-3.98) | 0.35(-5.45,6.51) | |
| Serbia | 1095.48(765.53-1442.07) | | 1770.51(1174.47-2490.33) | 0.62(0.19-1.22) | | 8.98(6.26-11.8) | | 11.55(7.62-16.25) | 0.87(-2.12,3.95) | |
| Seychelles | 2.34(1.61-3.12) | | 4.32(2.94-5.78) | 0.84(0.43-1.4) | | 4.15(2.85-5.55) | | 3.92(2.67-5.2) | -0.2(-4.84,4.67) | |
| Sierra Leone | 42.09(27.26-60.78) | | 83.11(50.45-124.63) | 0.97(0.35-1.8) | | 2.21(1.43-3.2) | | 2.36(1.44-3.54) | 0.23(-5.91,6.77) | |
| Singapore | 217.25(156.05-281.3) | | 609.71(414.16-849.56) | 1.81(1.18-2.67) | | 10(7.21-12.96) | | 7.89(5.36-10.98) | -0.81(-3.96,2.43) | |
| Slovakia | 662.67(472.51-859.47) | | 787.63(508.69-1132.43) | 0.19(-0.13-0.58) | | 11.1(7.88-14.44) | | 8.51(5.48-12.22) | -0.91(-3.92,2.19) | |
| Slovenia | 204.14(130.83-295.62) | | 332.41(217.43-468.18) | 0.63(0.07-1.38) | | 8.3(5.3-11.98) | | 8.28(5.43-11.73) | -0.01(-3.27,3.37) | |
| Solomon Islands | 9.52(4.39-17.22) | | 23.63(10.76-41.35) | 1.48(0.71-2.58) | | 6.53(3.23-11.5) | | 7.24(3.59-12.31) | 0.36(-3.24,4.09) | |
| Somalia | 39.45(21.68-65.65) | | 87.93(41.88-159.58) | 1.23(0.34-2.49) | | 1.55(0.86-2.52) | | 1.3(0.63-2.36) | -0.58(-8.26,7.74) | |
| South Africa | 1062.82(688.52-1593.3) | | 1923.55(1343.18-2569.77) | 0.81(0.47-1.17) | | 4.94(3.18-7.46) | | 4.25(2.98-5.65) | -0.52(-4.87,4.03) | |
| South Sudan | 53.04(30.22-87.95) | | 73.35(40.92-114.88) | 0.38(-0.08-1.12) | | 2.23(1.28-3.68) | | 1.97(1.11-3.03) | -0.42(-6.79,6.37) | |
| Spain | 4402.48(3198.12-5738.91) | | 7497.18(4926.45-10690.55) | 0.7(0.28-1.23) | | 8.26(6.01-10.81) | | 8.66(5.63-12.5) | 0.16(-3.08,3.51) | |
| Sri Lanka | 203.59(137.88-275.79) | | 594.4(357.87-901.65) | 1.92(0.97-3.29) | | 1.82(1.23-2.43) | | 2.28(1.37-3.45) | 0.79(-5.77,7.8) | |
| Sudan | 168.48(80.19-332.99) | | 363.74(211.27-610.57) | 1.16(0.37-2.65) | | 1.79(0.86-3.52) | | 1.92(1.12-3.28) | 0.25(-6.55,7.54) | |
| Suriname | 7.93(5.42-10.73) | | 21(13.76-29.49) | 1.65(0.99-2.52) | | 3.02(2.06-4.1) | | 3.44(2.27-4.84) | 0.45(-4.76,5.95) | |
| Sweden | 671.39(478.81-878.48) | | 978.15(686.28-1316.92) | 0.46(0.16-0.78) | | 4.8(3.41-6.24) | | 4.83(3.37-6.46) | 0.02(-4.24,4.47) | |
| Switzerland | 928.52(670.03-1191.24) | | 1070.55(704.44-1529.39) | 0.15(-0.13-0.54) | | 9.47(6.84-12.14) | | 6.57(4.33-9.36) | -1.25(-4.58,2.2) | |
| Syrian Arab Republic | 130.54(84.44-187.14) | | 327.33(200.65-485.27) | 1.51(0.65-2.89) | | 2.36(1.51-3.39) | | 2.57(1.59-3.72) | 0.3(-5.63,6.6) | |
| Taiwan (Province of China) | 940.21(661.47-1253.66) | | 2967.56(1984.24-4190.62) | 2.16(1.39-3.14) | | 5.71(4.05-7.58) | | 7.51(5.05-10.57) | 0.95(-2.77,4.81) | |
| Tajikistan | 135.71(92.22-178.73) | | 143.33(93-206.53) | 0.06(-0.22-0.43) | | 4.57(3.12-5.99) | | 2.76(1.83-3.92) | -1.73(-6.66,3.47) | |
| Thailand | 2406.84(1671.14-3190.57) | | 5127.12(3158.71-7512.38) | 1.13(0.47-2.03) | | 6.46(4.54-8.54) | | 5.03(3.14-7.33) | -0.86(-4.76,3.21) | |
| Timor-Leste | 10.37(6.66-15.54) | | 35.37(21.73-51.56) | 2.41(1.22-4.19) | | 3.59(2.34-5.36) | | 4.27(2.68-6.17) | 0.6(-4.15,5.59) | |
| Togo | 28.8(18.66-41.23) | | 92.23(58.61-138.47) | 2.2(1.18-3.63) | | 2.34(1.52-3.38) | | 2.54(1.63-3.77) | 0.28(-5.68,6.62) | |
| Tokelau | 0.07(0.04-0.09) | | 0.07(0.05-0.11) | 0.09(-0.23-0.58) | | 4.79(3.05-6.83) | | 5.37(3.5-7.91) | 0.4(-3.78,4.75) | |
| Tonga | 3.26(2.18-4.64) | | 4.79(3.16-6.67) | 0.47(0.06-1.06) | | 5.91(3.96-8.5) | | 6.11(4.03-8.49) | 0.11(-3.71,4.09) | |
| Trinidad and Tobago | 23.04(15.98-30.37) | | 45.56(28.32-68.06) | 0.98(0.36-1.77) | | 2.71(1.88-3.57) | | 2.44(1.52-3.62) | -0.36(-6.14,5.76) | |
| Tunisia | 217.57(142.55-304.77) | | 556.78(329.68-849.95) | 1.56(0.65-3.01) | | 4.25(2.79-5.93) | | 4.33(2.59-6.57) | 0.06(-4.45,4.78) | |
| Turkey | 3194.22(2098.11-4492.72) | | 6701.5(4547.53-9477.92) | 1.1(0.43-2.06) | | 8.52(5.68-11.9) | | 7.46(5.05-10.51) | -0.46(-3.78,2.97) | |
| Turkmenistan | 84.89(59.21-112.29) | | 101.55(65.45-147.24) | 0.2(-0.15-0.64) | | 4.01(2.79-5.3) | | 2.39(1.54-3.42) | -1.77(-7.04,3.81) | |
| Tuvalu | 0.38(0.24-0.6) | | 0.59(0.37-0.88) | 0.55(0.1-1.2) | | 5.35(3.44-8.35) | | 5.71(3.58-8.54) | 0.23(-3.77,4.39) | |
| Uganda | 89.31(57.47-124.56) | | 238.9(152.41-336.46) | 1.68(0.93-2.82) | | 1.38(0.89-1.92) | | 1.69(1.08-2.39) | 0.7(-6.81,8.82) | |
| Ukraine | 7363.59(5256.33-9409.34) | | 5007(3353.86-6839.24) | -0.32(-0.48--0.14) | | 10.17(7.25-12.98) | | 6.83(4.58-9.31) | -1.36(-4.6,1.99) | |
| United Arab Emirates | 14.56(9.29-21.06) | | 130.88(79.82-199.43) | 7.99(4.45-13.53) | | 4.09(2.57-5.92) | | 3.96(2.41-6.22) | -0.11(-4.76,4.76) | |
| United Kingdom | 10364.88(7488.83-13047.34) | | 12524.11(8851.02-16513.23) | 0.21(0.02-0.42) | | 11.51(8.35-14.47) | | 9.96(7.04-13.24) | -0.5(-3.37,2.45) | |
| United Republic of Tanzania | 208.52(124.21-319.31) | | 497.35(298.08-817.67) | 1.39(0.66-2.29) | | 1.9(1.15-2.88) | | 2.05(1.23-3.33) | 0.26(-6.33,7.32) | |
| United States of America | 43933.95(32386.68-54836.91) | | 61842.82(43850.82-80364.53) | 0.41(0.22-0.63) | | 14.19(10.45-17.73) | | 11.09(7.86-14.45) | -0.85(-3.5,1.88) | |
| United States Virgin Islands | 3.53(2.44-4.9) | | 10.22(7-14.1) | 1.89(1.17-2.9) | | 4.07(2.82-5.62) | | 5.49(3.73-7.63) | 1.03(-3.33,5.6) | |
| Uruguay | 381.05(270.86-492.05) | | 397.54(254.51-568.27) | 0.04(-0.23-0.37) | | 9.98(7.06-12.89) | | 7.89(5.05-11.4) | -0.81(-3.95,2.44) | |
| Uzbekistan | 515.63(358.46-690.76) | | 672.72(444.33-942.78) | 0.3(-0.02-0.71) | | 4.27(2.95-5.67) | | 2.94(1.96-4.05) | -1.28(-6.21,3.91) | |
| Vanuatu | 3.01(1.71-5.1) | | 9.42(5.57-15.06) | 2.13(1.1-4.33) | | 4.6(2.57-7.67) | | 5.43(3.24-8.71) | 0.58(-3.64,4.98) | |
| Venezuela (Bolivarian Republic of) | 423.49(298.71-554.55) | | 1340.12(870.42-1991.13) | 2.16(1.28-3.28) | | 4.28(2.98-5.59) | | 4.55(2.98-6.75) | 0.22(-4.24,4.88) | |
| Viet Nam | 1993.61(1318.27-2777.1) | | 5927.12(3923.01-8367.67) | 1.97(1.06-3.29) | | 4.83(3.21-6.69) | | 6.08(4.03-8.53) | 0.8(-3.27,5.04) | |
| Yemen | 110.49(59.12-190.96) | | 308.48(181.41-504.79) | 1.79(0.77-3.49) | | 2.18(1.2-3.73) | | 2.29(1.35-3.77) | 0.18(-6.03,6.79) | |
| Zambia | 64.86(41.67-92.55) | | 164.92(104.68-246.51) | 1.54(0.74-2.63) | | 2.28(1.48-3.23) | | 2.45(1.56-3.61) | 0.25(-5.79,6.68) | |
| Zimbabwe | 127.41(86.18-173.51) | | 237.14(156.93-339.33) | 0.86(0.33-1.55) | | 3.05(2.06-4.13) | | 3.27(2.18-4.68) | 0.24(-5.01,5.78) | |
| Abbreviations: YLDs, years lived with disability; ASYR, age standardized YLDs rate; UI, uncertainty interval; EAPC, estimated annual percentage change; CI, confidence interval. | | | | | | | | | | |
