# Supplementary figures and images for: The spatiotemporal dynamics of lung cancer: 30-year trends of epidemiology across 204 countries and territories
Source: BMC Public Health. 2022 May 16;22:987. doi: 10.1186/s12889-022-13281-y (PMC9109351; doi:10.1186/s12889-022-13281-y)

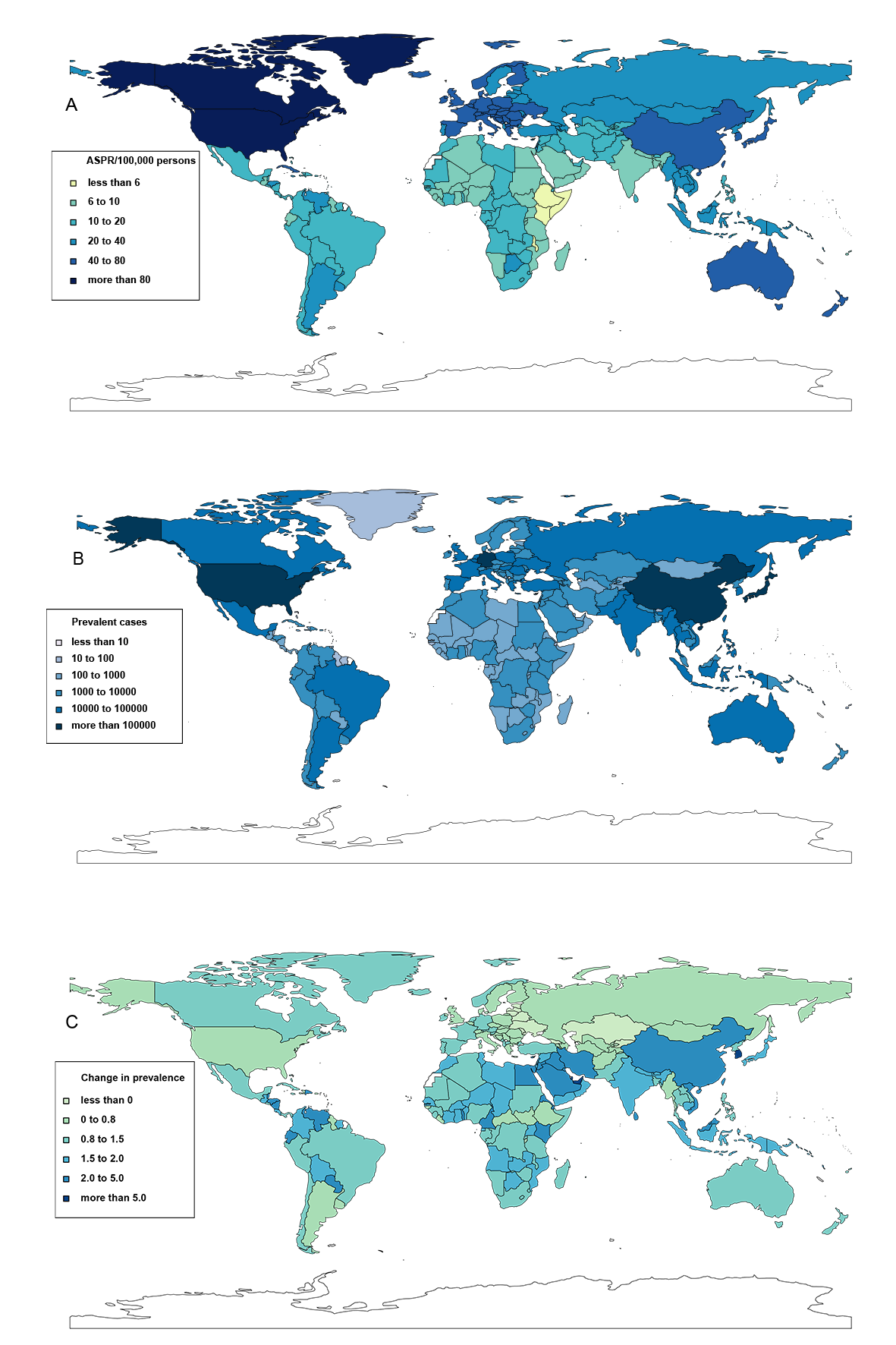

Supplement: Supplementary file 2 — Additional file 2: Figure S1. The prevalence of lungcancer for both sexes in 204 countries and territories. (A) The ASPR in 2019;(B) The prevalent cases in 2019; (C) The fold change in prevalent cases from1990 to 2019. ASPR, age-standardized prevalence rate. Figure S2.The incidence of lung cancer for both sexes in 204 countries and territories.(A) The incident cases in 2019; (B) The fold change in incident cases from 1990to 2019; (C) The ASIR in 2019. ASIR, age-standardized incidence rate. Figure S3. The YLDs of lung cancer for both sexes in 204 countries andterritories. (A) The YLDs in 2019; (B) The fold change in YLDs from 1990 to2019; (C) The ASYR in 2019. YLDs, years lived with disability; ASYR, agestandardized years lived with disability rate. [file 12889_2022_13281_MOESM2_ESM.zip › Revised Fig. S1.tif]

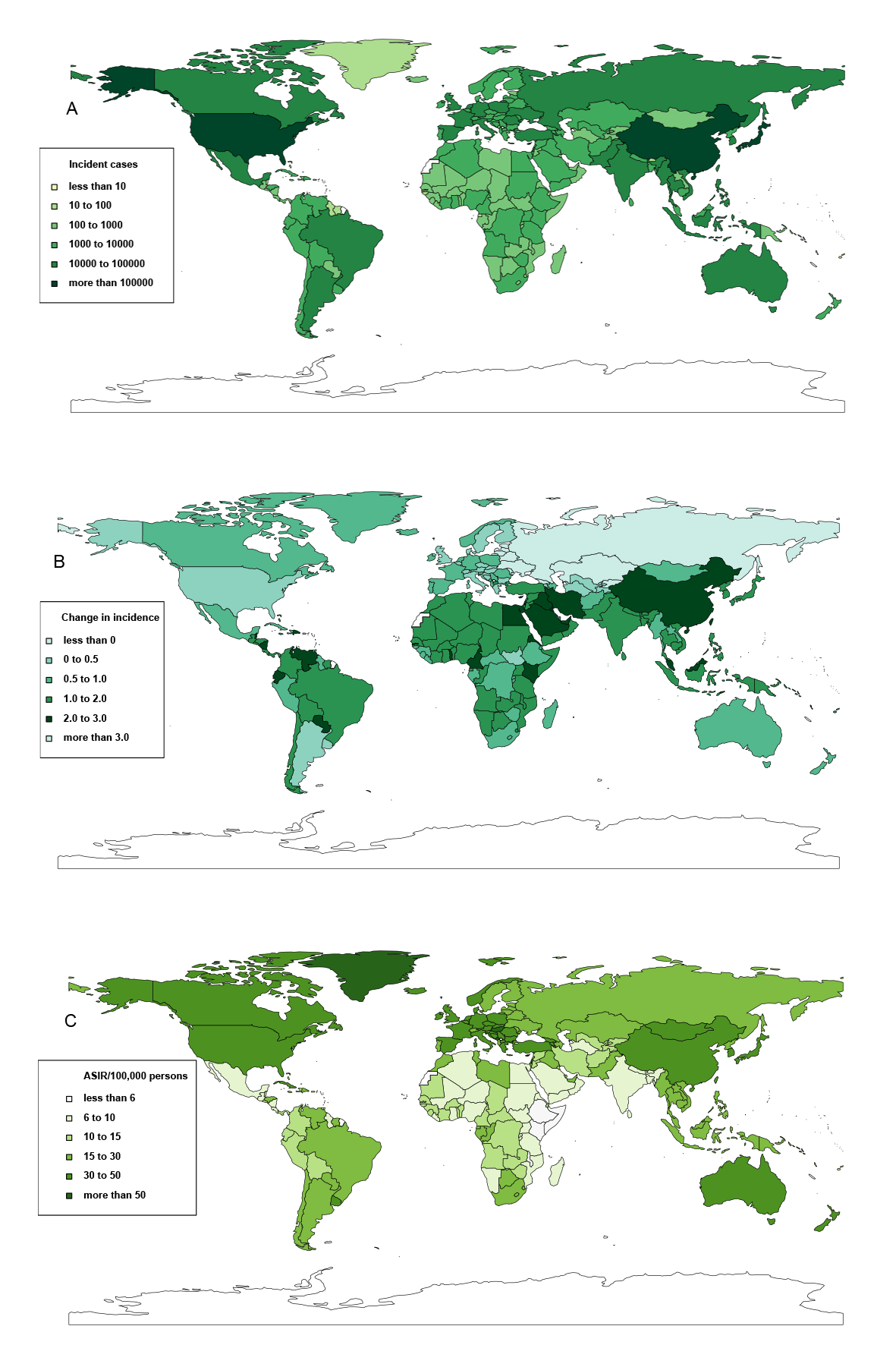

Supplement: Supplementary file 2 — Additional file 2: Figure S1. The prevalence of lungcancer for both sexes in 204 countries and territories. (A) The ASPR in 2019;(B) The prevalent cases in 2019; (C) The fold change in prevalent cases from1990 to 2019. ASPR, age-standardized prevalence rate. Figure S2.The incidence of lung cancer for both sexes in 204 countries and territories.(A) The incident cases in 2019; (B) The fold change in incident cases from 1990to 2019; (C) The ASIR in 2019. ASIR, age-standardized incidence rate. Figure S3. The YLDs of lung cancer for both sexes in 204 countries andterritories. (A) The YLDs in 2019; (B) The fold change in YLDs from 1990 to2019; (C) The ASYR in 2019. YLDs, years lived with disability; ASYR, agestandardized years lived with disability rate. [file 12889_2022_13281_MOESM2_ESM.zip › Revised Fig. S2.tif]

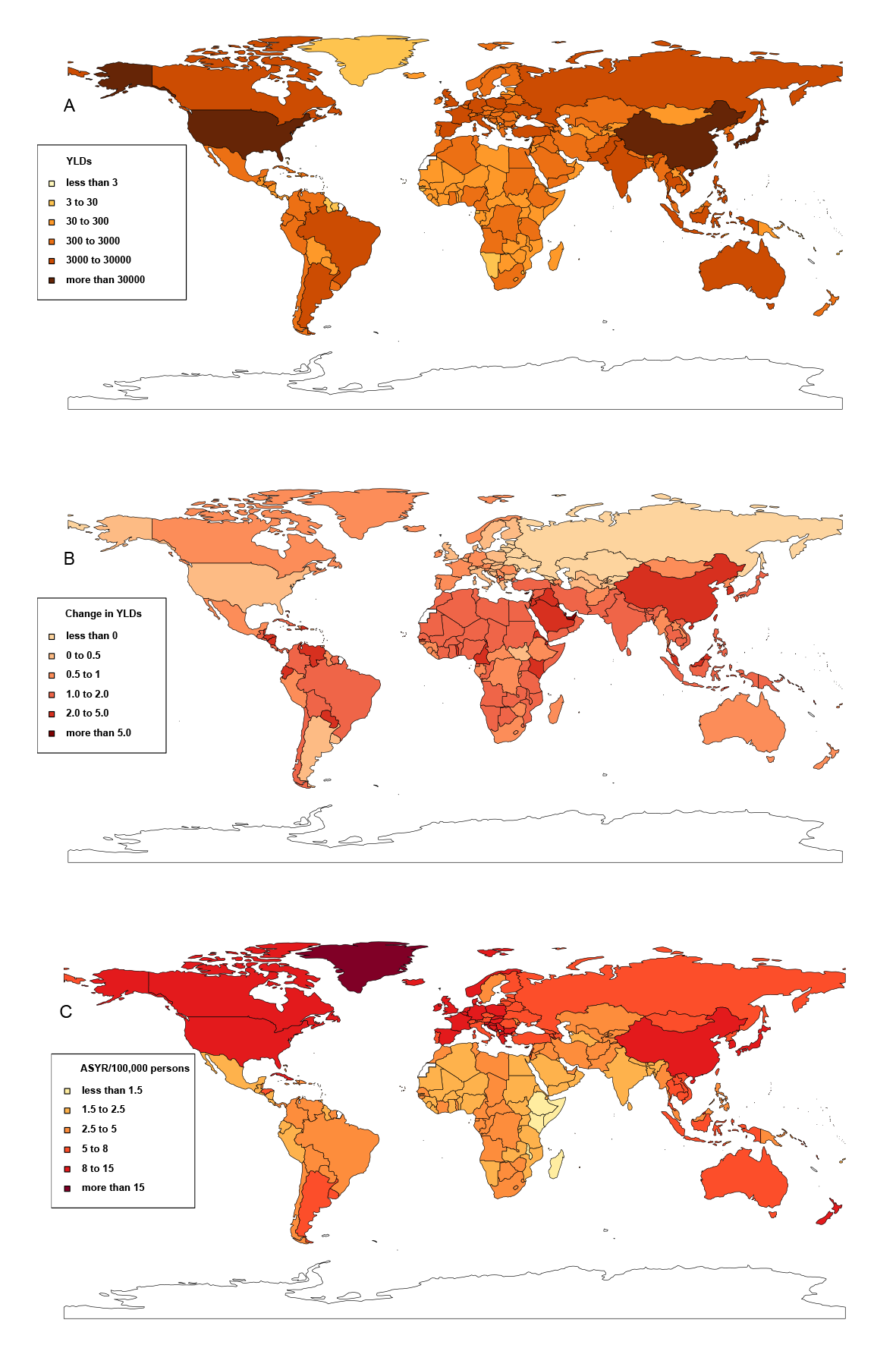

Supplement: Supplementary file 2 — Additional file 2: Figure S1. The prevalence of lungcancer for both sexes in 204 countries and territories. (A) The ASPR in 2019;(B) The prevalent cases in 2019; (C) The fold change in prevalent cases from1990 to 2019. ASPR, age-standardized prevalence rate. Figure S2.The incidence of lung cancer for both sexes in 204 countries and territories.(A) The incident cases in 2019; (B) The fold change in incident cases from 1990to 2019; (C) The ASIR in 2019. ASIR, age-standardized incidence rate. Figure S3. The YLDs of lung cancer for both sexes in 204 countries andterritories. (A) The YLDs in 2019; (B) The fold change in YLDs from 1990 to2019; (C) The ASYR in 2019. YLDs, years lived with disability; ASYR, agestandardized years lived with disability rate. [file 12889_2022_13281_MOESM2_ESM.zip › Revised Fig. S3.tif]
